# CRISPR-Cas Defense System and Potential Prophages in Cyanobacteria Associated with the Coral Black Band Disease

**DOI:** 10.3389/fmicb.2016.02077

**Published:** 2016-12-22

**Authors:** Patrick Buerger, Elisha M. Wood-Charlson, Karen D. Weynberg, Bette L. Willis, Madeleine J. H. van Oppen

**Affiliations:** ^1^Australian Institute of Marine Science (AIMS), TownsvilleQLD, Australia; ^2^Australian Institute of Marine Science, James Cook University (AIMS@JCU), TownsvilleQLD, Australia; ^3^College of Science and Engineering, James Cook University (JCU), TownsvilleQLD, Australia; ^4^Center for Microbial Oceanography: Research and Education, University of Hawaii, HonoluluHI, USA; ^5^Australian Research Council (ARC) Centre of Excellence for Coral Reef Studies, College of Science and Engineering, TownsvilleQLD, Australia; ^6^School of BioSciences, University of Melbourne, MelbourneVIC, Australia

**Keywords:** BBD, bacteriophage, viruses, pathogenicity, virulence

## Abstract

Understanding how pathogens maintain their virulence is critical to developing tools to mitigate disease in animal populations. We sequenced and assembled the first draft genome of *Roseofilum reptotaenium* AO1, the dominant cyanobacterium underlying pathogenicity of the virulent coral black band disease (BBD), and analyzed parts of the BBD-associated *Geitlerinema* sp. BBD_1991 genome *in silico*. Both cyanobacteria are equipped with an adaptive, heritable clustered regularly interspaced short palindromic repeats (CRISPR)-Cas defense system type I-D and have potential virulence genes located within several prophage regions. The defense system helps to prevent infection by viruses and mobile genetic elements via identification of short fingerprints of the intruding DNA, which are stored as templates in the bacterial genome, in so-called “CRISPRs.” Analysis of CRISPR target sequences (protospacers) revealed an unusually high number of self-targeting spacers in *R. reptotaenium* AO1 and extraordinary long CRIPSR arrays of up to 260 spacers in *Geitlerinema* sp. BBD_1991. The self-targeting spacers are unlikely to be a form of autoimmunity; instead these target an incomplete lysogenic bacteriophage. Lysogenic virus induction experiments with mitomycin C and UV light did not reveal an actively replicating virus population in *R. reptotaenium* AO1 cultures, suggesting that phage functionality is compromised or excision could be blocked by the CRISPR-Cas system. Potential prophages were identified in three regions of *R. reptotaenium* AO1 and five regions of *Geitlerinema* sp. BBD_1991, containing putative BBD relevant virulence genes, such as an NAD-dependent epimerase/dehydratase (a homolog in terms of functionality to the third and fourth most expressed gene in BBD), lysozyme/metalloendopeptidases and other lipopolysaccharide modification genes. To date, viruses have not been considered to be a component of the BBD consortium or a contributor to the virulence of *R. reptotaenium* AO1 and *Geitlerinema* sp. BBD_1991. We suggest that the presence of virulence genes in potential prophage regions, and the CRISPR-Cas defense systems are evidence of an arms race between the respective cyanobacteria and their bacteriophage predators. The presence of such a defense system likely reduces the number of successful bacteriophage infections and mortality in the cyanobacteria, facilitating the progress of BBD.

## Introduction

Diseases have become a major contributor to coral mortality over the last few decades (Willis et al., 2004; Rosenberg et al., 2007; Bourne et al., 2009). Many disease outbreaks can be linked to environmental and biological stressors, including direct human activities (Aeby et al., 2011; Lamb and Willis, 2011; Lamb et al., 2014), elevated seawater temperatures (Bruno et al., 2007; Maynard et al., 2015), and decreasing water quality (reviewed in Sutherland et al., 2004). Environmental perturbations are expected to increase in severity and frequency over time and are predicted to exacerbate the prevalence of coral diseases in the future (Selig et al., 2006; Aeby et al., 2011). While around 20 coral diseases have been described since the early 1970s, our understanding of their causative agents and pathogenesis is still limited (Harvell et al., 2007; Sheridan et al., 2013). Only a profound understanding of disease etiologies and diagnostics will lead to effective and adequate mitigation strategies (Pollock et al., 2011).

**FIGURE 1:**
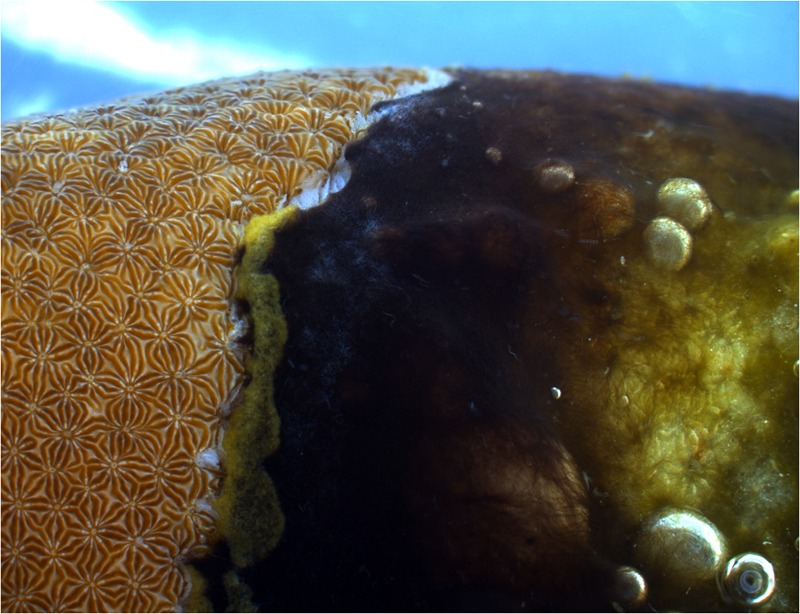
**Black band disease (BBD) on the coral *Pavona duerdeni*.** The black band microbial mat is dominated by the cyanobacterium *Roseofilum reptotaenium.* The band kills the underlying coral tissue and progresses at rates typically in the order of 3–4 mm per day.

Black band disease (BBD) is one of the earliest described coral diseases (Antonius, 1973) and the most widely reported (Green and Bruckner, 2000; Page and Willis, 2006; Barneah et al., 2007; Aeby et al., 2015; Johan et al., 2015). The disease consists of a microbial consortium of cyanobacterial species, sulfate -reducing and -oxidizing bacteria, *Alphaproteobacteria, Cytophaga*, as well as other heterotrophic bacteria (Cooney et al., 2002; Miller and Richardson, 2011; Sato et al., 2011). The bacteria form a black mat (**Figure 1**) that is characterized by an anoxic, sulfide gradient towards the coral surface, which is lethal for the underlying coral tissue, and that progresses over the colony surface at rates up to 2 cm per day (reviewed by Sato et al., 2016). In terms of biomass, most abundant in the disease mat is a filamentous cyanobacterium, *Roseofilum reptotaenium* (Rasoulouniriana) Casamatta (Rasoulouniriana et al., 2009; Casamatta et al., 2012; Buerger et al., 2016). The cyanobacterium is thought to be one of the main pathogens within the BBD microbial consortium because of its ability to penetrate polyp tissues and gastrovascular cavities (Kramarsky-Winter et al., 2014; Richardson et al., 2014), thereby providing a framework for disease development (Sato et al., 2016). It also supplies energy and nutrients to the microbial consortium, possibly through fermentation and photosynthesis in the sulfide-rich BBD environment, as suggested for *Geitlerinema* sp. BBD_1991 (Den Uyl et al., 2016). However, important questions, such as the direct cause of BBD onset and progression drivers, remain unclear (Sato et al., 2016). Although the role of viruses, in particular bacteriophages, can provide new insights into the drivers and causation of a coral disease (Weynberg et al., 2015), BBD etiology has most commonly been studied by investigating the functions of associated bacteria, while the role of bacteriophages has not been considered.

Lysogenic bacteriophages integrate into the genome of an infected bacterium and can change the bacterium’s behavior and virulence through the newly introduced genetic material (Brüssow et al., 2004). For example, the pathogenicity of *Vibrio cholerae* primarily depends on infection by a lysogenic bacteriophage (CTXphi). The bacteriophage transfers genes that encode for one of the primary virulence factors, such as the cholera toxin (CT), and converts *V. cholerae* from a non-pathogenic to a pathogenic strain (Waldor and Mekalanos, 1996; Faruque and Mekalanos, 2003). Lysogenic conversion may also transform *Vibrio coralliilyticus* into a coral pathogen triggering the coral disease white syndrome, since some of its virulence factors are found on pathogenicity islands that contain toxin genes homologous to those of the *V. cholerae* prophage (Weynberg et al., 2015).

In addition to triggering diseases through lysogenic conversion, bacteriophages can also lyse bacteria and mitigate bacterial diseases through their lytic cycle. The lytic ability of phages has been harnessed by humans to treat disease, known as ‘phage therapy’ (Duckworth and Gulig, 2002). Phage therapy has been successfully used to treat human diseases in clinical trials (reviewed in Abedon et al., 2011), mitigate diseases in aquaculture (reviewed in Oliveira et al., 2012), and to treat coral diseases (Efrony et al., 2007; Atad et al., 2012; Cohen et al., 2013). The bacteriophage infects the target host, takes over the cell machinery, replicates itself, lyses the host bacterium, and subsequently spreads its progeny phages, which infect remaining host cells and mitigate the disease over a short period of time (d’Herelle, 1930; Adams, 1959).

Conversely, bacteria can prevent bacteriophage infection by maintaining defense mechanisms, such as ‘clustered regularly interspaced short palindromic repeats’ (CRISPR) associated systems. Short sequences (spacers) that match the foreign target DNA and RNA sequences (protospacers) are stored in repetitive regions, known as CRISPR arrays (reviewed in Makarova et al., 2011a,b). An operon of associated genes codes for proteins (Cas) that detect and cleave the foreign DNA, guided by the transcribed spacers (crRNAs). CRISPR-Cas systems are known to be prevalent in ∼10% of currently sequenced bacterial genomes (Burstein et al., 2016), but seem to be more widespread among cyanobacterial genomes (detected in 68.3%, 86 out of 126 cyanobacterial genomes, Cai et al., 2013).

Evidence of typically high bacteriophage densities in cyanobacterial mats (Hennes et al., 1995; Voorhies et al., 2016), and the generally rapid co-evolution between hosts and their associated viruses in microbial mats (Heidelberg et al., 2009), led us to hypothesize that cyanobacterium-specific bacteriophages are likely to be present in BBD. In this study, we test for the existence of interactions between bacteriophages and the main BBD cyanobacteria, *R*. *reptotaenium* and *Geitlerinema* sp. BBD_1991. We evaluate the likelihood that the cyanobacteria are target of bacteriophage predation, which could influence their virulence and the development of BBD pathogenicity. By sequencing the genome of *R. reptotaenium* AO1 (Buerger et al., 2016) and retrieving the available genome for *Geitlerinema* sp. BBD_1991, we analyze the two data sets *in silico* for prophage integrations, bacteriophage–host interactions, and host defense mechanisms against virus infections. We also tested cultures of *R*. *reptotaenium* AO1 for evidence of active lysogenic infection, which was suggested from our genomic results, using mitomycin C and UV treatments.

## Materials and Methods

### Coral Sampling and Cyanobacteria Culturing

Samples of the coral *Pavona duerdeni* were collected at Orpheus Island in the central Great Barrier Reef (S 18-34.609; E 146-29.793) in 3–5 m water depth, transported to the Australian Institute of Marine Science (Townsville), and kept in flow-through aquaria until further processing. The main BBD-associated cyanobacterium, *R. reptotaenium* (Rasoulouniriana) Casamatta, was isolated and cultured as described elsewhere (Buerger et al., 2016). In brief, ∼1 cm^2^ of the black band mat was removed from an infected coral, inoculated onto an agar plate (0.6% bacteriological agar in autoclaved seawater, enriched with L1 medium at a final 1x concentration), and placed under unidirectional light of 40 PAR in 30°C. After 6 h of incubation, cyanobacteria furthest away from the inoculation spot were transferred to a new agar plate. Incubation steps were repeated three times until cyanobacteria were clean of contaminating bacteria. Non-axenic monocultures (single cyanobacterial genotype with associated bacteria) were obtained by isolating a single cyanobacterium from an agar plate under a stereo-microscope (Wild 143 Heerbrugg, M3Z, Switzerland). Cyanobacteria were grown in 125 mL L1 medium, with a 12 h light cycle until the end of their exponential phase, at which time a few aggregations of filaments were taken for DNA extraction.

### DNA-Extraction and Sequencing

DNA was extracted from 50 mg (dry weight) samples of the cyanobacterial biomass (*n* = 2 samples) with a Mo-Bio Power Plant Pro DNA extraction kit (cat. 13400-50), according to the manufacturer’s recommendations, with the following small modifications. Samples were: (1) bead-beaten in Power Plant Pro kit solution PD1 (450 μl), PD2 (50 μl), and Rnase A (3 μl, 25 mg/ml) for 1 min at max speed with (Fastprep-25 5G, MP Biomedicals); (2) incubated for 1 h at 56°C and 10 min at 65°C with proteinase k (15 μl, 20 mg/ml); and (3) eluted with 2 × 50 μl TE (10 mM Tris/Hcl, pH 8.5, 0.1 mM EDTA). Approximately 2.5 μg of purified DNA (Zymo genomic DNA clean and concentrator) was sent for next generation sequencing with a Truseq library preparation on a MiSeq 2 × 300 V3 (Ramaciotti Centre, University of New South Wales).

### Genome Assembly and Annotation

Paired-end sequences were merged with PEAR 0.9.5, using default parameters. Low quality reads (phred score < 33 within 95% of the sequence) identified by Fastx v0.0.13 and below 100 bp were removed. Reads were assembled into contigs with SPAdes 3.5.0, k-mer range = 55, 99, 127 (Bankevich et al., 2012). Contig bins were created based on marker genes, nucleotide composition, and contig abundance (minimum contig length 1000 bp) with MaxBin-1.4.2 (Wu et al., 2014), and taxonomically identified to genus level using Kraken v0.10.5-beta (Wood and Salzberg, 2014). A circular genome view was created with the software CGView (Stothard and Wishart, 2005). Assemblies were annotated using RAST (Rapid Annotations using Subsystems Technology, Overbeek et al., 2014), submitted to NCBI GenBank (accession numbers in results section and Supplementary Table S1) and filtered for genes that were relevant for bacteriophages (weblinks for bioinformatics tools, Supplementary Table S1). An additional data set was retrieved from *Geitlerinema* sp. BBD_1991 (Den Uyl et al., 2016) and compared to the results from the *in silico R*. *reptotaenium* AO1 analyses.

### CRISPRs

Clustered regularly interspaced short palindromic repeats (CRISPR), associated Cas genes, and direct repeats were identified within the respective cyanobacterium genome bins of *R*. *reptotaenium* AO1 (Buerger et al., 2016) and *Geitlerinema* sp. BBD_1991 (Den Uyl et al., 2016) with CRISPRfinder, CRISPRdb and CRISPRcompar (Grissa et al., 2007). Only confirmed CRISPR arrays were considered. To assess protospacers and potential self-targeting spacers, CRISPR arrays were compared to: (a) publicly available databases: viral RefSeq, plasmid RefSeq and GenBank-phage accessed through CRISPRtarget online tool, as well as (b) local databases of the assembled genomic bins, by retrieving best possible BLASTn matches with default parameters for short sequences (-gapopen 10, -gapextend 2, -dust no, -reward 1, -penalty -1, -word_size 7, -qcov_hsp_perc 100, Biswas et al., 2013). For both analyses, only protospacers with the CRISPRtarget default minimum matching score of 20 were considered as a possible categorical match (bacteriophage, plasmid, unknown). A less stringent matching score (i.e., 18 and 19) was only considered if protospacer matches were relevant to the respective environment (e.g., cyanophage). Cas gene assignments, self-targeted genes, and open reading frames adjacent to CRISPR arrays were verified against the NCBI nr database to known protein sequences (tBLASTx) using Artemis (version 16, Rutherford et al., 2000).

### Prophage

The assembled cyanobacterium genome contigs of *R*. *reptotaenium* AO1 (Buerger et al., 2016) and *Geitlerinema* sp. BBD_1991 (Den Uyl et al., 2016) were analyzed for prophage gene signatures with PHAST (Zhou et al., 2011), PHASTER (Arndt et al., 2016) and VIRsorter (Roux et al., 2015), using default parameters for submission as metagenomic contigs. Although incomplete prophages are reported in this paper, only complete prophage signatures were considered as potentially functional prophages. Annotations of potential prophage regions were checked for unrecognized phage genes, toxicity genes and genes of virulence with Blast2GO against the Swissprot database (Conesa and Götz, 2008) and BLASTp against the NCBI nr database.

### Lysogenic Virus Induction

*R. reptotaenium* AO1 was grown at 30°C with 50–80 μE m^-2^ s^-1^ light intensity in 12 well plates until mid-exponential phase (Buerger et al., 2016) and then exposed to a mitomycin C treatment to induce virus replication of potential lysogenic bacteriophages. Mitomycin C was added to the cyanobacterial cultures (exposure time 2 h) in final concentrations of 0.1, 0.5, and 1 μg ml^-1^ (three wells per concentration, three plate replicates). After the 2-h exposure, mitomycin C-treated medium was replaced by fresh L1 growth medium and plates were maintained at control incubation conditions (Paul and Weinbauer, 2010). In a separate experiment, cyanobacteria were exposed to UVA and UVB light (intensity 2.5 mW/cm^2^) for 1, 2, 4, 10, 15, 20, and 30 min. After UV exposure, cultures were kept at control incubation conditions. Cyanobacterial growth was monitored for potential lysis with fluorescence readings over time (Synergy H4, Biotek). Fluorescence readings were averaged from an area scan, with 25 reads per well, and transformed into a measure of cyanobacterial biomass (Buerger et al., 2016).

To quantify viral abundance in each treatment well, the abundance of virus-like particles (VLPs) was measured on a flow cytometer (BD FACSVerse) with a 488 nm argon-ion laser (Brussaard et al., 2010). In brief, samples were fixed in the dark at a final concentration of 4% μl glutaraldehyde, incubated for 30 min at 4°C, and stained with 1x SYBR green I (Invitrogen) at 80°C for 10 min. Samples were counted in a dilution series at a low flow rate with approximately 200–400 events per second, same VLP standards as Pollock et al. (2014).

## Results

### Assembly and Gene Annotation

The sequences retrieved from the *R*. *reptotaenium* culture were assembled using the software SPAdes 3.5.0 and resulted in three genomic bins that were submitted for automated annotation with RAST. The three bins were taxonomically identified as cyanobacterium *R. reptotaenium* AO1 (RAST 564709.3; NCBI GenBank project accession number MLAW00000000), *Alphaproteobacterium* (RAST 28211.29, and *Cytophagaceae* sp. (RAST 89373.4). The *R*. *reptotaenium* AO1 genome bin matched our expected BBD cyanobacterial target and was chosen for further analyses. The *R*. *reptotaenium* AO1 genome bin consisted of 134 contigs, with a total length of 5,826,181 bp (**Table 1**). The full annotation contained 5,491 features, with 39 possible missing genes (data access, RAST and NCBI GenBank, Supplementary Table S1).

**Table 1:** Assembly details of the draft genome of *Roseofilum reptotaenium* AO1.

Parameter	Assembly details
Sequencing library	True Seq library
Sequencing platform	MiSeq 2x300 V3
Assembly software	SPAdes 3.5.0
K-mer length	55–127
Total number of sequences	134
Total length [bp]	5,826,181
Shortest sequence [bp]	1032
Longest sequence [bp]	308,535
Total number of Ns	305
N50	94,947 (20 sequences)
GC content [%]	44.81
Coverage	300–600×

*Bioinformatic statistics such as N50 values and sequence length were generated with the software gnx-tools (version 0.1+20120305).*

### CRISPRs

**FIGURE 2:**
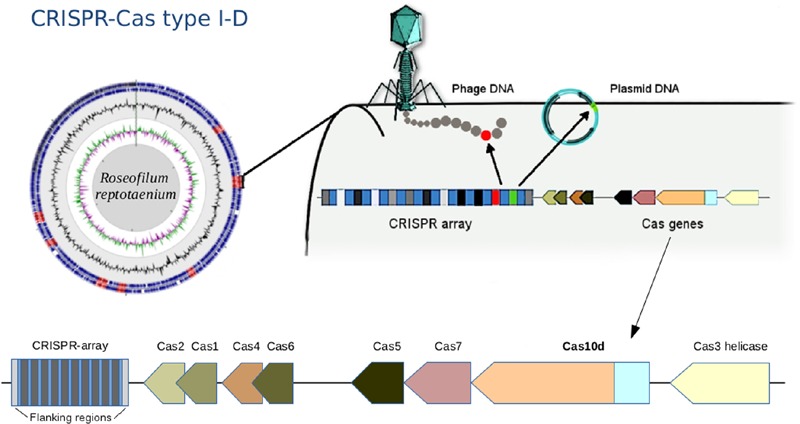
**Draft genome assembly and illustration of the CRISPR-Cas system in *Roseofilum reptotaenium* AO1.** The adaptive, heritable CRISPR-Cas system defends the cyanobacterium against bacteriophage infections, plasmids and mobile genetic elements. The gene Cas10d is representative of the CRISPR-type I-D, commonly found in other cyanobacteria. CRISPR arrays are marked in red on the genome contigs, displayed in a circular view. The Cas gene arrangement is indicated at the bottom of the figure.

A CRISPR-Cas immune system type I-D, also known as CASCADE (CRISPR associated complex for antiviral defense), was identified within the *R. reptotaenium* AO1 genome (**Figure 2**). CRISPR type I systems are known to target DNA only, not RNA, and require a specific protospacer-adjacent motif (PAM) on the target sequence next to the protospacer to be functional (reviewed in Jiang and Marraffini, 2015). The associated genes, adjacent to CRISPR array #2, were homologs of the known Cas genes, such as Cas3 helicase, Cas10d, Cas7, Cas5, Cas6, Cas2, and Cas1 (respective functions in Supplementary Table S2, modified from Makarova et al., 2011a,b). Compared to other cyanobacterium genomes, the *R. reptotaenium* AO1 CRISPR-Cas system is of average complexity, with seven array loci and a total of 100 spacers (median of type I systems contain approximately 92 spacers and three CRISPR loci; Cai et al., 2013).

Horizontal gene transfer has been inferred as one of the main methods for distributing parts of the CRISPR-Cas systems among bacteria, based on high sequence similarity of direct repeats (Godde and Bickerton, 2006). In *R. reptotaenium* AO1, all direct repeats of the CRISPR arrays were 37 bp long (**Table 2**) and showed high similarity to direct repeats of other cyanobacterial species, such as the marine *Rivularia* sp. PCC 7116 strain (Supplementary Table S3). The respective CRISPR-flanking regions were found to be specific for *R. reptotaenium* AO1, with no BLAST homologies to other known sequences.

**Table 2:** CRISPR-Cas spacers of *R. reptotaenium* AO1 and *Geitlerinema* sp. BBD_1991.

CRISPR array # *R. reptotaenium* AO1	Position 1st CRISPR, contig number	length [bp]	DR length [bp]	Spacers #	Spacer average length [bp]
1	171508..171547, contig_15	1518	37	20	37.1
2 + Cas I-D	446355..446395, contig_18	1071	37	14	36.9
3	2179117..2179156, contig_37	933	37	12	37.8
4	2351308..2351339, contig_38	553	37	7	36.9
5	3024497..3024532, contig_46	844	37	11	36.5
6	3582097..3582130, contig_55	2022	37	27	36.6
7	4115583..4115616, contig_65	693	37	9	36.0
Total		Average	Average	Sum	Total average
7		1091	37	100	36.8

**CRISPR array # *Geitlerinema* sp. BBD_1991**	**Position 1st CRISPR, contig number**	**length [bp]**	**DR length [bp]**	**Spacers #**	**Spacer average length [bp]**

1 + Cas I-D	384474..384507, BBD_1000996	19166	37	260	37.6
2	1172325..1172357, BBD_1000999	7340	37	100	36.0
3 + Cas III-U	1589539..1589575, BBD_1001002	7461	37	101	36.5
4 + Cas III-U	1597098..1597137, BBD_1001002	4589	37	62	36.4
5 + Cas I-MYXAN	2022334..2022369, BBD_1001004	2114	36	29	35.7
6 + Cas III-B	2315188..2315226, BBD_1001007	5320	36	71	38.4
7 + Cas genes	2341519..2341555, BBD_1001007	1725	35	23	38.5
8 + Cas genes	2577530..2577580, BBD_1001010	246	25	3	48.7
9	3423177..3423219, BBD_1001016	243	30	3	41.0
10 + Cas genes	4126767..4126803, BBD_1001024	985	35	13	38.1
11 + Cas genes	4127858..4127894, BBD_1001024	1568	35	21	38.0
Total		Average	Average	Sum	Total average
11		4614	34.5	686	38.5

*The total length of the CRISPR array is given from the start to the end of the respective direct repeats (DR). Only complete CRISPR arrays were considered. CDS numbers refer to overall nucleotides with contigs names in sequence.*

In total, seven CRISPR arrays containing a total of 100 unique spacers were identified in *R. reptotaenium* AO1. Identical matches (protospacers) to eight spacer sequences were found on one cyanobacterial genome contig (contig 93, cov. 558, length 31,342 bp). The protospacers on contig 93 match spacers in conserved middle parts of the CRISPR arrays (average spacer position: 8.75 out of 15.88, Supplementary Table S4). One of the target regions on contig 93 codes for a DnaB domain-containing helicase (related to a bacteriophage multi-domain, phage_DnaB, BLASTp *e*-value < 6.28*e* - 20), while all others code for hypothetical proteins (Supplementary Table S4). Analysis of the assembly’s de Bruijn graphs showed that contig 93 is not circular shaped, but can be connected to other contigs, and is therefore not part of a plasmid or linear extra-chromosomal element (Supplementary Figure S1). Contig 93 was not integrated into other contigs, likely due to multiple connection possibilities, strain variations, or repetitive regions. Although a phage integrase was detected on one of the adjacent contigs (contig 184, pfam00589, *e*-value < 8.47*e* - 03, Supplementary Table S5), no other genes related to bacteriophages or mobile genetic elements were uncovered.

Results from protospacer matches to public databases (40% of spacers had potential protospacer matches) showed that protospacers were mostly unrelated to cyanobacteria or the marine environment (Supplementary Table S6), indicating that spacer sequences were novel and not represented in publicly available databases. Protospacer origins were non-redundant and matched to a diversity of plasmids and viruses, with multiple hits to *Synechococcus* phages, a *Cyanothece* sp. plasmid, and a *Sinorhizobium fredii* plasmid (number of hits were 3, 2, and 2, respectively, Supplementary Table S6).

**FIGURE 3:**
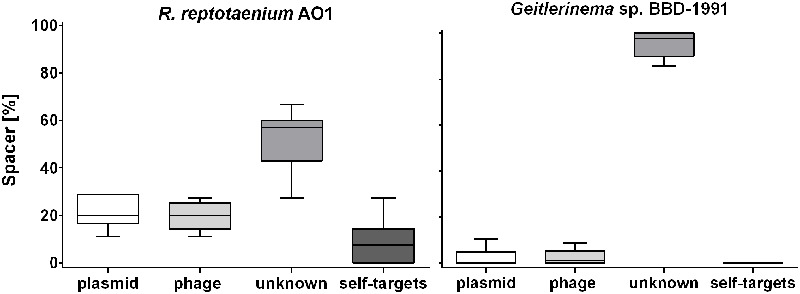
**Potential CRISPR-Cas spacer targets within BBD.** Most of the spacer targets (protospacers) could not be identified by matches to CRISPR sequences in publicly available databases. An unusually high number of protospacers were located within a short region of the *R*. *reptotaenium* AO1 genome. Spacer targets in % were calculated for the respective categories within each of the seven CRISPR arrays and visualized as replicates in a boxplot (detailed protospacer Supplementary Table S6). Bar = mean, whiskers = min to max values, box = 25th and 75th percentiles.

Eleven CRISPR arrays were detected in the genome of *Geitlerinema* sp. BBD_1991 (Den Uyl et al., 2016), which had an extraordinarily high number of spacers (*n* = 260), approximately six times as many spacer sequences compared to *R*. *reptotaenium* AO1 (**Table 2**). While most of the target sequences (protospacers) were unknowns (95% ± 6), no identical spacer sequences were identified between the two genomes and also no self-targeting spacers were found in *Geitlerinema* sp. BBD_1991 (**Figure 3**; Supplementary Table S6). Cas genes were adjacent to almost all CRISPR arrays of *Geitlerinema* sp. BBD_1991, whereas *R*. *reptotaenium* AO1 had only one CRISPR array with adjacent Cas genes (**Table 2**; Supplementary Table S2). Several genes representative of different CRISPR-Cas types were detected in *Geitlerinema* sp. BBD_1991, such as types I-D, II-U, III-B and I-MYXAN. The direct repeat sequences of *Geitlerinema* sp. BBD_1991 were similar to *R*. *reptotaenium* AO1, and closely related to other cyanobacteria species, such as *Crinalium epipsammum* and *Synechococcus* sp. (Supplementary Table S3).

### Prophage Detection

Three potential prophages were detected in *R*. *reptotaenium* AO1 (R1-R3, **Table 3**; Supplementary Tables S7 and S8). These were classified as incomplete and questionable prophages, because some of the required genes to fully form an assembled virus, such as genes coding for a tail and capsid, were missing from the contigs. It is noteworthy that the tools PHAST, PHASTER, and VIRsorter did not identify prophage signatures in the same regions of the genome, which may suggest that our ability to capture prophage signatures is still limited.

**Table 3:** Details of prophage detection.

#	Contig node #	Software	Classification score		Length [bp]	CDS	GC [%]
***R*. *reptotaenium* AO1**				
R1	contig_72	PHAST	Incomplete	20	7,100	8	45.1
		PHASTER	–	–	–	–	–
		VIRsorter	–	–	–	–	–
R2	contig_41	PHAST	–	–	–	–	–
		PHASTER	Incomplete	10	103,700	15	42.2
		VIRsorter	–	–	–	–	–
R3	contig_93	PHAST	–	–	–	–	–
		PHASTER	–	–	–	–	–
		VIRsorter	Category 3		31,342	9	44.4
							
***Geitlerinema* sp. BBD_1991**				
G1	BBD_1000996	PHAST	Incomplete	20	19,800	7	53.29
		PHASTER	Incomplete	20	19,800	7	53.21
		VIRsorter	–	–	–	–	–
G2	BBD_1001009	PHAST	Incomplete	40	9,000	10	50.07
		PHASTER	–	–	–	–	–
		VIRsorter	–	–	–	–	–
G3	BBD_1001028	PHAST	Questionable	90	16.600	16	51.12
		PHASTER	Questionable	80	16.600	16	51.01
		VIRsorter	–	–	–	–	
(G4)	BBD_1001072-4	PHAST	Questionable	80	20,900	29	49.14
		PHASTER	–	–	–	–	–
		VIRsorter	–	–	–	–	–
G5	BBD_1001065	PHAST	–	–	–	–	–
		PHASTER	–	–	–	–	–
		VIRsorter	Category 3		19,845	11	56.32

*Different tools were used for prophage detection in the draft genome of *R. reptotaenium* AO1 and *Geitlerinema* sp. BBD_1991. Gene details in Supplementary Tables S8 and S9. Classifications (PHAST, PHASTER): Intact, questionable or incomplete prophage = scoring from lowest 0 - highest 150, Table 1 in Zhou et al. (2011). VIRsorter category 3 = sequence similar to virus genome structure, but without sequence similarity to known viruses. CDS indicates the number of coding sequences. Overall GC-content *R*. *reptotaenium* AO1: 44.81%; *Geitlerinema* sp. BBD_1991: 50.38%. Annotation details in Supplementary Tables S8 and S9.*

The potential prophage regions of the *R*. *reptotaenium* AO1 genome carried genes that could be involved in processes other than virion assembly, such as the genes DNA adenine methyltransferase, transketolase, GDP-D-mannose 4,6-dehydratase, D, D-heptose 7-phosphate kinase, phosphoheptose isomerase, and a ADP-L-glycero-D-mannoheptose-6-epimerase (Supplementary Table S8). Some of these associated genes are involved in the non-oxidative pentose phosphate pathway (GDP-D-mannose 4,6-dehydratase), which provides energy during replication of virus when photosynthesis is not present (Shestakov and Karbysheva, 2015). However, potential cyanobacterial virulence factors that were located on prophage regions included lysozyme/metalloendopeptidases (e.g., region R1, e-value 7.00e - 017, Supplementary Table S8), genes potentially involved in lipopolysaccharide production (phosphoheptose isomerase, ADP-L-glycero-D-mannoheptose-6-epimerase and an NAD-dependent epimerase/dehydratase in region R2, e-value 3.37e - 029, Supplementary Table S8). Genes related to bacteriophages functionality were detected by RAST outside potential prophage regions and scattered across the *R*. *reptotaenium* AO1 contigs, such as a phage endolysin gene, phage tail protein, a T4-like virus tail tube protein gp19, a phage associated DNA primase, a putative prophage protein, a phage shock protein and a phage integrase (Supplementary Table S7).

By comparison, five potential prophages were detected in *Geitlerinema* sp. BBD_1991 (**Table 3**; Supplementary Table S9) (Den Uyl et al., 2016). Prophage_G1 region, recognized by PHAST and PHASTER (not VIRsorter), was the only region flanked by integration sites AttL and AttR, but was missing phage-related assembly genes. Phage-related genes were detected by PHAST and PHASTER mainly in the regions G3, resulting in a high prediction score (scored 90, questionable prophage) including genes such as phage baseplate, tail tube and tail sheath. In addition, the prophage_G4 region stretched over three consecutive contigs (BBD_1001072–BBD_1001074) that were not necessarily connected to each other in the genomic assembly and therefore likely represent a false positive result. Nevertheless, a gap-less assembly may rearrange the respective contig connections into a fully intact prophage. Genes directly located within the *Geitlerinema* sp. BBD_1991 potential prophage regions (Supplementary Table S9), which are likely to be involved in other processes than phage assembly, include photosystem II components, such as PsbE, PsbF, and PsbJ (prophage_G1), genes that contribute to Fe(II) transport systems, such as iron permease FTR1, to a non-oxidative pentose phosphate pathway, such as the GDP-D-mannose 4,6-dehydratase and other genes, such as a phosphoribosylglycinamide formyltransferase, orotate phosphoribosyltransferase, guanine deaminase and a biotin-(acetyl-CoA-carboxylase) ligase BirA. Potential virulence genes located in *Geitlerinema* sp. BBD_1991 prophage regions include a lysozyme/metalloendopeptidase (region G3 and G4, see annotations Supplementary Table S1 in Den Uyl et al., 2016), a peptidase S8/S53 (subtilase family protease, PHAST in region G4) and lipopolysaccharide modification genes, such as GDP-L-fucose synthase and glycosyl transferase (PHAST in region G4).

### Lysogenic Virus Induction

**FIGURE 4:**
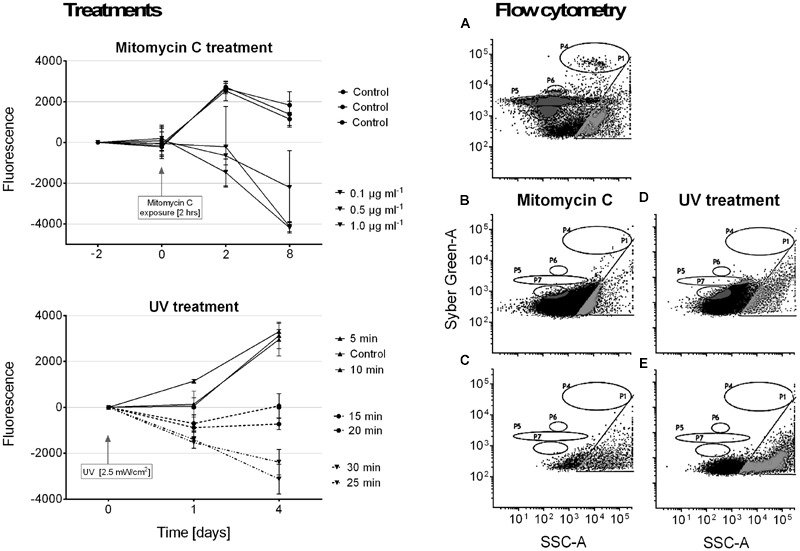
**Results of lysogenic virus induction experiments using mitomycin C and UV light treatments.** Panel on the left shows cyanobacterial growth, measured as fluorescence, in response to the respective treatments. Samples were normalized by calculating differences from starting values. Panels on the right show examples of particle size distributions from flow cytometry measurements after treatment (quantification not included due to no hits in relevant areas). In accordance with measured known references (Pollock et al., 2014), gate P4 = bacteria populations, and gates P5–P7 = virus like particles (VLPs). **(A)** Reference plot of bacteria and virus populations, **(B)** 2 h mitomycin C exposure at 1 μg/ml, **(C)** mitomycin C control measurement, **(D)** 30 min UV exposure, **(E)** UV treatment control measurement.

It was not possible to induce a lysogenic virus from our *R. reptotaenium* AO1 cultures. Although cyanobacterial cell densities in cultures declined over time in response to the mitomycin C and UV treatments, we did not detect any evidence of bacteriophage replication in our flow cytometry counts (**Figure 4**). We interpret this to indicate that the mitomycin C and UV treatments caused the cyanobacteria to disintegrate and to reduce their fluorescence compared to controls.

## Discussion

Our analyses reveal that the genome of the cyanobacterium *R. reptotaenium* AO1, the dominant member of the microbial consortium causing BBD in corals, and that of *Geitlerinema* sp. BBD_1991 are equipped with a CRISPR-Cas adaptive defense system and several prophage regions that contain potential virulence-related genes. CRISPR-Cas systems thrive in environments where there is a high incidence of phage predation (Jiang et al., 2013), and can be multi-functional, ranging from preventing bacteriophage infections, interfering with the uptake of plasmids and mobile genetic elements (Barrangou et al., 2007), to the control of gene expression (Hatoum-Aslan and Marraffini, 2014) and support of DNA repair mechanisms (Babu et al., 2011).

### CRISPR-Cas Self-targets

We found that a section of the *R. reptotaenium* AO1 genome is targeted by an unusually high number of spacer sequences (8 out of 100 spacers, Supplementary Table S6). Although spacer sequences of the CRISPR-Cas systems are usually thought to target foreign genetic material, approximately 1 in 250 spacer sequences can be self-targeting and match to particular regions of the host’s genome (Stern et al., 2010). Typically, a large proportion of these self-targeting spacers are located within the first or second position of a CRISPR array and may be acquired accidentally, resulting in autoimmunity through digestion of the host’s genetic code or inactivation of the CRISPR array (Stern et al., 2010). However, the self-targeting CRISPR-Cas spacers of *R*. *reptotaenium* AO1 do not result in autoimmunity and are not inactivated for the following reasons. The presence of the self-targeting spacers is unlikely to be accidental, because they originate from relatively conserved middle parts of the CRISPR arrays (average spacer position: 8.75 out of 15.88, Supplementary Table S4). They target loci that are not randomly distributed across the genome, but located in a narrow region of 31,342 bp (contig 93). The CRISPR arrays are still active, because up to three self-targeting spacers are present on single CRISPR arrays, indicating that they were acquired as separate events. These results suggest a secondary regulatory role of this CRISPR-Cas system in which cleavage of the host genome is prevented, possibly by the lack of PAM recognition sequences on the target regions or through another unknown process.

### Contig 93, a Potential Prophage

In *R. reptotaenium* AO1, contig 93 was detected to be an incomplete prophage by VIRsorter (**Table 2**, Supplementary Table S8). Self-targeting spacers are known to target environmental lysogenic bacteriophages or prophages of other bacteria, but rarely their own prophage signatures (Touchon and Rocha, 2010; Hargreaves et al., 2014; Briner et al., 2015). The potential prophage is classified as incomplete, because only a single gene (DnaB domain-containing helicase) was detected as having a phage origin (Phage_cluster_71 PFAM-AAA_25, coding for a DNA repair protein), while the contig lacks essential genes for virus replication and assembly, such as capsid, head, or tail genes. It is possible that essential parts of the potential bacteriophage could be spread over multiple assembled contigs. Indeed, according to the assembly de Bruijn graphs (Supplementary Figure S1), contig 93 was separated from other contigs due to multiple possibilities for continued assembly and probably unresolved repetitive regions. However, the only bacteriophage-related gene on adjacent contigs is a phage integrase (contig 184, pfam00589, *e*-value < 4e–46, Supplementary Table S5), which is required for site-specific DNA excision and integration (Fogg et al., 2014).

The area of *R. reptotaenium* AO1 contig 93 could resemble a bacteriophage that is still hidden in the genome bin. To test this hypothesis, we attempted to induce a potential hidden prophage with mitomycin C and UV treatments (**Figure 4**). Although cyanobacterial biomass declined in response to the treatments, the lack of any measurable virus replication events indicates that the observed cell degradation was probably due to the respective treatments, rather than viral lysis. Alternatively, it has been shown that a CRISPR-Cas system of *Escherichia coli* can prevent prophage induction without killing the host bacterium when both processes, virus replication and CRISPR-Cas defense, are activated at similar time points (Edgar and Qimron, 2010). Although unconfirmed, the authors suggest possible regulation by promoters of cas proteins during a stress response, such as the sigma factor σ^32^ of *E*. *coli*, which could silence CRISPR activity until required in order to prevent prophage induction (Edgar and Qimron, 2010). Consequently, even if contig 93 was part of a prophage, as indicated by the presence of multiple spacers that target the incomplete prophage region, it might not have been possible to induce it.

### Functional Role of Potential Prophage Regions

Contig 93 contains predicted coding regions for 39 hypothetical proteins with unknown functions, seven of which match directly to CRISPR-Cas spacers (Supplementary Tables S4 and S6). Other known genes on contig 93 code for a Rec-D like helicase, DnaB helicase, a putative proteinase, and a DNA-damage inducible protein. CRISPR-Cas systems can be multi-functional and involved in processes other than defense against foreign genetic material, such as expression regulation of pathogenicity genes leading to increased virulence (Hatoum-Aslan and Marraffini, 2014; Louwen et al., 2014), biofilm formation (Zegans et al., 2009), and DNA repair (Babu et al., 2011). The CRISPR-Cas1 protein (YgbT) of *E. coli* is known to interact with Rec repair proteins, which can increase resistance to DNA damage (Babu et al., 2011). In *R*. *reptotaenium* AO1, the proteins encoded by contig 93 may be interacting with the CRISPR-Cas system for increased DNA repair. Such increased DNA repair would be beneficial for *R. reptotaenium* during UV-induced DNA damage, for example in high light environments, a condition that would lead to more rapid BBD progression (Sato et al., 2010). However, further research is required to tease apart alternative hypotheses about the function of the CRISPR-Cas system in *R. reptotaenium* AO1.

**FIGURE 5:**
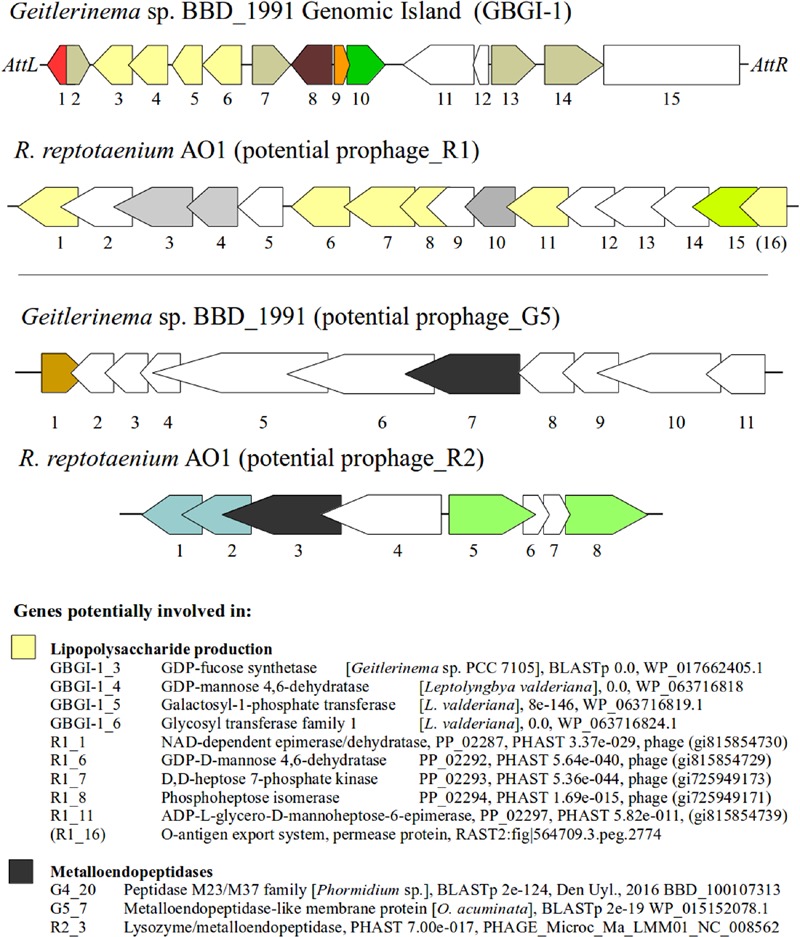
**Genetic structure of prophage regions and their potential virulence factors.** Genes with similar function are color coded, e.g., yellow = genes potentially involved in lipopolysaccharide production, dark gray = metalloendopeptidases, white = hypothetical proteins, other colors = see annotations Supplementary Tables S8 and S9. Genomic island of *Geitlerinema* sp. BBD_1991 is shown on top with the attachment sites AttL and AttR. BLASTp and PHAST results are given with *E*-values and protein accession numbers.

Several genes that represent potential virulence factors were located within other prophage regions of the *R*. *reptotaenium* AO1 and *Geitlerinema* sp. BBD_1991 genomes. These genes are likely involved in lipopolysaccharide production and code for lysozyme/metalloendopeptidases. Although some of the gene annotation differed between RAST and the prophage detection tools (PHAST and PHASTER), their functional roles were consistently associated with virulence factors of other known bacteria. The third and fourth most highly expressed cyanobacterial gene within BBD that encodes for an NAD-dependent epimerase/dehydratase (Arotsker et al., 2016) was homologous in terms of its functionality to a gene located in *R*. *reptotaenium* AO1 prophage region_R2 (PHAST annotation, Supplementary Table S8, *e*-value < 3.37*e* - 029). NAD-dependent epimerase/dehydratases have been linked to increased virulence in the bacterium *Pectobacterium carotovorum* causing soft rot disease of vegetables (Islam, 2016). *P. carotovorum* with an intact NAD-dependent epimerase/dehydratase (wcaG gene) showed increased secretion of virulence associated exoenzymes and caused 21.5–26.7% macerated tissue, while mutants with a disrupted gene caused only 5.8–6.5% tissue damage on vegetables (Islam, 2016). Since the respective NAD-dependent epimerase/dehydratase of *P. carotovorum* (Islam, 2016) and *R*. *reptotaenium* AO1 are homologs to the wcaG gene coding for a GDP-fucose synthetase, their virulence associated exoenzymes are likely to be involved in the production of lipopolysaccharides and colanic acid, both of which are known bacterial virulence factors (Fry et al., 2000). The same respective gene in *R*. *reptotaenium* AO1 prophage_R2 region has been annotated by RAST as a rhamnose containing glycans subsystem coding for an UDP-glucose 4-epimerase (EC 5.1.3.2). This particular annotation is also a known virulence factor, which is located within a gene cluster that produces endotoxic lipopolysaccharides in *Campylobacter* spp. (Fry et al., 2000). A deactivation of the UDP-glucose 4-epimerase coding gene resulted in the expression of incomplete lipopolysaccharides and a virulence reduction (Fry et al., 2000). In addition, both cyanobacterial genomes had other virulence-associated genes in potential prophage regions involved in lipopolysaccharide production (**Figure 5**). For *R*. *reptotaenium* AO1 these were: GDP-D-mannose 4,6-dehydratase (Webb et al., 2004), phosphoheptose isomerase, ADP-L-glycero-D-mannoheptose-6-epimerase and an *O*-antigen export system (Brooke and Valvano, 1996); and for *Geitlerinema* sp. BBD_1991: GDP-L-fucose synthase (Mäki and Renkonen, 2004), GDP-D-mannose 4,6-dehydratase (Webb et al., 2004), glycosyl transferase (Davies et al., 2013). In *Geitlerinema* sp. BBD_1991 some of these genes are located within prophage_G1 region, that contained an integrase (G1_1), recombinase (G1_2) and was flanked by phage attachment sites attL/attR (**Figure 5**; Supplementary Table S9), known arrangements of genomic and pathogenicity islands (Bellanger et al., 2014). Several studies have suggested that cyanobacterial lipopolysaccharides are less endotoxic than classic lipopolysaccharides (reviewed in Gemma et al., 2016). However, the high expression of NAD-dependent epimerase/dehydratase (Arotsker et al., 2016) leads to the assumption that lipopolysaccharides could play an important role in the virulence of BBD associated cyanobacteria.

Other virulence associated genes coding for lysozyme/metalloendopeptidases (superfamily peptidase M23, zinc metallopeptidases, BLASTp *e*-value < 2.23*e* - 36), were present in both genomes (prophage_R1 in Supplementary Table S8; G4 BBD_100107313 and G5 BBD_10010657 in Supplementary Table S9). Although unreported for cyanobacteria, metalloendopeptidases are known virulence factors in a wide range of bacterial pathogens causing tissue damage (Miyoshi and Shinoda, 1997).

We found potential virulence genes that had overlapping functionality in prophage regions of both genomes, *R*. *reptotaenium* AO1 and *Geitlerinema* sp. BBD_1991. To date, viruses have not been considered as a contributor to the virulence of BBD associated bacteria. Our results strongly suggest that genetic material related to lysogenic bacteriophages contributes to the virulence of *R*. *reptotaenium* AO1 and *Geitlerinema* sp. BBD_1991.

## Conclusion

Here we show that the cyanobacterium *R. reptotaenium* AO1, the dominant microbe within the BBD consortium, and *Geitlerinema* sp. BBD_1991, a less abundant member of the consortium, acquire resistance against bacteriophages by maintaining adaptive, heritable CRISPR-Cas defense systems. It is not surprising to find CRISPR-Cas systems and potential prophage regions in BBD associated cyanobacteria. Microbial mats, such as BBD, can be hot-spots for bacteriophage–host interactions due to the high abundance of bacteria and associated viruses (Heidelberg et al., 2009; Carreira et al., 2015). Cyanobacteria are the most abundant microbes in terms of biomass within the BBD consortium, and therefore a likely target for bacteriophage infections (Thingstad, 2000). Such constant phage predation pressure can result in an arms race and initiate evolution of host defense mechanisms, such as CRISPR-Cas systems (Levin, 2010; Held et al., 2013). CRISPR-Cas systems are not an absolute barrier and can be overcome by rapidly evolving bacteriophages (Andersson and Banfield, 2008), reflected by the multiple potential prophage regions within the *R. reptotaenium* AO1 and *Geitlerinema* sp. BBD_1991 genomes. Genes located in potential prophage regions are coding for known virulence factors and indicate that bacteriophages and foreign genetic material are likely contributors to the virulence of *R. reptotaenium* AO1 and *Geitlerinema* sp. BBD_1991. Maintaining CRISPR-Cas systems is costly, but crucial for protection against a potentially high number of lytic and lysogenic bacteriophages present in the BBD consortium. While a lytic infection could decrease the abundance and biomass of cyanobacteria in the mat, a lysogenic conversion could introduce new genetic material that might change the phenotypic characteristics of the infected cyanobacteria. Under both scenarios, an infection might alter the cyanobacteria’s functional role within the BBD mat: i.e., increase- or decrease its virulence. The detected CRISPR-Cas systems and potential prophage regions are evidence of a close interaction between bacteriophages and their host and highlight viruses as functional members of the BBD microbial consortium and as possible contributors to the virulence of the BBD-associated cyanobacteria *R. reptotaenium* AO1 and *Geitlerinema* sp. BBD_1991.

## Ethics Statement

No ethics approval required for corals.

Sampling permit: Great Barrier Reef Marine Park Authority.

Permit number: G14/.1 to: James Cook University, School of Marine and Tropical Biology, QLD 4811.

## Author Contributions

PB: Designed the study, performed experiments, data analyses, contributed materials, wrote the manuscript, and reviewed the drafts. EW-C, KW, BW, and MvO: Designed the study, contributed materials, wrote the manuscript, and reviewed the drafts.

## Conflict of Interest Statement

The authors declare that the research was conducted in the absence of any commercial or financial relationships that could be construed as a potential conflict of interest.
