# Supplementary material for: CRISPR-Cas Defense System and Potential Prophages in Cyanobacteria Associated with the Coral Black Band Disease
Source: Front Microbiol. 2016 Dec 22;7:2077. doi: 10.3389/fmicb.2016.02077 (PMC5177637; doi:10.3389/fmicb.2016.02077)

CRISPR-Cas in black band disease associated cyanobacteria

**Supplementary material**

**Table S1 Web access links for bioinformatic tools.** Follow the links to access bioinformatic tools and genome data.

| **Tool** | **Web access** |
| --- | --- |
| RAST | http://rast.nmpdr.org/  Access with guest account, username: guest; password: guest.  ID: Roseofilum reptotaenium AO1 (564709.3), Alphaproteobacterium (28211.29), Cytophagaceae sp. (89373.4) |
| NCBI GenBank | www.ncbi.nlm.nih.gov,NCBI GenBank MLAW00000000.  for Roseofilum reptotaenium AO1, Alphaproteobacterium, Cytophagaceae sp. |
| Artemis | www.sanger.ac.uk/science/tools/artemis |
| CG-View | http://stothard.afns.ualberta.ca/cgview_server/ |
| CRISPRfinder, db | http://crispr.u-psud.fr/crispr/ |
| CRISPRtarget | http://brownlabtools.otago.ac.nz/CRISPR_WEB/crispr_analysis.html |
| Bandage | Wick RR, Schultz MB, Zobel J, Holt KE. Bandage: interactive visualization of de novo genome assemblies. Bioinformatics. 2015 Jun 22:btv383. |
| PHAST | http://phast.wishartlab.com/ |
| PHASTER | http://phaster.ca/ |
| VIRsorter | https://de.iplantcollaborative.org/de/ |

**Table S2 Functions of *R. reptotaenium* AO1 and *Geitlerinema* sp. BBD_1991 CRISPR-Cas type associated genes.** CASCADE = CRISPR associated complex for antiviral defence. The gene Cas10d is representative for the CRISPR-Cas type I-D. Functions from Makarova et al., (2011a, b).

| **CRISPR type** | **Cas gene** | **Function** |
| --- | --- | --- |
| I-D | Cas2 | Spacer acquisition, protospacer recognition |
|  | Cas1 | Spacer acquisition, protospacer recognition, also RNAse |
|  | Cas4 | Not sure, might be involved in spacer acquisition |
|  | Cas6 | RAMP family RNAse, for crRNA processing, snaps ssRNA and dsRNA |
|  | Cas5 | RAMP CASCADE subunit, RNA cleavage, might substitute for Cas6 |
|  | Cas7 | RAMP CASCADE subunit, RNA cleavage |
|  | Cas10d | Large CASCADE subunit |
|  | Cas3 helicase | Cuts DNA during interference, promotes strand separation |
|  |  |  |
| **CRISPR type** | **Cas gene** | **Function** |
| I-D | Cas2 | Spacer acquisition, protospacer recognition |
|  | Cas1 | Spacer acquisition, protospacer recognition, also RNAse |
|  | Cas4 | NA, might be involved in spacer acquisition |
|  | Cas6 | RAMP family RNAse, for crRNA processing, snaps ssRNA and dsRNA |
|  | Cas5 | RAMP CASCADE subunit, RNA cleavage, might substitute for Cas6 |
|  | Cas7 | RAMP CASCADE subunit, RNA cleavage |
|  | Cas10d | Large CASCADE subunit |
|  | Cas3 helicase | Cuts DNA during interference, promotes strand separation |
| III-U | Csx3 | Not yet assigned to a specific cas subtype |
| I-MYXAN | Cas4/Cas1 | NA |
|  | Cas5/Cmx5 | NA |
|  | Cas7/Cst2 | NA |
|  | Cmx8 | NA |
|  | Cas3 | NA |
|  | Cas6/Cmx6 | NA |
| III-B | Cas1 | Spacer acquisition, protospacer recognition, also RNAse |
|  | Cas2 | Spacer acquisition, protospacer recognition |
|  | Cmr2 | NA |
|  | Cmr3 | NA |
|  | Cmr4 RAMP | NA |
|  | Cas1 | Spacer acquisition, protospacer recognition, also RNAse |
| Cas genes | Cas1 | Spacer acquisition, protospacer recognition, also RNAse |
| Cas genes | Cas6 | NA |
| Cas genes | Cas1 | Spacer acquisition, protospacer recognition, also RNAse |
| Cas genes | Cas1 | Spacer acquisition, protospacer recognition, also RNAse |

**Table S3 Direct repeat (DR) BLAST similarities.** DR sequences were BLASTed against the CRISPRdb with default parameters and top 3 hits reported in the table.

| *R*. *reptotaenium* AO1  **DR sequence (array #)** | **Organism** | **RefSeq** | **Identity** | **E-value** |
| --- | --- | --- | --- | --- |
| (1)  GTTTCAATCCACAGCAATCTCTATTAGATTTTGAAAC | *Tistrella* *mobilis* KA081020-065 | NC_017958_2 | 0.938 | 3.20e-002 |
|  | *Tistrella* *mobilis* KA081020-065 | NC_017957_2 | 0.938 | 3.20e-002 |
|  | *Tistrella* *mobilis* KA081020-065 | NC_017957_1 | 0.938 | 3.20e-002 |
| (2)  GTTTCAATCCCTCATAGGGATTTATGTTGGTTTCAAC | *Rivularia* sp. PCC 7116 | NC_019678_16 | 0.946 | 7.00e-011 |
|  | *Rivularia* sp. PCC 7116 | NC_019678_4 | 0.919 | 1.00e-008 |
|  | *Rivularia* sp. PCC 7116 | NC_019678_19 | 0.919 | 1.00e-008 |
| (3)  GTTGAAACCCATCTAAATCCCTATGAGGGATTGAAAC | *Cylindrospermum* *stagnale* PCC 7417 | NC_019757_35 | 0.964 | 3.00e-008 |
|  | *Nostoc* sp. PCC 7120 | NC_003272_20 | 0.912 | 3.00e-008 |
|  | *Cyanothece* sp. PCC 8802 | NC_013160_1 | 1.000 | 4.00e-007 |
| (4)  TGTTTCCAACTAATCCGATTTAACCCAATCGGTAGGG | *Cyanothece* sp. PCC 7822 | NC_014533_1 | 0.944 | 7.00e-011 |
|  | *Rivularia* sp. PCC 7116 | NC_019678_24 | 0.917 | 3.00e-008 |
|  | *Cyanothece* sp. PCC 7424 | NC_011738_1 | 0.917 | 3.00e-008 |
| (5)  GTTGAAATCGACCTAAATCCCTATTAGGGATTGAAAC | *Rivularia* sp. PCC 7116 | NC_019678_19 | 0.946 | 7.00e-011 |
|  | *Rivularia* sp. PCC 7116 | NC_019678_31 | 0.921 | 2.00e-010 |
|  | *Cylindrospermum* *stagnale* PCC 7417 | NC_019757_35 | 1.000 | 1.00e-008 |
| (6) GTTTCAATCCACAGCAATCTCTATTAGATTTTGAAAC | *Tistrella* *mobilis* KA081020-065 | NC_017958_2 | 0.938 | 3.20e-002 |
|  | *Tistrella* *mobilis* KA081020-065 | NC_017957_2 | 0.938 | 3.20e-002 |
|  | *Tistrella* *mobilis* KA081020-065 | NC_017957_1 | 0.938 | 3.20e-002 |
| (7)  GTTGAAATGAACATAAATCCCTATTAGGGATTGAAAC | *Rivularia* sp. PCC 7116 | NC_019678_16 | 0.947 | 2.00e-012 |
|  | *Rivularia* sp. PCC 7116 | NC_019678_19 | 0.946 | 7.00e-011 |
|  | *Calothrix* sp. PCC 7507 | NC_019682_29 | 0.919 | 1.00e-008 |
|  |  |  |  |  |
| *Geitlerinema* sp. BBD_1991  **DR sequence (array #)** | **Organism** | **RefSeq** | **Identity** | **E-value** |
| (1)  GTTTCAATCCCTAGAAGGGATTTTATCGGATTTAGAG | *Halothece* sp. PCC 7418 | NC_019779_1 | 0.956 | 5.00e-06 |
|  | *Calothrix* sp. PCC 6303 | NC_019751_6 | 0.923 | 5.00e-06 |
|  | *Nostoc* sp. PCC 7120 | NC_003272_3 | 0.923 | 5.00e-06 |
| (2)  GTCTAAATCCGATAAAATCCCTTTTAGGGATTGAAAC | *Crinalium* *epipsammum* PCC 9333 | NC_019753_5 | 0.961 | 1.00e-07 |
|  | *Crinalium* *epipsammum* PCC 9333 | NC_019753_18 | 1.0 | 1.00e-07 |
|  | *Crinalium* *epipsammum* PCC 9333 | NC_019753_17 | 0.961 | 1.00e-07 |
| (3)  GTTTCAATCCCTAAAAGGGATTTTATCGGATTTAGAC | *Crinalium* *epipsammum* PCC 9333 | NC_019753_5 | 0.961 | 1.00e-07 |
|  | *Crinalium* *epipsammum* PCC 9333 | NC_019753_18 | 1.0 | 1.00e-07 |
|  | *Crinalium* *epipsammum* PCC 9333 | NC_019753_17 | 0.961 | 1.00e-07 |
| (4)  GTTTCAATCCCTAAAAGGGATTTTATCGGATTTAGAC | *Crinalium* *epipsammum* PCC 9333 | NC_019753_5 | 0.961 | 1.00e-07 |
|  | *Crinalium* *epipsammum* PCC 9333 | NC_019753_18 | 1.0 | 1.00e-07 |
|  | *Crinalium* *epipsammum* PCC 9333 | NC_019753_17 | 0.961 | 1.00e-07 |
| (5)  GTGCTCAACGCCTAACGGCGATCGAAGGAAATTCAC | *Leptospira* *interrogans* L1-130 | NC_005823_1 | 0.956 | 5.00e-06 |
|  | *Stanieria* *cyanosphaera* PCC 7437 | NC_019748_5 | 0.916 | 2.00e-04 |
|  | *Synechococcus* sp. PCC 6312 | NC_019680_1 | 0.892 | 2.00e-04 |
| (6)  GTTTCCATTCATTCCACTTCTCTAAAAAGAAGCGTC | *Cylindrospermum* *stagnale* PCC 7417 | NC_019757_28 | 0.878 | 2.00e-05 |
|  | *Stanieria* *cyanosphaera* PCC 7437 | NC_019748_1 | 0.818 | 0.009 |
|  | - | - | - | - |
| (7)  CTGACAGCTTCTTTTGAAGCGGAATGAATGGAAAC | *Trichodesmium* *erythraeum* IMS101 | NC_008312_20 | 0.952 | 2.00e-04 |
|  | - | - | - | - |
|  | - | - | - | - |
| (8)  GTTTCCGTCCCCTTGCGGGAAAAAG | *Crinalium* *epipsammum* PCC 9333 | NC_019753_6 | 0.958 | 8.00e-07 |
|  | *Synechococcus* sp. JA-3-3Ab | NC_007775_2 | 0.958 | 8.00e-07 |
|  | *Crinalium* *epipsammum* PCC 9333 | NC_019753_3 | 0.956 | 3.00e-06 |
| (9)  CATCCCCCGAAGGGGAAGTCGATCGAAAAG | *Synechococcus* sp. JA-3-3Ab | NC_007775_10 | 1.0 | 0.089 |
|  | - | - | - | - |
|  | - | - | - | - |
| (10)  ATTTCCATTCATTCCGCTTCAAAAGAAGCTGTCAG | *Trichodesmium* *erythraeum* IMS101 | NC_008312_20 | 0.95 | 7.00e-04 |
|  | - | - | - | - |
|  | - | - | - | - |
| (11)  ATTTCCATTCATTCCGCTTCAAAAGAAG  CTGTCAG | *Trichodesmium* *erythraeum* IMS101 | NC_008312_20 | 0.95 | 7.00e-04 |
|  | - | - | - | - |
|  | - | - | - | - |

**Table S4 Spacer matches to contig 93.** A# = CRISPR array number. S# = spacer number out of total respective array. %Ident = percent identity of query to spacer target in BLASTn. M/G = number of mismatches or gaps in alignment of protospacer and spacer. E-value = BLASTn score of query match.

| A# | S# | Sequence | Target | Gene target | %Ident | M/G | E-value |
| --- | --- | --- | --- | --- | --- | --- | --- |
| 2 | 4/14 | GATCGGGCCCCCATCTGGGAATGCCCCGAGTTT | Contig_93 | hypothetical protein | 100 | 0 | 2e-011 |
| 2 | 7/14 | CCAAAGGGTCCCCACTTACTGGATGTCTTGAAATATCCA | Contig_93 | hypothetical protein | 97.44 | 1 | 5e-013 |
| 3 | 12/12 | CAAACCTATTTTTACTATTTCCGCAATTTATGGCAA | Contig_93 | hypothetical protein | 100 | 0 | 5e-013 |
| 5 | 6/11 | GAAACATTCAGAATCAAATGGGAGGTTGATTGGGA | Contig_93 | hypothetical protein | 100 | 0 | 2e-012 |
| 5 | 7/11 | CCGGCACCAGATTTTCCGGATGAATTCCTAGAAG | Contig_93 | hypothetical protein | 100 | 0 | 5e-012 |
| 5 | 9/11 | GGGAAATTCTCCTTTCGGAGCGCTCGGTGAAATTCTTG | Contig_93 | hypothetical protein | 100 | 0 | 4e-014 |
| 6 | 11/27 | CTGGGTTAAGAAGAGGATTCCATTCGCTATAATCAAAATCAT | Contig_93 | hypothetical protein | 97.3 | 1 | 3e-011 |
| 6 | 14/27 | GCATAACAAAATACGATCGGGAAACATTGAAGAGCCCAAT | Contig_93 | DnaB domain-containing helicase,  VIRsorter: Phage_cluster _71 _PFAM-AAA_25 | 100 | 0 | 3e-015 |

**Table S5 Short contigs of *R*. *reptotaenium* AO1 that are connected to self-target region contig_93.** BLASTx matches (nr database) are indicated below the respective sequences.

**>Contig_184_length_979_cov_476.981_ID_367**

ATCGCCTACGACCCCAACCGAGAAGCGCGGGCCGAACAACTGCGGCTGAAGAACCAACCCACCATTCGGG

AACTGTGGGAGAAGTACAAAATCAAGCAGTTAAAACTGACCTCCAAAACCACCCAGAAAAGCGTTTGGGT

AGAGATTGACCGGGCACTGGATGCCTTATCCAGTAATGCCTTGAAGCTTGAGAGTCTGGATAATCTAGGC

GATGAGTATATGAAGCTCTACGCGATCGCCACCTGCCACCGTCATTTCGAGTCACTGCAACCAGCCATCC

GGTTGCACCTTCCCAACATTAAACTCAAGCCCCAACTTCCCAAGAGGGTAAAACGCCCTATCGAATGGTT

CCCCCCGGATGAAGTCAAAGTCATATTACAAGCGTTCAAAACCGACCGTTTTTCCTCTCCCTTTGCCCCA

GTTCCCCACAGCTACTACT ACCCCTATGTCTGTTTCCTCGCTCATACCGGGTGCAGACCCGAAGAAGCGA

TCGCCCTGCTGTGGTCAGATTTGTTCTGGCTGCGCGATCGGGCCGGTTGCGAAGCCTCCATCACCAAAGT

CTTCTCCAAAAAGGTCCTCAAACCCTACACCAAGAACCACCTCATCCGAAACATCCCCATCTCCCTAGCC

CTTCAAGACATATTAGAGGAGCGAAAGAAGCGATCGGGCTTAGTCTTCCCCTCTCCCCAAAAAAAGCACA

TCGATCAGAGCAACTTCTCCAGTCGCGTCTGGAACTGCGTCCTCCACTCCCTAGTTGCAGAGGGAGAAAT

CCGCAAGCGACTACGCCCCTACTGCCTACGTCACTCCTTCGTCACCAATATCCACCATGAGCATGGAGTT

CCCTTCCCCACCATTGCCCACCTGATTGGCGACAAAATTGAAACCGTCATCCGGTTTTACTCAGGAACCA

AACCCCTTACCACCCAAACCTTCCCCAACCTCTATTAAAACCCCTTCAAACTGGTACGATTTTGGATCG

BLASTx M.score T.score Q.cover E-value Ident Accession

phage integrase family protein [Stanieria sp. NIES-3757] 169 169 88% 4e-46 39% BAU63200.1

**>Contig_156_length_1881_cov_520.087_ID_311**

ATTGCCCACCTGATTGGCGACAAAATTGAAACCGTCATCCGGTTTTACTCAGGAACCAAACCCCTTACCA

CCCAAACCTTCCCCAACCTCTATTAAAACCCCTTCAAACTGGTACGATTTTGGATCGGAATTTCCGGCTC

CAATTGCCCACCTGGTAAAGGCTTGCCAGTTTGAGTAAGCGAAAAAAACTGCCCCAAAGTGGTACGATTT

TGGTACAGGGGTATTTTTAGAGAAAAATCTCAAACGGCTGAAACCCTTGCTGTGACTGGGATGGACGTAA

CGGGACTCGAACCTGTGAACCTTTAAGGGGTTGATGTCATTCCAAAGGCTAAAAGCCTGACCATGCATAC

ATTCAACGCATTAAACCTTAGAAATGTGTTGAAAATGTGTTGAATATGGACACCTACATAAAACAAGCAT

GGAGCCTGGTGAATGAGTACTTCTACTCTAACTCCATTGACATCTCCAAGCAGGTAGACCATGAGCTAGT

GAGGGCATACCTCAAAGCTTGCCAGAAGAGTACCCCCAAGGGTATTCGTATTGTCAGTAGCGGTAACCGA

CTGTATCTCCGATTCAAAACTACGACTAAGCCAGCAACGGTTAACAATTCCTGTAATGAGGACTTCACTC

GCGATGGTTGTATTAACGCTCTAGCTAAAGCCTTAGCTGTCTTTAACAAACTCAAGGAGACTGAAGCAGA

GTCTGAATTCTGGTCGTGGTATGAGTCAGAAATTAAAGGCATTGTACTCCTCAAGAATGACATTATTACC

ATTGGTCAAGCTATTGAGACTGTCAAATCAAACTACCTACTAGGTCGCGACAAGTGTGACCGAAATAGGA

ATGACGAAAAGCTGACGACTAACAGCTTGAGTATATACAATAAGACTTATGGCGGACATTACAAGAAACT

CAATCCATGTCTCAGACTTACCGGGGAAAACATAATCTCTGAAATCATGAGGAACTGGGGTCAATTAATT

ATTTCTACGACTGGCTCTCAAACCTTGTGTTCCAAAGGCTTTAAAAATGCCTATACAGCTTGCTGTAAGC

TTCTCCGGGATACAAAACTCTCATCAGAGCTGGACAAAGTTACCAGCTATTTTGAGAAATTGAAGGTCAC

TAGGAAAACAAAAATGCAAGCCATTGACCTTGAAGCCTTCCTAGACTTTAGGGCTAGAGTATTGGGGCTA

AATGGTTATGAGCTGACTAAAGCTCAATGGCGTAACATTGAGTCACGCAAATCATGGATGAAAGCTATTT

GCATTAATTTAATTTACGGCTTTCGTGCCTCTGAGTTCAAAGCAATTTTGAATTTTGATAAAGCAATTAC

TTTAGATGGTTACACCTTTTATGCACTTGATGACCCCAGTAACAACGAAAATATTGTAGTTATTGACGAA

GGCTTTTGGATAACTGATACTAGCGGAGAGTGTCATTACATCACAATTAAAACAGGCAAACGTATTGCAC

GTCCGATGATTCATCCCGATTACCCTAACTTGGTTGAATTATTGGGAATAAAAGACCCAAGAGTCAAGAT

ACCTGAATGTATCCCTAAAGCCAGTAGCAACCCGGACACGATTAAAGATATTTATACCCGTCAAATGGGG

CAGAGATTGGCTGATTACATTTCCCAAGTAGGAGGTCAAGGATTCACTCAAACTCATGCCCTACGCCACT

TAGCAAATTACCATGGCAAACTTGCCGGTTTAACGCGCGACCAACGTGCTTTGTCACTGGGACACTCTCA

AACAATGAATGACAAATACGACAAACATCAAACTACCAGGAATCAGGTGAACCTTCTGATGGCTGACATC

TCTGAGAAATCAGAAATCCAAAGGCTAAAAGATGAGCTTTCCCAAGCTCAGGAGACCATTA

BLASTx M.score T.score Q.cover E-value Ident Accession

hypothetical protein [Lyngbya aestuarii] 323 323 56% 6e-102 46% WP_023065230.1

**>Contig_152_length_2049_cov_1399.3_ID_303**

ATGAATGACAAATACGACAAACATCAAACTACCAGGAATCAGGTGAACCTTCTGATGGCTGACATCTCTG

AGAAATCAGAAATCCAAAGGCTAAAAGATGAGCTTTCCCAAGCTCAGGAGACCATTAAATCCCTAGAGGA

AACCATTCTCTTCCTGAAGAAGGAGAACGCCAGGCTGAATGAACTTTTAGGAGGTAACGACGACCTTCCC

AGAATTGATTAGAATGCAGGCGAATCCCCTTCTACTTGGAAGGGGTTAATTTACCTTAAATTTTTAAGGT

AAATATCTCATTACCTCCCTAACTTGGTGTTCGTCAAAACCCCATTCCTCCATCTGCCGACACAGTGTAG

AGAGTCGTCTAACGCTGAGTTTAAGAACACGAGTATTACCCTGATTATCTAAGTTACTAAAAACGACAGC

GTATGAATTAGAATTTTTAAGCTTCTTAATATTCAGAGTGCATTTTCCCCCCTCAACTACTCCACGCCAT

TCTGTTGAATTTTTATCTGAATTCCTTTTCTTAATCAATTCTCTGACAGCATCCTTCACTAGCCATGATT

CATTGATGGCTGTATACACTGCTAAATCGATACATTCCTCAGATTCTATACAATCCCCCCATTGATTGAT

TGTTGATGGGAATAAATCAATTATTCTCAACCACTCACTAACTTGCTCTAGCTTGCAGTCTAATTTTGAT

ATTCTGTCACACAACGCGTAAATCGTATCAGATGGTTTAGAGTAACGCTCTAATACGACCTCCCTAAAGT

CAATTGATAAAGCCTCTGCAATTTGGTCAATCAAATCAGTCTTGAGCCAACCACCGTCTACTAACGTAAT

AGTGGTATCCATTTTTTCGTTTAAAATCTCAGCCATTGCAACCTCATTCTTTCGTATAGAGAGTGTAATT

TACCTTAAAAATTTAAGGTAAAATTGCCATCTAAACCTCTATTCTACCGTTAGGTCTTTAATCTTAGGTC

TCTCCTGCCGTGTCCGGTTAAACGGCAGTGCATTATCTCCACTCGTTCGGATAGTAATTTGGCTACCATC

AGGAGACCTATAAATACCATCTGGCGGCAGATATCCCTCCTCAGTAAACTCTAGAAACTTCCGCCAATCT

TTCTGCTTGTACCTATCAGTATCTTCTAGACTTTCCGGCTCCTCTGTAATACCTTTAGCTTTCTTTACAG

CCCTCGTCCCCTGATTAACTTTGGTCTCGAAATCTTTAAGATACTTATTCAGATTCTCCTCCAAGTCTTC

TGAACCCCCTACAGACACAGTGAAACTATGTTTAATAATAGATATCGCCTGCTTTACATCAACCATCATA

TCCATTAAGTCTTCCGTCGAAGATTCTAATTCATAGGCTTTTACGGGGTTGGTGGATGGCTCCACAAATT

TATCTAATTGTTTGTCAGCGTAATCGTCAGTCGGTAAAGTATCTTTACCCATCTCCTCCTCCCATCCAGG

AATACTAAAAGCTGTAAAACCACCCCCACCAATTAAATTAGCTATATCTGGAGAAACTAAACCAGGGATA

GTAAAAGATGCCCTCAACTTTTTAGTGACTTGCTTGTATTTGAACCCTAGCCAATCGATTACAACTTCTA

TTCTATTTCTAGCCTTATATGCCTCCTCTCTGGCTTGGTAAGCTTCCAATACAGCCCTCATCCCTAGAGC

CTTAATCGTTCTTTCATCATCCTGCAAGTTCATGTTCAGCTTGATTATTTCAGTTATTGCGTCTTCCAAG

TTGTGTACAGTGACATTAACCGGTAAATTTTCGCCCGATTGTTCTGCTGCCTCTCTAATCCTTCTAGGGA

CTTTAATCGTGAAGGGAAACTGACCAAAAAAACCATCCAGGCAATCCATAAAATGCCAGATTAGCTCAGG

CTGAGAGCGAATAATCTTATTCTCCATAAAAGCGTTGAATCCAGTAGGCACAATCCCCTCTATACCATCG

TTGGAAATAAATGGGTTTCCAGGGACTCTAATCGCTGTGTTATTCGCCCCAGTCCACTGTTTAATCATCC

TCATATCACGCTGAATTTC

BLASTx M.score T.score Q.cover E-value Ident Accession

no match

**>Contig_165_length_1517_cov_432.414_ID_329**

CGTTGAATCCAGTAGGCACAATCCCCTCTATACCATCGTTGGAAATAAATGGGTTTCCAGGGACTCTAAT

CGCTGTGTTATTCGCCCCAGTCCACTGTTTAATCATCCTCATATCACGCTGAATTTCGTTAAGTTTTCTG

ATTACTGTTGGGCAGCAATTCCTCATATCATCCTCCTCTCCTCTTGGATTTCTTTTCAGTTTATGTAAGG

GGACAATTCCTTTCCTTGGGGGGAATTGAACAAGCCTATAAGGCACTCTAATTATAATTGATGCGCTGTC

AACATCTGTCCCCCAATCAGTAAATACTTCACCAGTTACGCTATTGATAGCCGTCCCCGGAGTCCCGAAA

GCGTTAACCCTATATTTACCATTTGGTCGTGGTTCTTTAGTTGGAGTTTTTCCTGCCCCATCCGTTGTGG

CACGCTCTCTATAATTAAGGAATTGCTCCATCGAGCTAGGTAGTCCCCATCTCGGACTGACTGGAGGGAC

TTCGTAGGGGTGACTCCACTCCATTCTTCCGTTTCGATATGCAGAATTATTGTAAACGTATATCTTTTCC

CCCAATCCATTTGTGTAACTTCCAATTACTCCCGAAGACCCAACTGGAAGAAGAAAACCTAATCTACTAC

CGTCTGGGTTTGAGGTTGGTCTGCCCGCTTCAAAATCTACGCCTCTAGCCTCTATTATGCCGTCAATGAT

TTCCGGGGTAATTCCTCCCAAAGATTCTGTATTTCCTGGAGGATAACTAATTGTTATTGTGTCTAAATTT

ATCGTTTCCTGAGTAACTTTATTTTCCTCTCTTATTTGTTGCTCTATGATATCCGGTACTTCGCTTGGAC

TGGTGTTAGAAATAATATCATATATTTCTCCTAGTGCTTGATATAAAGTGTAAACATCGTGTGCCCATAA

AGCTACGTTTAAAGCCGCTCCTGCTGGTCCGGTTGCGAGTGTAGTCAAGGCTAATCCTCGTTTTCCAGCC

CACTTTAGAAATTCTTTGACAATTCTTCCCCCTCCAATCTTACTGCCGCGCCTTAATAATCTAGCCGCTG

CACCAGTGAACCCACCTGAAGGCGCGGCATCTCTGAGTTTATTTCTAGGATCTAATAATTGTTTAGTTTT

TTCGGGGTCTCTTAACCATCGTTCTATAGGTTGTGCTGCCAATTCGCTATCGATTCTTCGACTAGGAGAT

AGAACTCTTGTAGCCCCTTCTCCCATGATGGCACTAGAGGTATTAGGCACTGGCGCTCTTAAGGATGTTG

ATTGATTGGGATTATATGGAGAAATACTTCCACCTCTTCCCGCTTTAATTCCATCAAAAGGATTGGGATT

TCTCTTGGGCATGATAACCTCATCGATTTGTACGGGTATGGCTACCGATGAGGTTCGTTGTGGTATTCCC

ATGACAGTTTTACCTTAAATATTTACCTTAAAATGGTCTGGGCATGGTCTCAATTGAATCGAAAAAGATT

TGGTCGAGCCACTCTTTTATCATGTTATCCATGGTTGGGTTATCTTC

BLASTx M.score T.score Q.cover E-value Ident Accession

no match

**>Contig_166_length_1508_cov_816.137_ID_331**

CGTTGAATCCAGTAGGCACAATCCCCTCTATACCATCGTTGGAAATAAATGGGTTTCCAGGGACTCTAAT

CGCTGTGTTATTCGCCCCAGTCCACTGTTTAATCATCCTCATATCACGCTGAATTTCATTTAATTTTCTA

AGTACCTTTGGGCAGCATTGCATGTTATCATCCTCCTCTCTATTTCTTTTTCTTTTTGCCTTTGGCTTGG

GTACAATTCCTGTCTTTGGCGGGGAATGTAGCACTATGTGAGGTATGCGGTCAACTCTCGCATATCCAGT

AACATCAACACCCGTACCGCCTGATATTGCATCGACTGTAATTGTTTCTCCATTTATCTCTATTTCAGTG

TTACGCCCTAACATCGAAGCAGGAGCTGTTACCGAACCCAGGGTATAAAGTCCATTTGCATCGGGTTCAC

CCTTAGTAAATTCTCCCCAATCTTTAAACTTATCTAATCTTTTTATATATTGCTCCATTGTGGAAACTAT

GTCCCGAAAGTTTAATGAACGCGGTTTTATTGTATCAAAGGGATAGCTCCATTTAATCTTTAATCCCGTA

CCGTAGGAAGTAAGTCCAACGCTACTAATAACTTCCCCGAACTCACCGCGAAGCCCGCCTAAAACAGCGA

CGTTATTAAAAGGAGTACGTCCTATAAAGGCACTATGTCCAATTGCCTTGACGTAACCTGATTTACTTCC

ATCACTTCTAGGGGTTGGGATATTTTCTATTTGCTCAATAGCTCCTGCCCCTTCTTGGATTCCTTCGACT

ATACCATCACCATATTCTTGTATGATTTCTTGAATAGATTCTGTGGAAATATTGCCCGCCGTTTCAATAG

CGCCTTCCAACCCAGAATACTTTTCTAGCAATTCTTTTCTCGCTCCTTGTTCCACGATATCTGGAACCTC

ACTTGGGCTAGTGTTCCCCATATTATCGAGAATATTCTCTAACGCTCTATATCCTTGGACGATAGCTATA

CCGCCAAGAACACCAGCGCCGATTAGCAGTCCCCCTTTGACTGTGATGGCACCACCAACAACCAATTTAG

TGCCCTTAATTGCTCCTCCAACAACCTTTTTCCCCGCAGCAGAGAATCCAGCCTTGCCGCCACCAATTCC

CCCAGCGAGAACCTTTGCGCCCTTACCTTGAAGGGCGCGTTGAGTTTTAGTAGGGTCTTTTAGCCAGTTT

TCTATTGGTTGTTTTGACAGATTCCAGTCTGATATTAGGCTACCTGCCGACGTTGGAAGCTTAACTGATG

CTCCCTTCAAAGTCCCTCCTGTCGGAAAGATTGCTGACCCTGTTGTAGGGGGCGATATTACCGTTGTTGG

CATCTTAGCCGCAACCTGTTCAGGCAGGTTAGACGGCATTGATATTGTACTTGGTATTCCCATGACAGTT

TTACCTTAAATATTTACCTTAAAATGGTCTGGGCATGGTCTCAATTGAATCGAAAAAGATTTGGTCGAGC

CACTCTTTTATCATGTTATCCATGGTTGGGTTATCTTC

BLASTx M.score T.score Q.cover E-value Ident Accession

no match

**>Contig_138_length_3886_cov_1381.31_ID_275**

TGGTATTCCCATGACAGTTTTACCTTAAATATTTACCTTAAAATGGTCTGGGCATGGTCTCAATTGAATC

GAAAAAGATTTGGTCGAGCCACTCTTTTATCATGTTATCCATGGTTGGGTTATCTTCTCGGTAGTATAAC

TCAAATCTTTTGCTCATCTTCCTAGAAGACTTCTCTCTGTTTGGGTCATTTAATCGGTATCTACCTCGTC

TATCTTGACCCGTGCGAACAATCCAAAAGCGAATAGGGTAAACCGTCATCTCTTCTGGAAACACTCCCGA

TTGAATGTTGTCAAGGTTTTTGACTTTTTCGGGTAACGTCATATTCTTGATCTGGTCATCAGAAACTAAG

TTAAATAAATCTCGAACAAATCGCTCAGATTTATTTTTGTCATCTGTTGAAAAACAGGTATTAGTCCCGC

TTCGCAGGTCAACCCAACACTTATATTTACCTAGCCGGATGCCACTACTTCCACCGCAAGCCGCCTTTAA

TTGCTTCCACGTCCAGCCAGATCGTTTGACCATAGGAATAGTCATCGTAACCGTTTTATATGGTTCACCG

GCTCTCCTTAATGGGGGTCTAGGCTGGTTGCACCATAAAATTTTCATACTGATTGTATGAGCCGCTTTGA

GTTTGTATGCTGGGGTACTTCCTTCAATTATTTCCCCTACATCCTTCTCATACATCATCTGGTAATTAGC

TAAGAAGTTTGTGATGTCATTTCTACCTATGTTTTCTCGCGTAGAAAAGTAATAGGGAGTTCCTTCTTCT

CTATTGGGAATTACTTCAATGAATTTTTGCTCACCCAATACGATATCGGGTTCCTTGACTCTATTCAAAG

CCATCTGGCTATCGATAGCATCCGCTACTACCATGAATCCTTCTTTGCAGGACTGCCAAAGCTCTTCTGA

AAATTCTTCTACAAATTCTCTAACATTTGGATTTTCAATATCTTCTATTCTGTCTCTCTGCCATTGTGCT

AGAGACAGGGTTTTACTCCCTTCTTCTCCCCATTTATCAAGAGCTTCCTGTCCGAACACCCTCTGGGCTA

TTCCATTCTTTTTAATCGCTTTTCTAGCATTTTGAAAAGCAAAATAGAATGCATGTTCTTGTCCTAATTG

ATAAATAGCCCTACCAGTTTGCACCCATTCCTGCTTGATTTCTTCGTATATCTCTTCCGAAACTTCATCC

CTAATTGCTAGTGCCATGGATGGGTCTATCTTAAAGGTCAAGGTATTGGCTATTTGACCGCACAATACCT

GCCCAACCGTTACTCCTAGCGCACCGCCTAACATACCAGCCCCTTGCTGGATAAGTTGCTGCCTCTGCAT

GTCAATTTGGGTATCTGTAGTGTTCCAATCAAAGTTCCACAAGAATTCAGCAGACTGCAATAATCCGCCA

ACGATTGTAGATACTCCAAGAAACTTTAATACATTCCCGGCGACTGCTCGAAGTAACCCGGCAATCCCCT

TCTCCAAAAAGGATTGAGCCAGACCCGTTCCTACACCAATAAGTTCAGCTTGCTTTTGTCGGTAAAAATC

TCTTAGTTCGTTCATGAGCCGTGCCCATAAACTTTTAGTAAAAACCTTTATACCGCCACTGCCGCCGCCT

CTATTTCCTCTTAATTGTGGTGGTAGCGCTAAAGAGCCAGGCATGACAAAAATCCTTTACTAATCTACTA

AAGAGTACCATAACTTAATAGTATATATAATTTCTTCCTTCGGAATTCTACCCATCCATCCTTTATAAAT

TAATAAACTACTTTCCTCTAAATCATTTTTGATAAAGACGGGTATTCGTGGCGGTCTAGTTATCGTTTTA

TTTATTGTGTTGGGAATGGTTTCTACCCAACGAGTAAAAATGATTCCATTATTGAATGTCTTCAAGCGAA

GCGCTAACCATAATCGACTTCTTACCAGCTTTGGGTCAGCNNNNNNNNNNNNNNNNNNNNNNNNNNNNNN

NNNNNNNNNNNNNNNNNNNNNNNNNNNNNNNNNNNNNNNNNNNNNNNNNNNNNNNNNNNNNNNNNNNNNN

AGGTTCACTAAGAATTTCAATAGCATCATCGTCTACCAAAATTTTCAGTCCATCTATACCATTGGGCATA

ACTTCCACTGGAAATCGTGCAGATACTTGATAGATGTCTTCGTTGCCCGATTGAGTGAGCCTGCCTTGGA

CGGTTGTCCCCTCTTTCAAAACCAATCGCCCTTCCTTTCTGGCAACTTCAATGTCGCCAAATAAATTAAC

GATATCAAGTGCTTGTCCCATGTGTCCTTCTCCTGGTCGCTACATCTCAGTTATAGCGCGACTTTTCGCT

ATTTTCGTGATAAAATGGACTATCAGAACCCTATAGGAGTGTGATAGGACAGTATGAGTGTAATCGTCAT

TCAGAAAAAGGTAATTTGCGATAGTACAGAGCGGCAAATTATCCTAGAACCTCAAGAGCAAGACATACTT

TTTGAAGAGGACTTGGTTTTGCTTTATCGATTCCACTATGACGCTAACGATGGTGGCATTAAGATAACGA

CTCCCTTCGTAGAGCAAAGACCAGAGGATACAGTGGAGTGGGCAGCAGTAAAGGAATATTACAATTCTTG

GGCAATACCCCATATACTCCTTTATTTGTTCGTTTTCGGGCAAGAAGTAGACGATGACGCACCTCGACAG

ACTCTCGCGTTACCTAATTATGGTGTGCCTTTTCCGTTACCGCTCAACAATCCTATTCGCCTAAAAGCAG

GGCAAGGATTAGCCATTGAGTTAGTCAATAATGGGTATGATTTTCCTGATAGCAATAGACTTGATAATTT

CATTACTATTTATGGGGATGCTTGGCTGGGTAGGAATGAATTTAATGCACAAACGGTTAACGTGGATGCT

ACTAGCCAGACAACGGTTAACGCAGCAGTGCAAGAAGGAACACCACCAGAAATAGACCTTAGTCAATTAA

GGAAAGTTGGCATGGGTGATATCAGTTCGATTAAAGTTAAATTCAGAATGCCTTCTGGAAGTACCGGAGT

GGCTCTTTATGAACCATCGCCAACCATGAATAATGCAATTCTTTTTGATGATGATGAAGTGGGTTTATGG

CAGCCAGTCGCCAATAACGTGCGAGGAAATAATTCAGATTTAGATATCTTTACAACCCTTAGAAGTTGCG

GTCACGTTATCGAGGTTGAGTTTAATCAAACATTAACGATTGGTCAAATAAATATTGTTACTCCTGATTT

TCCAGATAAAGCCATGGGCAATATTAGGGCAACAGAAGATTATAGAGGATGGAAGCAAATACATACCGGT

TATTTGTCGGGAAATATTTCCCTACCTAAGCCAGAAAGGGCTAGAAAAATAGAAATAGTAGCTCCCTTAG

AAGATAATTTAGTTGATGGAGTTTTTAAGATTTATGAAATAAATCTATGGGTAGCAGAAGAAATATTAGA

GGTGAATAATGACCCGCTAATTATTCAACAGGCAACGTTTAGATTGAACCCAGAGAAGGTAAACACTTCA

TCTTTAGAAACTTATGAATCATTTACTCTCGCAGATGGCTCTACAGCGCCTTTGGTAAAAACAGAGGATA

ATCGAAAATATATTGAATGGAGGTTTACAGGAAATAATGATAAGAAACAGCTTGTTTCTTCTGAGCAATT

TGTAGTTAAGTCGTTATTAATAGAGTTACGCTCTCCTACTACCTCTTGGGAGGTAGATGGAAGCTTTGAT

AACGCTAATGGAGGTATGATAACTGGATTGAATCCACAAAGTTCTCCTTTATTCACTGCCCTCGGTGGCA

CTTCTTCAATAAATTGGTATACAGCCTACAAGAAGATATCAAGAAATAACATAACGTTAACAGGAGACAA

TGTCAGAGGAGTTGATTTATCTCCAATTGATAATTG

BLASTx M.score T.score Q.cover E-value Ident Accession

no match

**>Contig_151_length_2086_cov_936.202_ID_301**

TATTCACTGCCCTCGGTGGCACTTCTTCAATAAATTGGTATACAGCCTACAAGAAGATATCAAGAAATAA

CATAACGTTAACAGGAGACAATGTCAGAGGAGTTGATTTATCTCCAATTGATAATTGGTTCTGGGTATAT

ATCGAACTAAACGAAAGCTTTACCGACCAACTTATGTTAGGGTTGGGGTCTTATTCCTGGCATAGGGTTG

ATGTGGATGTGCGGCAAATATTAACGTATGATTCCTCACTTTCTGAGAGGAATATAAATGATGTAATAGC

TTATTTCGGTACTTCCCCATCGCCAACCCCTACTCCAGTACCTGTTCCCACGCCAACACCATCAGAGCCA

TCGGCGTACTTGGATAGCAACAACATCATTCTTAAGCCAGACACTTTAGCTGATGGTAATTTATCGAATT

GGGGCGGATATGTTCAACGTGGTTCTACGCAGCCAACAGTGACTAGCCATGGCAATTATAAGCTGGTCAG

TTTTTCCGGTTACTCCGAATTGGCGAAATCTCTTTCTAGTAAAGCAAATTTTTATGGCGTAACCTGCATA

ATAAAATCGCCCAATCCCTCTTGGAATAATTGGGGAAATGTTTGGTCTCATAATGTTCCCAATCATTCCT

GGACATTTGCACTTGATAAAGACGGGACAGCCATCGGGAACAATCCGCCTTTATCTCGTGTACGAGTAAA

TAAATCAGAGATTAATCTAATTTCCAACTGGGAACTGGGAACTATAACTAATCTCTTTTGCCTGACGATT

TTCTGGGATGCTTCAATGATTACCCATTCATCTGGGGATTTAGTTATTCACAAGAGCAGTATCTCAGATT

GGCAATGTGAGTATTCATTAGGGGATGTTTTAGTGTGGGGAAATATGCCATCTGATGCCGATTTAATTGG

CGTAGAGGATTGGATGATGGATAAATATTCAATTCTTTGACAGTACAATAAGGAAGAAGAACTAAACAGA

AGGAGAAGCAAGGAAGATGGACGAATTGTTACGAGGAATAACGTTGAGTGAGACTCTAGATTTCCTGGTT

TTATTGGGGGAGTTTTTTCTCGCTGTCATTATGAAGAAATTGAATGACAAATTAGAGAGGATGACTAGCA

AAGAGGGTGTCCAGATGGATGATGTGCTAAGTAAGTTGAACAAGATACAGCTTTGGGTGGATAGTAATTA

GTAGGTCTACCATGAACTTTTACCTTAAAAATTTAAGGTAAATTAACTTTCTCCTCATCGTCTTCTTGAT

GGGGTGAAGAATTTACCTTAAATTTTTACCTTAAATATCGCACTACACCCTCATCGAAGCATGTAGACGA

TGGCTGTCAATATCACCACAGAAGGTATCAACACGTTGAACACATCGATAACATCACGTGCTTTCAGCTA

CTTCTCAGCTTTCCTGAGGTATCTCTGTATTCTATTCATAGCTCTTAAATAGGTCGCAATACTCATCGAA

GGTGATATCAAGGATTCTGCAAAGCGTCTGAATCTGTTTGGGTTCTAATCTTGGAGGTTTATTGCTAGAT

ACACACCTTCTATATGTTGTCGCACTCATTTTGCATTTTTTTACAAAATCCTCTTGAATCAAATCCGTTC

TTTCTTTACGCAATCGCTCCAGAGGGTAAATATCATCAATTTCAACTGGTGCAAACAGTAGCTTTCCTTT

TTCTGTTCGTTGATTCATAAGTCAATTATAGACAAAAATCTATTGTATTTATAAGATTACAATGCTGTCA

ACCAAAACGACCGGTGCTACACTAAGGACATCGGAGCCAAGCACTCCTGGTCTAGAGCCTCTAAGCACTA

CTATCGCTTAACTGAAGGCAATCACCAAGTGTTGACAACCGATACTATCTTTTTGGGGTAAAACCTTTTC

ACGTTCTGTTATGCACCATTTTCTGCGTGTATATGCAGAGATGTCGTAACAGTCAAAAAAGGCTTACTCT

CCATTGTACCTTGACAATTTGGTTCACCTGTTGACCCTTCTTTCACTTACTCAATCTGAAGAAAGGAGTC

AACAAATGGTTAAACAACAACGCATTCCTGAAGTAGCAGGAGGGTTTAACGTTTTG

BLASTx M.score T.score Q.cover E-value Ident Accession

transcriptional regulator [Nodosilinea nodulosa] 45.8 45.8 10% 0.007 33% WP_017301714.1

**>Contig_203_length_708_cov_1251.67_ID_405**

TCAGCCGAACATTTATCACGCCAGTTTTTACTCTTGGCACGACGTTCGTCGGCATCTGAAACGGCAAGGT

TGGAAACGCTTCGATTGATGTTGCATAAACCTCGGACACCGTGTCCGCTATCAAACGTGGTTTTCTTTAG

GTCTTGATAAGACTTGCGATAATCACGTTTGTCTTTTTTGCTTGCCTTCCGATGCTTAGTGAGTCCTTTC

TGGAGGGTTGCACCTGCATCAAGGTACTCTTTACGGCTTCGGTTGATTCCAAGTGCTTCATCAATCGTTT

GCTGCAAGATGACTGCAAATGATGCTTCCTGCATCGACTTGGCTACTGGGTAACCTTTGTCGGATGCAAG

TTTCACTAGGAGGGTTAAGTCGGTTTGGGTGATGATGGAGATGGGCTTAGAGTTGACTTCGGTCAAAAGG

TTGCCCCTCTTGGAAGTCTCGCCAGCTAAGGCTTTGAAGGCTTCCGAGTTAAGTAAGCGCCCCACGACTC

TTTTATCAATTTCCAATCCTCTGGACACGCCACGCAGGCTAAAAACCACCTGATTGGTGCTTGGGTTAAG

GAAACCCTGAAACACCACACCCAAAACGTTAAACCCTCCTGCTACTTCAGGAATGCGTTGTTGTTTAACC

ATTTGTTGACTCCTTTCTTCAGATTGAGTAAGTGAAAGAAGGGTCAACAGGTGAACCAAATTGTCAAGGT

ACAATGGA

BLASTx M.score T.score Q.cover E-value Ident Accession

Nuclear factor, kappa-B-binding prot. [Eufriesea mexicana] 40.8 40.8 27% 1.5 33% OAD62547.1

**Table S6 Spacer details and protospacers.** A = CRISPR array number. S# = spacer number. Length = basepairs of spacer sequence. Score = cut-off at default value of 20, however, values of 18 and 19 were considered if protospacer originated from BBD relevant match (e.g. cyanophage).

| A | S# | Sequence | Length [bp] | | Score | | Potential target | | Category | | |
| --- | --- | --- | --- | --- | --- | --- | --- | --- | --- | --- | --- |
| 1 | Spacer1 | CCAGAAAACCTTATCTCCCAAAAACCAGTAGGAGGCAGGA | 40 | | NA | | NA | | | NA | |
|  | Spacer2 | TTGTCCATTGGGAAAAGGAAGAATGCATTGTAGA | 34 | | 20 | | *Synechococcus* phage S-MbCM6 (NC_019444) | | | phage | |
|  | Spacer3 | AGTACCCATTCCTTCCGCAGAACCGCACTAACGCAA | 36 | | 23 | | *Salmonella* phage FSL SP-101 (KC139511) | | | phage | |
|  | Spacer4 | AAGCCTTTGAAATGGGCATGACCGAGCTAGAGGA | 34 | | NA | | NA | | | NA | |
|  | Spacer5 | GCAGGAGAGCGCAGATATCGAGCGGCTGTAGAGC | 34 | | 24 | | *Ruegeria* *pomeroyi* DSS-3 megaplasmid (NC_006569) | | | plasmid | |
|  | Spacer6 | GAGGTATAAATCCAGATACGATCCTGAAGGGCTGAGGGTTGC | 42 | | NA | | NA | | | NA | |
|  | Spacer7 | ACAAGGCAATTCCCAGAGATATAGATTTCAAG | 32 | | NA | | NA | | | NA | |
|  | Spacer8 | TTGATTAATCGGTCGATGGGATGAGCATTAGGATT | 35 | | NA | | NA | | | NA | |
|  | Spacer9 | GATAGATACCTGAAATGGTTGGAGGGGAAATCATGA | 36 | | 25 | | *Bifidobacterium* phage Bbif-1 (GQ141189) | | | phage | |
|  | Spacer10 | TTCAGGCAAAAACCTGTGATATTCAGGCTTGCCATTGGG | 39 | | 25 | | *Granulicella* *tundricola* plasmid pACIX905 (NC_015060) | | | plasmid | |
|  | Spacer11 | CGGGGGGATTGATGGGGTAAACGATCGCGTTTGCCAGTCGAT | 42 | | NA | | NA | | | NA | |
|  | Spacer12 | GGCAAGAAATATAATCACGGGTACTCTCACATGAGTTGAAA | 41 | | NA | | NA | | | NA | |
|  | Spacer13 | CCCCAAAAAACACAATCAGAAGAATCCCCAGAAGAATCCCAA | 42 | | 20 | | *Apocheima* *cinerarium* nucleopolyhedrovirus (NC_018504) | | | virus | |
|  | Spacer14 | CGTGCGAATTTCGTGCGAATTTCGTGCGAATTTCGC | 36 | | NA | | NA | | | NA | |
|  | Spacer15 | TTGCAGGATTAACAATTAATGGACGAAGCAAGGGCGATCGC | 41 | | NA | | NA | | | NA | |
|  | Spacer16 | CTCTTCTTTAACCCCGGACTGTTCCTCACCTTCTT | 35 | | NA | | NA | | | NA | |
|  | Spacer17 | TGGTCTTTTGCAGCCAATGCGCGCCAATTGCGAGCGTA | 38 | | NA | | NA | | | NA | |
|  | Spacer18 | AACAGTGATCGCGCTAGAGAATTGCAAGAATTAGA | 35 | | 24 | | *Stanieria* *cyanosphaera* PCC 7437 plasmid pSTA7437.02 (NC_019749) | | | plasmid | |
|  | Spacer19 | AATCTTTCCGACTTAACTTATGAGCTGAATTCTC | 34 | | NA | | NA | | | NA | |
|  | Spacer20 | TGAGCTAACCGCAGATCGGCTGGCAAAAATGATCAA | 36 | | 22 | | *Azospirillum* *lipoferum* 4B plasmid AZO_p1 (NC_016585) | | | plasmid | |
| 2 | Spacer1 | TTTACTGTAGCTCCATAATTCGAGTTATTGACGATATGCTT | 41 | | 27 | | *Anabaena* *cylindrica* PCC 7122 plasmid pANACY.01 (NC_019772) | | | plasmid | |
|  | Spacer2 | CTTAAACGCCAGCCGATTCATTTCGATAAATTTTTTGCT | 39 | | NA | | NA | | | NA | |
|  | Spacer3 | ATAACATTTCCGTCCGCTTATCTCGATGGTGCTGATTGGC | 40 | | 27 | | *Ralstonia* *eutropha* JMP134 megaplasmid (NC_007336) | | | plasmid | |
|  | Spacer4 | GATCGGGCCCCCATCTGGGAATGCCCCGAGTTT | 33 | | NA | | Contig_93, hypothetical protein | | | self-target | |
|  | Spacer5 | AATTTTATTTGGATCGGGTTTTGGGTGAGGATTTGCCTCT | 40 | | 21 | | Vibrio phage 11895-B1 genomic sequence (NC_020843) | | | phage | |
|  | Spacer6 | TAAAGGCAGTGTGCTGATCAAAGCTTTGTAATCAGC | 36 | | NA | | NA | | | NA | |
|  | Spacer7 | CCAAAGGGTCCCCACTTACTGGATGTCTTGAAATATCCA | 39 | | NA | | Contig_93, hypothetical protein | | | self-target | |
|  | Spacer8 | TCACCTGGTACGTCCAGCCCTTATCCTCTGTAAA | 34 | | 21 | | *Meiothermus* *silvanus* DSM 9946 plasmid pMESIL01 (NC_014213) | | | plasmid | |
|  | Spacer9 | TATTGGCTTGTAGCCTACCTCCTGCCCTTCATTACCGCCAAT | 42 | | NA | | NA | | | NA | |
|  | Spacer10 | TTTCCAATAAGGGGGTAATTGCCTCTTATGCCAA | 34 | | NA | | NA | | | NA | |
|  | Spacer11 | ATGATTGAATAGCTGGATAATTCTAATTGCCTTA | 34 | | NA | | NA | | | NA | |
|  | Spacer12 | GCTGCGGTGAGGCTGGCTTTTGTGCTATGGGTT | 33 | | 22 | | Beet curly top virus – California (NC_001412) | | | virus | |
|  | Spacer13 | TTCCGTATTCTTGCGAAATACATATTTCTTTTTTGTT | 37 | | 21 | | *Bacillus* *thuringiensis* Bt407 plasmid BTB_502p (NC_018878) | | | plasmid | |
|  | Spacer14 | CCGCGTATTTGGAATCCAACGATGACCCGATATGA | 35 | | NA | | NA | | | NA | |
| 3 | Spacer1 | GAGAAAGTGAAACAACGAAGCTTTTCAGCGATTAGCCCCC | 40 | | NA | | NA | | | NA | |
|  | Spacer2 | AAGAAAGCCTTGGACGATACTGAGGCAACTGTGGAT | 36 | | 20 | | *Escherichia* *coli* K-12 plasmid F DNA (NC_002483) | | | plasmid | |
|  | Spacer3 | TATGAAGAAAAGATGGAGCATCTCAACGCAGCAAT | 35 | | 24 | | *Spodoptera* *frugiperda* MNPV virus (NC_009011) | | | virus | |
|  | Spacer4 | AGAGATTTTAAGAAATGCAAAGCAACCAATGACAGCAAACTTG | 43 | | NA | | NA | | | NA | |
|  | Spacer5 | GTTTTCTCAGGACTATGGGGGCGAAAGTTTTCACTTAT | 38 | | 23 | | *Acaryochloris* *marina* MBIC11017 plasmid pREB2 (NC_009927) | | | plasmid | |
|  | Spacer6 | TTTTATTTTGCACTGAAGTACTCCCCTTCTCGTTCGTATT | 40 | | 23 | | *Leptospira* phage LnoZ_CZ214 (KF114877) | | | phage | |
|  | Spacer7 | TTTAATGGCGCTTACGCCATTTGCAATTTATATCTCATT | 39 | | NA | | NA | | | NA | |
|  | Spacer8 | AGGGACTTCCTGCACGATGCCTTCTTCAAGCATTTC | 36 | | NA | | NA | | | NA | |
|  | Spacer9 | TGCAACGCCAAATTGAGAGAGATGTGGCCCGCCAGT | 36 | | NA | | NA | | | NA | |
|  | Spacer10 | ATCCAACATCCTCTGAAGGCTGTACCACGGGATGTATGA | 39 | | NA | | NA | | | NA | |
|  | Spacer11 | CAAATTTTCTACTTGCAAGCGCTCCATACTTTCAA | 35 | | 28 | | *Sclerotinia* *sclerotiorum* dsRNA mycovirus-L (NC_017915) | | | virus | |
|  | Spacer12 | CAAACCTATTTTTACTATTTCCGCAATTTATGGCAA | 36 | | NA | | Contig_93, hypothetical protein | | | self-target | |
| 4 | Spacer1 | GCTACTTTTGACATTACTGTGTCCGGCACCCC | 32 | | 19 | | *Cyanobacterium* *aponinum* PCC 10605 plasmid pCYAN10605.01 | | | plasmid | |
|  | Spacer2 | CTAGAGACAGAAAATTACTCGAGGAAAAAATCATGTCT | 38 | | 22 | | *Burkholderia* *phymatum* STM815 plasmid pBPHY01 (NC_010625) | | | plasmid | |
|  | Spacer3 | TTGAAGTGACTGGAAGGGAACGTATCGTCACTGG | 34 | | NA | | NA | | | NA | |
|  | Spacer4 | TTACCGCCTACCTTACTAGGACGGTATATAGAGA | 34 | | NA | | NA | | | NA | |
|  | Spacer5 | CCAACACACCCAAGCGGGTGCAATTCTGTGAGGAGATGCGGGGC | 44 | | 18 | | *Cyanophage* KBS-M-1A genomic sequence (NC_020836) | | | phage | |
|  | Spacer6 | GGCACCGCGTACAAAATTTCCGTGGACATCTTTA | 34 | | NA | | NA | | | NA | |
|  | Spacer7 | TTACCTCCAGATTGCCCCTGATTTGGTAAAACCATCTCTTAA | 42 | | NA | | NA | | | NA | |
| 5 | Spacer1 | TCAGGTAGACAAAGTGAGGTTAAGCGCCTCCCATTA | 36 | | NA | | NA | | | NA | |
|  | Spacer2 | TCTTGCCATAAGAGATTTAATGATGTCAAATCAAGA | 36 | | NA | | NA | | | NA | |
|  | Spacer3 | ACTAACAAGCTTTTATCCTCATGCTTATGAAGAA | 34 | | 22 | | *Loktanella* phage pCB2051-A genomic sequence (NC_020853) | | | phage | |
|  | Spacer4 | TGCCCCCACCAGCCGAAGCGAACAGCAAACCCAC | 34 | | 24 | | *Micrococcus* sp. V7 plasmid pLMV7 (NC_022599) | | | plasmid | |
|  | Spacer5 | AGATTATGATAAGTCAAATGCGGGCAAAGCCCAACCCCT | 39 | | NA | | NA | | | NA | |
|  | Spacer6 | GAAACATTCAGAATCAAATGGGAGGTTGATTGGGA | 35 | | NA | | Contig_93, hypothetical protein | | | self-target | |
|  | Spacer7 | CCGGCACCAGATTTTCCGGATGAATTCCTAGAAG | 34 | | NA | | Contig_93, hypothetical protein | | | self-target | |
|  | Spacer8 | GCGTATGGACACCTTTTCATCACCATTGATATGTT | 35 | | 20 | | *Acidianus* two-tailed virus complete viral genome (AJ888457) | | | virus | |
|  | Spacer9 | GGGAAATTCTCCTTTCGGAGCGCTCGGTGAAATTCTTG | 38 | | NA | | Contig_93, hypothetical protein | | | self-target | |
|  | Spacer10 | ATTAAATTTTATTTCCATTTCAAAGTTCCACAACCCAT | 38 | | 21 | | *Staphylococcus* phage StauST398-3 (NC_021332) | | | phage, temperate | |
|  | Spacer11 | CAACATTATTTGGGACATAGAAATATCAGGAACACCTTGATA | 42 | | 30 | | *Cyanothece* sp. PCC 7424 plasmid pP742401 (NC_011738) | | | plasmid | |
| 6 | Spacer1 | CAAAAGATTCGCCAGTCACCAATGCCTGAGTTAC | 34 | | NA | | NA | | | NA | |
|  | Spacer2 | GCAATAAGAATCAGGATGACAACCGCCCAAAATCCGATAA | 40 | | 24 | | *Sinorhizobium* *fredii* HH103 plasmid pSfHH103e (NC_016815) | | | plasmid | |
|  | Spacer3 | CTCCTCCGACTAGAACACTACAATGCCCATCAACTGCTGGA | 41 | | NA | | NA | | | NA | |
|  | Spacer4 | CCAAGCCCTCACCGCAGCCGCCTATAGTGGTC | 32 | | 21 | | *Paracoccus* *aminophilus* JCM 7686 plasmid pAMI5 (NC_022043) | | | plasmid | |
|  | Spacer5 | AAGTAATTACCAGAAAGACATATTGGAGTGGGTGAA | 36 | | 21 | | *Erwinia* *amylovora* CFBP 2585 plasmid pEA3 (NC_020920) | | | plasmid | |
|  | Spacer6 | TGAAAAACTAGAAACGGCAACGGCTCGTGCTATGCAAA | 38 | | 20 | | Enterobacteria phage EK99P-1 (KM233151) | | | phage, virulent | |
|  | Spacer7 | AGTGGATCTGGTGGCGATCGCTAAAATAATGGTAGA | 36 | | NA | | NA | | | NA | |
|  | Spacer8 | GGCAGAACCTACTACATCCGTAAGAAAATCGCCCTG | 36 | | NA | | NA | | | NA | |
|  | Spacer9 | CTTGAGCAATTAGGCTTGCCCAAGGTTGCCATAAT | 35 | | NA | | NA | | | NA | |
|  | Spacer10 | TAATCACCACAACATCAGGACTGACGATTCTAA | 33 | | NA | | NA | | | NA | |
|  | Spacer11 | CTGGGTTAAGAAGAGGATTCCATTCGCTATAATCAAAATCAT | 42 | | NA | | Contig_93, hypothetical protein | | | self-target | |
|  | Spacer12 | TTGTCATCAAGATATCCCCACGCCTAGCGAAATCTT | 36 | | NA | | NA | | | NA | |
|  | Spacer13 | CTCTTCCTCGCCTATTGGGGAAAACCAGACCCCGGT | 36 | | NA | | NA | | | NA | |
|  | Spacer14 | GCATAACAAAATACGATCGGGAAACATTGAAGAGCCCAAT | 40 | | NA | | Contig_93, DnaB domain-containing helicase,  VIRsorter: Phage_cluster _71 _PFAM-AAA_25 | | | self-target | |
|  | Spacer15 | CGCACTGGCCGATAGAAATCATTTTCATCGCCAAGGG | 37 | | 26 | | *Cyanothece* sp. PCC 7822 plasmid Cy782202 (NC_014534) | | | plasmid | |
|  | Spacer16 | TGGCTATAATTCTCTCTGGATAAGGGTTTCAAGCTTT | 37 | | 23 | | Uncultured bacterium plasmid pEFC36a (NC_025088) | | | plasmid | |
|  | Spacer17 | CACAAAAATGGGGGATAGCTTACTTCCCCCTGCGA | 35 | | NA | | NA | | | NA | |
|  | Spacer18 | TCTGGGAACGAGGTGGAGTCGCTGGGGATGGTTTCGAGT | 39 | | NA | | NA | | | NA | |
|  | Spacer19 | AGACGGATTAATCGAACTCAGTAACGACATCCAA | 34 | | 38 | | *Natrialba* phage PhiCh1 (NC_004084) | | | phage,  temperate | |
|  | Spacer20 | GTGTAGCTTTACAGGTCGCACTTATGGAATGGAAA | 35 | | NA | | NA | | | NA | |
|  | Spacer21 | CAAGCGCTTTTGGGGGATGGATGCCGTGGGGAGTT | 35 | | NA | | NA | | | NA | |
|  | Spacer22 | GAAGAATTGTCAACAGAAGATGCCGAATATGTGGC | 35 | | 19 | | *Synechococcus* phage S-ShM2 (NC_015281) | | | phage, virulent | |
|  | Spacer23 | TTGCCCTAAATTGTTTTTTAGTCTTCTGCGATAATC | 36 | | 26 | | *Staphylococcus* *epidermidis* plasmid pSWS47 (NC_022618) | | | plasmid | |
|  | Spacer24 | CAAAAAGCGATCATTTCCCATCGCTCTATGGAGCGATCGCAC | 42 | | NA | | NA | | | NA | |
|  | Spacer25 | AATTTAGAAACCTTGATGCCTTGATCGCGCCCTACCG | 37 | | NA | | NA | | | NA | |
|  | Spacer26 | GTGTCAAAATGGCAAGTGCGCCCACTTCCTCAA | 33 | | NA | | NA | | | NA | |
|  | Spacer27 | AGACAAAGAATACAGCGAAGGCGAACCGAGCGAACCA | 37 | | NA | | NA | | | NA | |
| 7 | Spacer1 | TGGAGGATTTGGACTGGCATCGAGAAAAAGTACA | 34 | | 23 | | *Sinorhizobium* *fredii* NGR234 plasmid pNGR234b (NC_012586) | | | plasmid | |
|  | Spacer2 | TTACATTGCCTATTCTTTTGAGCAAGATGATGACT | 35 | | 24 | | Influenza A virus 107399 (H9N2) (NC_004911) | | | virus | |
|  | Spacer3 | TATCTATGAGCCAGGTATTTCCGATGAAGAATGTTATA | 38 | | NA | | NA | | | NA | |
|  | Spacer4 | GTATATTTTCCTCCCAGATTGGCTAAGGAGTAACT | 35 | | 20 | | *Methanobacterium* *formicicum* DSM 3637 (NZ_AMPO01000001) | | | NA | |
|  | Spacer5 | TGCAGGAGGAAAATAGGGGAAGGACATGGTTGATAGA | 37 | | 19 | | *Synechococcus* phage Syn19 (NC_015286) | | | phage, virulent | |
|  | Spacer6 | CTTGAGATTCGTTGATGTAGGTTTGAAGCCTACCG | 35 | | NA | | NA | | | NA | |
|  | Spacer7 | ATGAAAAGTACTGACAACTATTAATACTGTACAAA | 35 | | NA | | NA | | | NA | |
|  | Spacer8 | ATCATATTAAATTCACTCATGCCATTATTTCTGCTCAG | 38 | | NA | | NA | | | NA | |
|  | Spacer9 | GAAGATAGAAATCTAAATCAGTCTGCAAGCTTTGAGT | 37 | | NA | | NA | | | NA | |
|  |  |  |  | |  | |  | | |  | |
| Geitlerinema sp. BBD_1991 | | | |  | |  | |  | | |  |
| A | S# | Sequence | Length [bp] | | Score | | Potential target | | | Category | |
| 1 | Spacer_NoG1_1 | GTCTTCGTCGTTCTGAGAAGAGTGCCAGAGGTAG | 34 | | NA | | NA | | | NA | |
|  | Spacer_NoG1_2 | CTGCGGCGCTTCTTCGTTCCCGAGAAGTTGCGATCGGACG | 40 | | 22 | | *Cupriavidus* *metallidurans* CH34 megaplasmid | | | plasmid | |
|  | Spacer_NoG1_3 | CTTCAATCGTTACGTCCCCCCCCCTCAGCTTCCACGA | 37 | | NA | | NA | | | NA | |
|  | Spacer_NoG1_4 | CTGCCTTGATGGGGGACTCAAACGCCATCACCCG | 34 | | NA | | NA | | | NA | |
|  | Spacer_NoG1_5 | CGGAGATGCTCGCCGTATTGGCTCTGCAAAACCG | 34 | | NA | | NA | | | NA | |
|  | Spacer_NoG1_6 | AAGTCGAGCCCATTGTTGTTGAATGCACGGAGGAGGA | 37 | | NA | | NA | | | NA | |
|  | Spacer_NoG1_7 | TCGAAAATCTCCGAATTCAGTTTTGATGATAAATTAGTA | 39 | | NA | | NA | | | NA | |
|  | Spacer_NoG1_8 | GTTACGTCAGAACTCCCAGATCGGCGCATTCAGG | 34 | | NA | | NA | | | NA | |
|  | Spacer_NoG1_9 | TTTATCGGATTTAGAGCCGAGTGCCTCGCATTGTTCCAATATGTTCTG | 48 | | 20 | | KT895374 *Bacillus* phage vB_BpuM-BpSp | | | phage | |
|  | Spacer_NoG1_10 | TCCTTATCGACTTCTACCCCAAATCCCCACCC | 32 | | NA | | NA | | | NA | |
|  | Spacer_NoG1_11 | CTGGGATAATCGGGGGCGGAAACCCGCAAAACAA | 34 | | NA | | NA | | | NA | |
|  | Spacer_NoG1_12 | TAAAGAAAAAAACCTCGAAACCTATGGGAACTAAC | 35 | | NA | | NA | | | NA | |
|  | Spacer_NoG1_13 | CTTGAGGTCGGCGAAAGGTAAACGATCGCGAATCCCG | 37 | | NA | | NA | | | NA | |
|  | Spacer_NoG1_14 | CCCCCTGATATCTTCCGTATCAGGGGGGTAAGGAG | 35 | | NA | | NA | | | NA | |
|  | Spacer_NoG1_15 | ACATCGGCACGTGACGGCGAAAAATATCTCAAAC | 34 | | NA | | NA | | | NA | |
|  | Spacer_NoG1_16 | CAGACAACAATCGGCAATTTCCAGTCGAGGCAGATTTTC | 39 | | NA | | NA | | | NA | |
|  | Spacer_NoG1_17 | AACAGTTGAGCTTCGACACCGAGTCAATCGATCTGTC | 37 | | NA | | NA | | | NA | |
|  | Spacer_NoG1_18 | GTTGTGTTCGGTGCAGCAACACTACACCGAACAC | 34 | | NA | | NA | | | NA | |
|  | Spacer_NoG1_19 | TCTGATGCTGAACTTCAGGCCGAAATTCATGCGATG | 36 | | NA | | NA | | | NA | |
|  | Spacer_NoG1_20 | GGCTCTAATCGAGTCTCATACCATCGCGCCAAATCCAA | 38 | | NA | | NA | | | NA | |
|  | Spacer_NoG1_21 | ACCTTCGATATGATGCTCGTCATCATGAAA | 30 | | NA | | NA | | | NA | |
|  | Spacer_NoG1_22 | GGTCGGACGGACAAGCCAGTAGTCTCGCCCTCCTC | 35 | | NA | | NA | | | NA | |
|  | Spacer_NoG1_23 | GCGTTGTTTCCAGCCTATCGCAGTTTGCAACAGAT | 35 | | NA | | NA | | | NA | |
|  | Spacer_NoG1_24 | CCCGTTGCTGAAGTCGGGCGCGATGTAAGCCGCCC | 35 | | NA | | NA | | | NA | |
|  | Spacer_NoG1_25 | TGCCCTTATTTCGCCTCCTACCACCGCTCTGCCACCATCCA | 41 | | 21 | | *Halobacterium* sp. JI20-1 plasmid: II | | | plasmid | |
|  | Spacer_NoG1_26 | GGGGACAAAGAGCGGGTTCGGGATTACTGCTTT | 33 | | NA | | NA | | | NA | |
|  | Spacer_NoG1_27 | ATTCGCCAAATTCGCCACCTGTCACGGCATCACG | 34 | | NA | | NA | | | NA | |
|  | Spacer_NoG1_28 | TCTTTATCACGCATGGATAAGTGACGATGTTCACC | 35 | | NA | | NA | | | NA | |
|  | Spacer_NoG1_29 | GAGGCATGGCGATCGTTGTACTGACCGATGGCGATTC | 37 | | NA | | NA | | | NA | |
|  | Spacer_NoG1_30 | GGGTCGGCCAGGATTGAGGTTTGCTTTCACCTCAAATTT | 39 | | NA | | NA | | | NA | |
|  | Spacer_NoG1_31 | TCGGTTAGCGTTACGGCTCCATCCGCAGATTTCACCAC | 38 | | NA | | NA | | | NA | |
|  | Spacer_NoG1_32 | CTTCGAGGAAGATAAGATTTTCCAAGTCGATCTCCA | 36 | | NA | | NA | | | NA | |
|  | Spacer_NoG1_33 | TCGAAGCCCTTGCCAAAGGGGCAGGCTTTGACCACCTGGAG | 41 | | NA | | NA | | | NA | |
|  | Spacer_NoG1_34 | CAGTCTTTCACATGGCTATTACGCCAGCGATCGAC | 35 | | NA | | NA | | | NA | |
|  | Spacer_NoG1_35 | TTGCAAGCCTTGGCACTGGACCGGACGGGTACC | 33 | | NA | | NA | | | NA | |
|  | Spacer_NoG1_36 | CTTCACACGGAAGCGGGTCTGAGGAGTCGCCAAGCTTCAG | 40 | | NA | | NA | | | NA | |
|  | Spacer_NoG1_37 | AGTCAATACACAAATATAGTCTATGAACAGATTAA | 35 | | NA | | NA | | | NA | |
|  | Spacer_NoG1_38 | TCTAATTAGTTCTAAAGCTCTATTAGATCCTTTGGC | 36 | | NA | | NA | | | NA | |
|  | Spacer_NoG1_39 | TATAATGGTCATCGGGAAAGAGTGCGGGTATAGCAA | 36 | | NA | | NA | | | NA | |
|  | Spacer_NoG1_40 | TACTACTTCTTATGCTCTAGAGATTCAAAGAATCAA | 36 | | NA | | NA | | | NA | |
|  | Spacer_NoG1_41 | TGTAACAGGGAAGAACAGCCGGATCAATAATGTAAAGATCC | 41 | | NA | | NA | | | NA | |
|  | Spacer_NoG1_42 | TAATGCATGATTTTTGTATTAAGGTAATCTTTTCT | 35 | | NA | | NA | | | NA | |
|  | Spacer_NoG1_43 | ATAGAAGAGAGAGAGGAGATTTTAAAAGCTATT | 33 | | NA | | NA | | | NA | |
|  | Spacer_NoG1_44 | CTTAATCCTATAGTTTCAAATGTTACGTTAAGCGCTATTCC | 41 | | NA | | NA | | | NA | |
|  | Spacer_NoG1_45 | AGGCATTAAATACTTGAAAGGCTAAATAGCCTTG | 34 | | NA | | NA | | | NA | |
|  | Spacer_NoG1_46 | GAAAACTGGTTAAGAAATAATGGCTAAAGTTTTT | 34 | | 20 | | C2PVCG_L48605 Bacteriophage c2 | | | phage | |
|  | Spacer_NoG1_47 | GATTTATTACTTACATCCCCGGCATCAGGCATATG | 35 | | NA | | NA | | | NA | |
|  | Spacer_NoG1_48 | CTTAGGGGAGGCTAACTGAGATGACTAGATACGTTTTA | 38 | | NA | | NA | | | NA | |
|  | Spacer_NoG1_49 | TGCATTCGCCCAAGCTTGGGAATCACCGAAGTCGAT | 36 | | NA | | NA | | | NA | |
|  | Spacer_NoG1_50 | AACAAGCTTTCCTTAAGACTTTAGAAGATGCCCCTA | 36 | | NA | | NA | | | NA | |
|  | Spacer_NoG1_51 | CTTATACGAAGGGGTATTGCAATCTTACAGAG | 32 | | NA | | NA | | | NA | |
|  | Spacer_NoG1_52 | CAAAAGTCTTTACATTTCCAGAGAAATCTAATAT | 34 | | NA | | NA | | | NA | |
|  | Spacer_NoG1_53 | GCGAGTAAGCAGGTAAAGTTACCAGATTATGGAAATTT | 38 | | NA | | NA | | | NA | |
|  | Spacer_NoG1_54 | GGGAGTTTCGGCAACGCCCCTGTCGAAGTTGTTCTTGGT | 39 | | NA | | NA | | | NA | |
|  | Spacer_NoG1_55 | AGTGAATATTTGAAGCTTCCACCGTTGGAAAAAACT | 36 | | NA | | NA | | | NA | |
|  | Spacer_NoG1_56 | GGGAACTAACTGGCGATACGCCGGGTACGAGCC | 33 | | NA | | NA | | | NA | |
|  | Spacer_NoG1_57 | AGCATCTACATAGTCAGATTGTGCGGTTAGGTACAG | 36 | | NA | | NA | | | NA | |
|  | Spacer_NoG1_58 | TTTAGTTTTTATGTCCGGCTCATTAGTATCCTACTT | 36 | | NA | | NA | | | NA | |
|  | Spacer_NoG1_59 | CCACCCTCATATATGGCCCGCCTGGTTCGGGGAAATC | 37 | | NA | | NA | | | NA | |
|  | Spacer_NoG1_60 | TCGCGATCTGTGACGAGGGTGTTGGGTTGAAATGG | 35 | | NA | | NA | | | NA | |
|  | Spacer_NoG1_61 | TAAAGGTGAGCTATTATAGTGTACCTTTCCACCCT | 35 | | NA | | NA | | | NA | |
|  | Spacer_NoG1_62 | GATTAAAGCCATCACTATTATAAGCATTGAAAGTCTC | 37 | | NA | | NA | | | NA | |
|  | Spacer_NoG1_63 | TCAAAATCGTTGAAAGACTTTGCTGGTGGTAGTTT | 35 | | NA | | NA | | | NA | |
|  | Spacer_NoG1_64 | ATATATATTGGTAATCCGTCAGCGTCATTTACTTT | 35 | | NA | | NA | | | NA | |
|  | Spacer_NoG1_65 | CCTTAAATTTTTTTGTGTTAAAATAGTTAATATAT | 35 | | 21 | | *Staphylococcus* *aureus* NCTC8532, plasmid: 2 | | | plasmid | |
|  | Spacer_NoG1_66 | TTAAGTCAACCTATAACTTCAGCACCTAAAATAAACCA | 38 | | NA | | NA | | | NA | |
|  | Spacer_NoG1_67 | GTAAGATTTTGAGCTAATACCCTGTAAGTCTTAGA | 35 | | NA | | NA | | | NA | |
|  | Spacer_NoG1_68 | ATTTGGTTAGAATCGTTCTCGACGTAGTAAAATTT | 35 | | NA | | NA | | | NA | |
|  | Spacer_NoG1_69 | ATTAGAGCTAGTTGGTAAATCATCCAATCCACACATCAG | 39 | | NA | | NA | | | NA | |
|  | Spacer_NoG1_70 | GAAAGGCTCTTTTGCCCCTAAATCAGTCTTGTTTGTT | 37 | | NA | | NA | | | NA | |
|  | Spacer_NoG1_71 | AAGTATAAGCATTTTTGGTATTGACACCTTAATCTCA | 37 | | NA | | NA | | | NA | |
|  | Spacer_NoG1_72 | TAGATTCCACCACTCGGGTAGTTTACAACAAATCT | 35 | | NA | | NA | | | NA | |
|  | Spacer_NoG1_73 | TTTCTATAATTTTTGAGTTCTTTAGAATAATAATTTTT | 38 | | 22 | | JN258408_JN258408 Megavirus chiliensis | | | virus | |
|  | Spacer_NoG1_74 | AGTGCCGCACCAAATCCCCAAGGTTCACCAGAAA | 34 | | NA | | NA | | | NA | |
|  | Spacer_NoG1_75 | TCAGAAGCCGTTTGGCTCCCTTGGTGCCTTTCGCTTG | 37 | | 21 | | *Meiothermus* *silvanus* DSM 9946 plasmid pMESIL01 | | | plasmid | |
|  | Spacer_NoG1_76 | TTAATATCTATCGCGAAGCGATCGCACAATGCCTTAAATCT | 41 | | NA | | NA | | | NA | |
|  | Spacer_NoG1_77 | CTCCAGCGGCAGAGGGTTGGCAATCGGTTCGTCA | 34 | | 20 | | *Escherichia* *coli* ST131 EC958 plasmid pEC958 | | | plasmid | |
|  | Spacer_NoG1_78 | AATTCGGGGCTGTACTCCTCTAGCAGTAATCCTG | 34 | | NA | | NA | | | NA | |
|  | Spacer_NoG1_79 | CTTTTAAGATTGGGTTCACGGCTACCCCTTACCG | 34 | | NA | | NA | | | NA | |
|  | Spacer_NoG1_80 | ACGAAGAAGTATCGCGCTGGGTGACGGGATTTTGG | 35 | | NA | | NA | | | NA | |
|  | Spacer_NoG1_81 | TTTGCCGCTCCAATTCCTCCAGCTTGCGTTGGAGTGCCAG | 40 | | NA | | NA | | | NA | |
|  | Spacer_NoG1_82 | GTCTGCAAAGATGCACTCAGGGTTATTTACTAACTG | 36 | | NA | | NA | | | NA | |
|  | Spacer_NoG1_83 | GATTGTACAAAATCAACGGGAAGAATTAGAAAATAGAA | 38 | | NA | | NA | | | NA | |
|  | Spacer_NoG1_84 | TTACTTTAATCAATCCCTCCCAATTACCTCCAATAGATG | 39 | | NA | | NA | | | NA | |
|  | Spacer_NoG1_85 | TTTTGTCCGATTCGATCGAGAGACTCTTTTGCCTCCCGATCGACT | 45 | | NA | | NA | | | NA | |
|  | Spacer_NoG1_86 | AAAATTCCTCCGATCGCCAAGCCGAAACCGACACC | 35 | | NA | | NA | | | NA | |
|  | Spacer_NoG1_87 | TTGTGCTTCCGATTGTGGCTTCTTACTTACTTCAATCGCCTC | 42 | | NA | | NA | | | NA | |
|  | Spacer_NoG1_88 | TTGACGGAAGGGTTGAAATCGCTAAAGGCGTCTGCGA | 37 | | NA | | NA | | | NA | |
|  | Spacer_NoG1_89 | TCGATCTCCGTCACGCAAGGTATATCCATTTGTGA | 35 | | NA | | NA | | | NA | |
|  | Spacer_NoG1_90 | TTTGTTTGGTACAGATTGATTATTAGAACCTGG | 33 | | 21 | | HM595733 *Spodoptera* *frugiperda* MNPV isolate Nicaraguan | | | plasmid | |
|  | Spacer_NoG1_91 | TTCATAGCCAGGCATGATTGACCAGAGTGATTTGCCATT | 39 | | NA | | NA | | | NA | |
|  | Spacer_NoG1_92 | TCCTTTTCTACTCCACAATGTTGAATTAAAGTCTC | 35 | | NA | | NA | | | NA | |
|  | Spacer_NoG1_93 | CCTTAAGAGAACTGCAATCTGGACACTTAAGTCCAGA | 37 | | NA | | NA | | | NA | |
|  | Spacer_NoG1_94 | TTTATTTGTTCATAAATTACATTAGTACTAAGGGTT | 36 | | NA | | NA | | | NA | |
|  | Spacer_NoG1_95 | GAGGTCGTAACTTATGATAGAATTCTAGATGAATATTT | 38 | | NA | | NA | | | NA | |
|  | Spacer_NoG1_96 | CATCTTTATATCCGCTGGCAGCTTTGCCACCTAGACCG | 38 | | NA | | NA | | | NA | |
|  | Spacer_NoG1_97 | GTTCTGTCTGTTTTTGAAGCAGCCGCGATAAGAA | 34 | | NA | | NA | | | NA | |
|  | Spacer_NoG1_98 | GTAGCAAAGGAAGCTTCCTTTGCTATCGAGACAA | 34 | | NA | | NA | | | NA | |
|  | Spacer_NoG1_99 | GCAACGGGAAAAAGGAAAAATGCAATCATCCCTA | 34 | | NA | | NA | | | NA | |
|  | Spacer_NoG1_100 | CGGTTCTCGACGATCGCGGGTTCCCCCAGTTGCAT | 35 | | NA | | NA | | | NA | |
|  | Spacer_NoG1_101 | GAAAGAGGACTATCAGATCCTCTCCAACGGGCAACGG | 37 | | NA | | *Sphingobium* sp. EP60837 plasmid pEP2 | | | plasmid | |
|  | Spacer_NoG1_102 | TCCACGCCGTCCAGCTCCCCACGGTGGCTGTAAATCC | 37 | | NA | | NA | | | NA | |
|  | Spacer_NoG1_103 | TTGATGCTTGAGTGCGAAATTTTCTATCCCTTTGA | 35 | | NA | | NA | | | NA | |
|  | Spacer_NoG1_104 | GTTATAGGGACGACGACTTATCCGAGGACTGGAA | 34 | | NA | | NA | | | NA | |
|  | Spacer_NoG1_105 | TGCAAAGTGCGATTGGCTGTGGGAGGCAATTCAA | 34 | | NA | | NA | | | NA | |
|  | Spacer_NoG1_106 | CTTATTGGTATCAGCGAGCTAGGTTGGCTCTCACTGATTTG | 41 | | NA | | NA | | | NA | |
|  | Spacer_NoG1_107 | TCTGTTACCCAATTTGGAGCGGGATCGAGGACGATCTC | 38 | | NA | | NA | | | NA | |
|  | Spacer_NoG1_108 | CCGTACCATAGAGATAGCGCGATCGAGGCAAAACTC | 36 | | NA | | NA | | | NA | |
|  | Spacer_NoG1_109 | GCCGAGTTGAAGGCGATCGCCGCCAAACTGATGG | 34 | | 20 | | *Rhizobium* sp. N541 plasmid pRspN541e | | | plasmid | |
|  | Spacer_NoG1_110 | TCGAACACGGGCAAGGCAGCCCCCTCAACGTCTTC | 35 | | NA | | NA | | | NA | |
|  | Spacer_NoG1_111 | GAGAAGAAGTTCGTCACGGGCGGCGCAGTTGGCGAGACT | 39 | | NA | | NA | | | NA | |
|  | Spacer_NoG1_112 | ACCAACAGACGGAGCCACGCCAGACACCCCAGACGGA | 37 | | NA | | NA | | | NA | |
|  | Spacer_NoG1_113 | GGATAAAGGACAGATGAATATCGATAACTTTCTT | 34 | | NA | | NA | | | NA | |
|  | Spacer_NoG1_114 | TCTAAAAATACATCCCACCAGTGACCGAAATCAA | 34 | | NA | | NA | | | NA | |
|  | Spacer_NoG1_115 | CCAGCGCCGTGGCGCTTGCCCCCGTGTCCGTTA | 33 | | NA | | NA | | | NA | |
|  | Spacer_NoG1_116 | CTTTGCTATCGCTGTAGAGCAAGCAAAGGATACG | 34 | | NA | | NA | | | NA | |
|  | Spacer_NoG1_117 | GAAGGTAACGCTCGGCCGGGGCGATTGTTTTGGCTCG | 37 | | NA | | NA | | | NA | |
|  | Spacer_NoG1_118 | TTGTAGGTAAACACACTCCTCAATCAGATCTAGGATCT | 38 | | NA | | NA | | | NA | |
|  | Spacer_NoG1_119 | TTGGAACAACTTGGGGACTAGAAATCATTGGATTAGGTAA | 40 | | 20 | | *Clostridium* *botulinum* 202F plasmid pCBI | | | plasmid | |
|  | Spacer_NoG1_120 | TTAAAGTCCCCCATCCTAGAACTGGAAATTAAG | 33 | | NA | | NA | | | NA | |
|  | Spacer_NoG1_121 | GAGAAACAAATACCCAATTTAAGGGCGCAAGTTGCTCAG | 39 | | NA | | NA | | | NA | |
|  | Spacer_NoG1_122 | CTGAAGGTTATGCACCTTATTATGTTAGGTACGGAGTATTT | 41 | | NA | | NA | | | NA | |
|  | Spacer_NoG1_123 | TCGGAATGATACTCGTCAAGCCTTCCTGTCCCGT | 34 | | NA | | NA | | | NA | |
|  | Spacer_NoG1_124 | TTGATTCAAAGAATCATTTACGGTAGTATCAAAG | 34 | | NA | | NA | | | NA | |
|  | Spacer_NoG1_125 | GGGTTCAAGCTTTAGAGTACATTCTCAAGTCTGCTACC | 38 | | NA | | NA | | | NA | |
|  | Spacer_NoG1_126 | ACGAGATTTTCATTAAATTTTATAATGGAGATATTTTCTTA | 41 | | 21 | | *Peptoclostridium* *difficile* NCTC13307, plasmid: 2 | | | plasmid | |
|  | Spacer_NoG1_127 | ATATTTTCGATTAGTTCATACATCGCTAAATTCAGATG | 38 | | NA | | NA | | | NA | |
|  | Spacer_NoG1_128 | GGAGATATGTGGAGTGATTTAATCCACGCCCACATC | 36 | | NA | | NA | | | NA | |
|  | Spacer_NoG1_129 | TTTGCGAATGAGACTACGGGTTTCATGGTAGATA | 34 | | NA | | NA | | | NA | |
|  | Spacer_NoG1_130 | TCTGCCCCGATCTGGAGGGGTATCTTTTAGAGAATCCA | 38 | | 20 | | *Mycobacterium* *chubuense* NBB4 plasmid pMYCCH.01 | | | plasmid | |
|  | Spacer_NoG1_131 | TTCTACGCCTCCACCCCGTCGTCGGAAAACTACGCCAAATTC | 42 | | 22 | | *Granulicella* *tundricola* MP5ACTX9 plasmid pACIX902 | | | plasmid | |
|  | Spacer_NoG1_132 | CTCAAAAGTTTTATCTGAGAGAGGGGAGCCTTTT | 34 | | NA | | NA | | | NA | |
|  | Spacer_NoG1_133 | GGAAATTGGCTTCGGGGAACCAGCAGACAACACT | 34 | | NA | | NA | | | NA | |
|  | Spacer_NoG1_134 | TAACCTATTTAAAAGAGGATGATTAATATGAT | 32 | | 22 | | *Clostridium* *botulinum* plasmid pCB111 DNA strain: 111 | | | plasmid | |
|  | Spacer_NoG1_135 | GCTTAACAAAGCCATCTAAGTTAGTAGTGTCTTTGTAAGA | 40 | | NA | | NA | | | NA | |
|  | Spacer_NoG1_136 | ATTGATGGCTGTTCCGACTATAGATAGGTCGTAGTTATCTAA | 42 | | NA | | NA | | | NA | |
|  | Spacer_NoG1_137 | TTGAAACTGAATCTTATAGGTTCACAGGAAATTAT | 35 | | NA | | NA | | | NA | |
|  | Spacer_NoG1_138 | TCGGTAAGACCCATCCTCGAAAAGTTCAAGTGCGT | 35 | | 21 | | KU568494 *Mycobacterium* phage Bactobuster | | | phage | |
|  | Spacer_NoG1_139 | TATGCCTGGATGGACAGCCCGTATTGCACGCACTTCT | 37 | | NA | | NA | | | NA | |
|  | Spacer_NoG1_140 | GATGAGCCACTAGATAGTCCATATTAGCTAGAAAA | 35 | | NA | | NA | | | NA | |
|  | Spacer_NoG1_141 | TCGTTTTTACCTCAATGCTATTGAGGAGATGGATTGT | 37 | | NA | | NA | | | NA | |
|  | Spacer_NoG1_142 | GTATCCCTAGATCTCGTTAATCTAGGTGATGTCA | 34 | | NA | | NA | | | NA | |
|  | Spacer_NoG1_143 | GATGGAGAAGAAATTTACTCTGCATCGTTCGCAGGG | 36 | | NA | | NA | | | NA | |
|  | Spacer_NoG1_144 | TAGATGTAGAGAATCTTATAGCCCAAACTTAAAAATA | 37 | | NA | | NA | | | NA | |
|  | Spacer_NoG1_145 | TTAGACGCGAGGCAATGTAGAATATGCGATAAAAACTAGTT | 41 | | NA | | NA | | | NA | |
|  | Spacer_NoG1_146 | AGCTGTTACGACAACGCTATTGAGGAGATGGATTGT | 36 | | NA | | NA | | | NA | |
|  | Spacer_NoG1_147 | TGGCGATCGATTTCGTCAAAGTAGCGATCGTATTCGTCTTGGCT | 44 | | 22 | | *Haloterrigena* *turkmenica* DSM 5511 plasmid pHTUR01 | | | plasmid | |
|  | Spacer_NoG1_148 | CTCACGCCAGCGATTGGGAGGCGGGGGGGACGATC | 35 | | NA | | NA | | | NA | |
|  | Spacer_NoG1_149 | CGGGTACGACTTCGGTTCGTATTCGTAGTCGTCGGG | 36 | | NA | | NA | | | NA | |
|  | Spacer_NoG1_150 | AATAGAGGGTTCTATACGACCATTAGCTACTTATAGA | 37 | | NA | | NA | | | NA | |
|  | Spacer_NoG1_151 | ATATTGACGCCCCTTAGAAATATTGGGGTTTTCAAGTCT | 39 | | NA | | NA | | | NA | |
|  | Spacer_NoG1_152 | GAGCTAGTCGGTAAATCATCTAAACCACACATCAG | 35 | | NA | | NA | | | NA | |
|  | Spacer_NoG1_153 | ATATAGAAGAGGGTAAAAAGAAGGCTAGAAGGTTAGGT | 38 | | 20 | | JQ340389_JQ340389 *Vibrio* phage pVp-1 | | | phage | |
|  | Spacer_NoG1_154 | CTCTTAAGGTTAAATGAATCTACACTGAGGGAGGTTTT | 38 | | NA | | NA | | | NA | |
|  | Spacer_NoG1_155 | CTCAGCCAATCTTAGTGTCTCAGCTCGTTTAGATTCA | 37 | | NA | | NA | | | NA | |
|  | Spacer_NoG1_156 | ATATTTTTACATCACATCCACTCGAACATATTCTC | 35 | | NA | | NA | | | NA | |
|  | Spacer_NoG1_157 | TGGAAAATAGATGAAATGGATTGATTCCTCCTGG | 34 | | NA | | NA | | | NA | |
|  | Spacer_NoG1_158 | CTTAGATCAGTTGAGGGGGTATGTTATAGCCTCAG | 35 | | NA | | NA | | | NA | |
|  | Spacer_NoG1_159 | GAAGAATCAATGGCAGATTCCACGATATGACTTTGG | 36 | | NA | | NA | | | NA | |
|  | Spacer_NoG1_160 | GACCTGAATCTAGATCTTCAAAATTGCTAGACCACACA | 38 | | NA | | NA | | | NA | |
|  | Spacer_NoG1_161 | AAGCACCGATCCTATGCACGGTAAAGTTACCAGGGG | 36 | | NA | | NA | | | NA | |
|  | Spacer_NoG1_162 | GCGGCTATAAAGGAGGTTCGTCCGAACCTCCTTTATT | 37 | | NA | | NA | | | NA | |
|  | Spacer_NoG1_163 | TTATTGTTTTCTATCTTATAAAACTACTTGATCTAA | 36 | | 20 | | *Ralstonia* *solanacearum* CMR15 plasmid CMR15_mp | | | plasmid | |
|  | Spacer_NoG1_164 | ATGTCGTAGCATTAGCCAAATTAGACGCTTCTAA | 34 | | NA | | NA | | | NA | |
|  | Spacer_NoG1_165 | CTCAATATTAGTGTAATTGCAATCACGCACTCATT | 35 | | NA | | NA | | | NA | |
|  | Spacer_NoG1_166 | TCGCCAACAGCGCCGGAGGGATGCCGTTCTTCTCG | 35 | | 21 | | *Sinorhizobium* *fredii* NGR234 plasmid pNGR234b | | | plasmid | |
|  | Spacer_NoG1_167 | TATGCGGTATCGTTGGTGTCGTTGACTTCTATGGC | 35 | | NA | | NA | | | NA | |
|  | Spacer_NoG1_168 | GAGGGACTAGATCCTCTACATGGTGAGGTCGTTTTGGG | 38 | | NA | | NA | | | NA | |
|  | Spacer_NoG1_169 | AAGTTTAACACCAACAGGTAGTAAAAAAGGTATTGAAA | 38 | | 20 | | *Bacillus* *cereus* strain CMCC P0021 plasmid pRML04 | | | plasmid | |
|  | Spacer_NoG1_170 | ATTAACAGAATCTTTCATTTCCTCAGTTACATTTCCACCTTGT | 43 | | 21 | | *Campylobacter* iguaniorum strain 1485E plasmid pCIG1485E | | | plasmid | |
|  | Spacer_NoG1_171 | TCTTAATTCTATAGAGATTTGTTAGCCAATGCCAAAAAATT | 41 | | NA | | NA | | | NA | |
|  | Spacer_NoG1_172 | GATTGAATTAATCGAAAAAAGCAAGGATTTGGGG | 34 | | 22 | | DQ092789 *Enterovirus* E isolate Jena 3802 polyprotein gene, partial cds | | | virus | |
|  | Spacer_NoG1_173 | TTTTCTCTGTATCTAAGTCAGAAGTCGTAAAAGTTGAAAAA | 41 | | NA | | NA | | | NA | |
|  | Spacer_NoG1_174 | TAGGTAAATTTTCATTTTTTTTCTTCCATTGACTAGATTCAATAT | 45 | | 19 | | *Cyanothece* sp. PCC 8801 plasmid pP880101 | | | plasmid | |
|  | Spacer_NoG1_175 | CCTGAAAATATTACGGCCGTTGCTACAGGTAAATC | 35 | | NA | | NA | | | NA | |
|  | Spacer_NoG1_176 | CTGAGGAAGATATTTATAATGCCATAGACAAGTGGAACGA | 40 | | 20 | | KT968831 *Pseudomonas* phage YMC1102R656 | | | phage | |
|  | Spacer_NoG1_177 | GTGGAAAAGCAAGTACCGGGGTCAGTGGAGATCA | 34 | | NA | | NA | | | NA | |
|  | Spacer_NoG1_178 | GAGAATAGGACTCATGCGTCCTTTAGCCAATTGAGGACTAA | 41 | | NA | | NA | | | NA | |
|  | Spacer_NoG1_179 | CTGAAGACAAATTAGATAATATTAGTCAGACAAA | 34 | | 20 | | *Cronobacter* *turicensis* z3032 plasmid pCTU1 | | | plasmid | |
|  | Spacer_NoG1_180 | GATACGGTGACAAACTTGGCGGCGGCTACCGGGGGAACC | 39 | | NA | | NA | | | NA | |
|  | Spacer_NoG1_181 | CTGTGAAGCATCCTAGCCATCATGGAAACCTGTTAGCAGA | 40 | | NA | | NA | | | NA | |
|  | Spacer_NoG1_182 | GGTGGCGTCCTTCCCATCCCTGTAAGGCTGTAGGGTG | 37 | | NA | | NA | | | NA | |
|  | Spacer_NoG1_183 | TTGCCGGATTGTCCGCCCCTCTACCCGCGCTCGT | 34 | | NA | | NA | | | NA | |
|  | Spacer_NoG1_184 | TTAAAATCTATACCCAAAAACTTAACTATGGATCAGGTAGT | 41 | | 19 | | *Anabaena* *cylindrica* PCC 7122 plasmid pANACY.04 | | | plasmid | |
|  | Spacer_NoG1_185 | TTTCTGCCGCCCTCGATGCCGAACTGGAGATCCTCGAAG | 39 | | NA | | NA | | | NA | |
|  | Spacer_NoG1_186 | TTTAGAATTTACCGAAGCTGATTACAAAGAGTTAAGAGAA | 40 | | 20 | | *Jeotgalibacillus* sp. D5 plasmid | | | plasmid | |
|  | Spacer_NoG1_187 | GGAAAAATGTGTAACTATTTCCCACAAATGGCTGA | 35 | | NA | | NA | | | NA | |
|  | Spacer_NoG1_188 | TGCCAGGAGAGGATCTCCTCTCCGTTTTTAAAGATAG | 37 | | NA | | NA | | | NA | |
|  | Spacer_NoG1_189 | GGGAAATATCCCTCTGTCCACAAGATCCCGCAC | 33 | | NA | | NA | | | NA | |
|  | Spacer_NoG1_190 | AGTAGCTTTCGCGTCGGATGGTGCCGAAGCGATCG | 35 | | NA | | NA | | | NA | |
|  | Spacer_NoG1_191 | ATGTTTTGTTATCCGAGTCATAATCAAACAACATAG | 36 | | 20 | | *Rhizobium* sp. N541 plasmid pRspN541e | | | plasmid | |
|  | Spacer_NoG1_192 | AGGAAGAAGTCGAAACGCTGGGGCGACTAATTCAC | 35 | | NA | | NA | | | NA | |
|  | Spacer_NoG1_193 | GTACACCAACCCCGGATCGTCGGCGAACAGCAAG | 34 | | NA | | NA | | | NA | |
|  | Spacer_NoG1_194 | TTTCTCTATTTTTAATTTTTAGTTAACGGTTGAATAG | 37 | | 21 | | KU556803 *Faustovirus* strain D3 | | | virus | |
|  | Spacer_NoG1_195 | CCCAGGGGTTCGAGTCGCCATCGAAAATCATGAATATT | 38 | | NA | | NA | | | NA | |
|  | Spacer_NoG1_196 | GCCGGAAAGGTTTTTGGCGGTGGCACATCTAGCAATGA | 38 | | NA | | NA | | | NA | |
|  | Spacer_NoG1_197 | GGAACGAAAGCGTACCAACAGGCGCGCGAGGAATTC | 36 | | NA | | NA | | | NA | |
|  | Spacer_NoG1_198 | TTAGACTTCCAAAAAAAAGTCTAACAAAAAGTCT | 34 | | 20 | | *Borrelia* *valaisiana* VS116 plasmid VS116_cp32-2-7 | | | plasmid | |
|  | Spacer_NoG1_199 | AGCTTTCTCGCGACCTGGGCGCAAGTCAACCCTCG | 35 | | NA | | NA | | | NA | |
|  | Spacer_NoG1_200 | ATTAATCGCGATGTCCGTATATTGGCGTGAGTATTCGC | 38 | | NA | | NA | | | NA | |
|  | Spacer_NoG1_201 | AATATGGTCAGCTCGACCATATCTCCCGTGTCATG | 35 | | NA | | NA | | | NA | |
|  | Spacer_NoG1_202 | AATAGGAATCCCTCCACTCTCTGATGAGGAATGGGAT | 37 | | 20 | | VSVGLYPW_M35229 Vesicular stomatitis Indiana virus strain 85-GM-B glycoprotein gene | | | virus | |
|  | Spacer_NoG1_203 | CCTACTGAGACACAATTAGAATTCTTATACTCTAAATTCTCA | 42 | | NA | | NA | | | NA | |
|  | Spacer_NoG1_204 | TTTAGATCTAAATCGTAATAAGGAGAGGGCTGAA | 34 | | NA | | NA | | | NA | |
|  | Spacer_NoG1_205 | CATTTTTCTTTCTCCCATCGATTAGAATAAATAT | 34 | | NA | | NA | | | NA | |
|  | Spacer_NoG1_206 | TAGTTACCAGGGGGGCAGAATCCTACTCGTGGAA | 34 | | NA | | NA | | | NA | |
|  | Spacer_NoG1_207 | CTGTGCAAGGTAAATTTGCATGAGGATCTCCTTCACA | 37 | | NA | | NA | | | NA | |
|  | Spacer_NoG1_208 | TAGAGCTACCATGGTGTTTATGTCTCCTATTTAGACTCTG | 40 | | NA | | NA | | | NA | |
|  | Spacer_NoG1_209 | ACCATTATGAGGAGGGTTCCTCATATGAACTCAAG | 35 | | NA | | NA | | | NA | |
|  | Spacer_NoG1_210 | ACATTCAAAATCATCATAGGTTTGATTTTTAATC | 34 | | NA | | NA | | | NA | |
|  | Spacer_NoG1_211 | CTCAAAATTAACCGATAATCCAAAGCTTATAGAA | 34 | | NA | | NA | | | NA | |
|  | Spacer_NoG1_212 | ACTAGTCCGCCAATATTCTATAATGCAGTAGTTTTC | 36 | | NA | | NA | | | NA | |
|  | Spacer_NoG1_213 | CCCAGTAGCCATCGGGGTTCGGTGTTAGAGGTACAA | 36 | | NA | | NA | | | NA | |
|  | Spacer_NoG1_214 | TAGTGTGACTTCTTCTTCGTTACCACACTGATGGGGTATATTT | 43 | | NA | | NA | | | NA | |
|  | Spacer_NoG1_215 | TTTACCTTGGGGAAACGACAATATGATGACCCCAGAA | 37 | | NA | | NA | | | NA | |
|  | Spacer_NoG1_216 | CTTCACCCTCTCCCGCAGTCGCGAAATCTAATCGCTTCCCTAAAGCTTCAA | 51 | | NA | | NA | | | NA | |
|  | Spacer_NoG1_217 | TTTAAAGATTCTTCTATTTCTGCTTTTATTCTCT | 34 | | 20 | | *Ornithobacterium* *rhinotracheale* plasmid pOR1 | | | plasmid | |
|  | Spacer_NoG1_218 | TTGGAAGCCCCGTCGCTTTAGCGCGGGGATTGGTTACACAACTTGTCAGATA | 52 | | NA | | NA | | | NA | |
|  | Spacer_NoG1_219 | TGTAAACCCAGTTGTGCCAAAATTGGTTCCG | 31 | | NA | | NA | | | NA | |
|  | Spacer_NoG1_220 | ATTCTGGCGGATTGACATAGGCATTGCTACTGCAC | 35 | | NA | | NA | | | NA | |
|  | Spacer_NoG1_221 | CATTGCCCAAAGCGTTCGGCGTCTGTTCGGCG | 32 | | NA | | NA | | | NA | |
|  | Spacer_NoG1_222 | GAGGGTGTGGAGAGTCACGCTGAGCGCCTCGTCGAT | 36 | | 22 | | DQ184476 Orf virus strain NZ2 | | | virus | |
|  | Spacer_NoG1_223 | TGGAGGGGAGGTGGTAGTACCTCCCCGGTTGTGTTA | 36 | | NA | | NA | | | NA | |
|  | Spacer_NoG1_224 | GCTACTCGTCCTCGCCGTCTTCGAGCCACAACTC | 34 | | NA | | NA | | | NA | |
|  | Spacer_NoG1_225 | TCCAGATTTTGGGCGATACTCGGAGGAAGTAGGCGCG | 37 | | NA | | NA | | | NA | |
|  | Spacer_NoG1_226 | TGTATAATGGATACAAACAGACACAGCAGAACCT | 34 | | NA | | NA | | | NA | |
|  | Spacer_NoG1_227 | CTTATTATCAAAATTAGAATCGGGGCAAAGAAATCT | 36 | | NA | | NA | | | NA | |
|  | Spacer_NoG1_228 | GTAAGATAAGATGCCCGAAATCACAAATCCTCGCGCTGT | 39 | | NA | | NA | | | NA | |
|  | Spacer_NoG1_229 | CGGCGTTCCGATCGAAGGCGATCGTCGTTGCATCGA | 36 | | NA | | NA | | | NA | |
|  | Spacer_NoG1_230 | GCATTCGTTGCAAGGTTCTCCATCAATTGGGTTCA | 35 | | NA | | NA | | | NA | |
|  | Spacer_NoG1_231 | ATTCATTTAATTAAATCGTAATACCTATAGAAAAT | 35 | | NA | | NA | | | NA | |
|  | Spacer_NoG1_232 | ACTCCTTTACTTAAGAGGTAATCTGATAATTGGT | 34 | | NA | | NA | | | NA | |
|  | Spacer_NoG1_233 | CTGAGTTGCCGTAATTGAATTTGTGTGCCGATTATAAGT | 39 | | NA | | NA | | | NA | |
|  | Spacer_NoG1_234 | TTGTGGCATTCGTTGCAAGGTTCTCCATCAATTGGGTTCA | 40 | | NA | | NA | | | NA | |
|  | Spacer_NoG1_235 | ATTATTGGGACGATACAGATTGGATCTAATTTTTG | 35 | | NA | | NA | | | NA | |
|  | Spacer_NoG1_236 | TAAAGAGGATCTATATTGGGTACAGAAAGAAAAAG | 35 | | NA | | NA | | | NA | |
|  | Spacer_NoG1_237 | GTTAAATTTTTCGGTTTTGAATGTGGCATGAAGGA | 35 | | NA | | NA | | | NA | |
|  | Spacer_NoG1_238 | TTTACGCCAAGGACGTAAACTAGTTTTAGCCGCT | 34 | | NA | | NA | | | NA | |
|  | Spacer_NoG1_239 | CGGCGTCAGGCATATGTACCAGTGTCTGTGGATTGAAC | 38 | | NA | | NA | | | NA | |
|  | Spacer_NoG1_240 | TCGGACACAGATTAAAGAACATGAGGCCTCAATG | 34 | | NA | | NA | | | NA | |
|  | Spacer_NoG1_241 | GGGCTTGGCTAGCATCCTCGGGAGTTGTCGTCACTCAG | 38 | | NA | | NA | | | NA | |
|  | Spacer_NoG1_242 | TCGGCTCCGTCAAGTTAGCCCGATCGCGCTTCGACAT | 37 | | NA | | NA | | | NA | |
|  | Spacer_NoG1_243 | TTTGGCAAAGGCATCCCGCGCCTCTTGAGTGTTGA | 35 | | NA | | NA | | | NA | |
|  | Spacer_NoG1_244 | TTGTCAGTGTTCCACCCGGATAAGTATAAACTAA | 34 | | NA | | NA | | | NA | |
|  | Spacer_NoG1_245 | TTTAAAAGGCTTTGGTGGTGTTAGTAACCCCGTTGGTCTAA | 41 | | 19 | | AB626962 *Staphylococcus* phage S24-1 DNA | | | phage | |
|  | Spacer_NoG1_246 | ATTTTTGCAAGAGTGCCTTCCTGTCTTAGTCCTC | 34 | | NA | | NA | | | NA | |
|  | Spacer_NoG1_247 | GCTGATTTAATTTAGGCTTTAGATCGTCTAATTCGATTTCAA | 42 | | NA | | NA | | | NA | |
|  | Spacer_NoG1_248 | GGTTCCGCCCATGCCTTTGGCGTACTCCGTCCAGTAAGG | 39 | | NA | | NA | | | NA | |
|  | Spacer_NoG1_249 | ATTAGATCCCGGTAGGCCAGCTACAAAAGTACTGA | 35 | | NA | | NA | | | NA | |
|  | Spacer_NoG1_250 | ATAGTAGGGTTAGGATCCACTTACTATGGCTTCC | 34 | | NA | | NA | | | NA | |
|  | Spacer_NoG1_251 | GAAGAAAAATTCTTTAGTTTTTACTTTAGGTTCCTTAG | 38 | | NA | | NA | | | NA | |
|  | Spacer_NoG1_252 | GTGGTGATTTTCGGGTGGATCTTTCACCCTATCGAG | 36 | | NA | | NA | | | NA | |
|  | Spacer_NoG1_253 | TTGTCGTACCTTCAGATAATTGTGATTGACGACTTTTGAC | 40 | | NA | | NA | | | NA | |
|  | Spacer_NoG1_254 | CATATATTTTAAATATCGACACTTTACTTTTGAGTTAG | 38 | | NA | | NA | | | NA | |
|  | Spacer_NoG1_255 | GCTATGAGCCGGACATCCAGCCTGGTGTACCTGATGA | 37 | | NA | | NA | | | NA | |
|  | Spacer_NoG1_256 | CCCAATTCTACTAATTCTCTAATAGAAGCACTTTT | 35 | | NA | | NA | | | NA | |
|  | Spacer_NoG1_257 | ATATCAGGTTCCCCCTCCACGGTCTACTCTTGCCC | 35 | | NA | | NA | | | NA | |
|  | Spacer_NoG1_258 | GATAGCCCAATTCTACTAATTCTCTAATAGAAGCACTTTT | 40 | | NA | | NA | | | NA | |
|  | Spacer_NoG1_259 | TTATTGGATTAGGTAGTACAAAAGTAGCTAGAAG | 34 | | NA | | NA | | | NA | |
|  | Spacer_NoG1_260 | AATAGTAGCCATATAAAACTATAAAATAAAAATTAC | 36 | | 20 | | *Helicobacter* *cetorum* MIT 00-7128 plasmid pHCW | | | plasmid | |
| 2 | Spacer_NoG2_01 | CTTTTTAAATTAGGTCAGCAAGCCTCTGACCTC | 33 | | NA | | NA | | | NA | |
|  | Spacer_NoG2_02 | ATCACTGGAGACTTCTCCCTCTATTGGGTTCCTA | 34 | | NA | | NA | | | NA | |
|  | Spacer_NoG2_03 | AAAAGATTTTTAGATGAGGAATAGTCAGCGAGTTGATCTGCTGGAGC | 47 | | NA | | NA | | | NA | |
|  | Spacer_NoG2_04 | ATCTTCATACCAATTAATATAAACATTTAGGATACG | 36 | | NA | | NA | | | NA | |
|  | Spacer_NoG2_05 | GCAGTTTCTTATGGAAGAAAGGGTGATTCAATCTATC | 37 | | NA | | NA | | | NA | |
|  | Spacer_NoG2_06 | ATTTGAGAAGCTATGTCAGGGATTTCGACATCTAATT | 37 | | NA | | NA | | | NA | |
|  | Spacer_NoG2_07 | CTGCAACCTAGAAAAATAAATGTCTTATTTAGAA | 34 | | NA | | NA | | | NA | |
|  | Spacer_NoG2_08 | TTCGCCGGGAGAGCCGTTGGCGGTGTGGCGGACGAA | 36 | | NA | | NA | | | NA | |
|  | Spacer_NoG2_09 | GACCGACCGACAGGCGATCGCGATTCAACTCAGC | 34 | | NA | | NA | | | NA | |
|  | Spacer_NoG2_10 | TTGTGTTTTTACTTTTGTTAGATTAGGGTTTATAACTC | 38 | | NA | | NA | | | NA | |
|  | Spacer_NoG2_11 | TGGACATCTCGGCTTTAAGGTAATCATTGACTCTT | 35 | | NA | | NA | | | NA | |
|  | Spacer_NoG2_12 | TGCAAGATAAAGGACATTAAAGATCCTCAAAACCGC | 36 | | NA | | NA | | | NA | |
|  | Spacer_NoG2_13 | GAAAAACCGTGGTTCAATCATCACTTTCCTCTTCTT | 36 | | NA | | NA | | | NA | |
|  | Spacer_NoG2_14 | TGTGCTATGACATGTGCTATCTTTTTCTTACCCTTGAAAAGCTGATT | 47 | | NA | | NA | | | NA | |
|  | Spacer_NoG2_15 | TGTGCTATGACACCACTTCTCCTTA | 25 | | NA | | NA | | | NA | |
|  | Spacer_NoG2_16 | GTGAATAATAAAATATCATCCCATCAATGGTATCAC | 36 | | NA | | NA | | | NA | |
|  | Spacer_NoG2_17 | AGTGTAATTACCGGGGGCGTTAGTAAGTACTGCCCCCT | 38 | | NA | | NA | | | NA | |
|  | Spacer_NoG2_18 | ATGTGGTTGCAGTCTCCCATGATTCCAACTACAAA | 35 | | NA | | NA | | | NA | |
|  | Spacer_NoG2_19 | AAAAATGTAATTATCAGTGACGCTACGGCTAAAG | 34 | | NA | | NA | | | NA | |
|  | Spacer_NoG2_20 | ACCCGGTTAGGTTGTGTCAAACTATGCTTGTCACAA | 36 | | NA | | NA | | | NA | |
|  | Spacer_NoG2_21 | ATTACAACAGGTAGAGGATGGCCTGAGGGGGGATACGG | 38 | | NA | | NA | | | NA | |
|  | Spacer_NoG2_22 | TTCTAAATCAATCCCACTTGTACGCCAATCCATGA | 35 | | NA | | NA | | | NA | |
|  | Spacer_NoG2_23 | GGCAAACAACCTGTCAATACAGGGGAGCGACGCAAGGTAAG | 41 | | NA | | NA | | | NA | |
|  | Spacer_NoG2_24 | TAGCATAATTTCTACTAACATATACATAAGAAC | 33 | | NA | | NA | | | NA | |
|  | Spacer_NoG2_25 | CTCTAGGCGGATAATGCACATCAGAGATCAAATCCC | 36 | | NA | | NA | | | NA | |
|  | Spacer_NoG2_26 | CTCAGGAGATGGGGGCTCAGCCTGTGTATTTGA | 33 | | NA | | NA | | | NA | |
|  | Spacer_NoG2_27 | CTTCCCTTTGTTTCTATACCCAATATAGATCGGT | 34 | | NA | | NA | | | NA | |
|  | Spacer_NoG2_28 | GCTCTACCTTAGAATCAAAAACTAATCCTTGTGAGGAAGA | 40 | | NA | | NA | | | NA | |
|  | Spacer_NoG2_29 | TTAGGTAATATTAAGAAGCTCTGCCATCATTGGAAT | 36 | | NA | | NA | | | NA | |
|  | Spacer_NoG2_30 | TTTAGTTGCATCCATTTTTAGTCCTTCCCATTTA | 34 | | NA | | NA | | | NA | |
|  | Spacer_NoG2_31 | CCCACTTCCCGGTATTAAGGAGGGCATTCTTTACTTC | 37 | | NA | | NA | | | NA | |
|  | Spacer_NoG2_32 | TGGCAAGATTTCGATCGGGGATACACTCGGAACCTC | 36 | | NA | | NA | | | NA | |
|  | Spacer_NoG2_33 | TACAAATACTATAAGACTGTGTTACATTATATGTGT | 36 | | NA | | NA | | | NA | |
|  | Spacer_NoG2_34 | ACTACAGTGGTAGGCAATTAAATATCATGGTTAAA | 35 | | NA | | NA | | | NA | |
|  | Spacer_NoG2_35 | AACGCCTTTTGGAAGGCGGCGGCAAAAGTAAACGAGT | 37 | | NA | | NA | | | NA | |
|  | Spacer_NoG2_36 | AAACCTTGGAAGGCTACACGATAACTTAAACCTTTA | 36 | | NA | | NA | | | NA | |
|  | Spacer_NoG2_37 | GTTAGACAGCTTGTCCTTGTTTCTTGTTTTTGCCC | 35 | | NA | | NA | | | NA | |
|  | Spacer_NoG2_38 | GAAAGATGCTCCAACTGTTCCTACTGACATCCCGCCT | 37 | | NA | | NA | | | NA | |
|  | Spacer_NoG2_39 | TTATAGTTATAAAAACTTATCTAACCCATAGGATAAA | 37 | | NA | | NA | | | NA | |
|  | Spacer_NoG2_40 | CTCGGATTTCACCGTTGTCTTTAAGTTTCACGAAACT | 37 | | NA | | NA | | | NA | |
|  | Spacer_NoG2_41 | ACGTCAAGCATCACCATGTATTTCTTCTTACTGCTA | 36 | | NA | | NA | | | NA | |
|  | Spacer_NoG2_42 | ATATTCCTAAAGGATACATAAAATTAAACTATAAA | 35 | | NA | | NA | | | NA | |
|  | Spacer_NoG2_43 | TAGTAGCTACGAAGATTTAGATTCAGCTTATGAAA | 35 | | NA | | NA | | | NA | |
|  | Spacer_NoG2_44 | TGTTGCCTGATAATCTTCAATAGCCTTTTTAATCCT | 36 | | NA | | NA | | | NA | |
|  | Spacer_NoG2_45 | CTTTTTCTGTTTCACCTTCCACCCATCTTTTCCT | 34 | | NA | | NA | | | NA | |
|  | Spacer_NoG2_46 | GAGCCATAGCGTGGCTGTGGGCGACTCCATTGAAA | 35 | | NA | | NA | | | NA | |
|  | Spacer_NoG2_47 | CCTTTATTTTGGTTCTAGAAATAATACCTATTGCATTAGT | 40 | | NA | | NA | | | NA | |
|  | Spacer_NoG2_48 | TCATTTTCTGGAATTAAGTACATATTGGATTACT | 34 | | NA | | NA | | | NA | |
|  | Spacer_NoG2_49 | ATGTATTTAATAAGTGTGGCTAATAGGTTAAATAAA | 36 | | NA | | NA | | | NA | |
|  | Spacer_NoG2_50 | TCAATAGTTGGATCGTAACTGTAATCTATTCTATAGT | 37 | | NA | | NA | | | NA | |
|  | Spacer_NoG2_51 | ACGGGAAGTTGCATTTTTTCCGCCCATTCGTTTAC | 35 | | NA | | NA | | | NA | |
|  | Spacer_NoG2_52 | ATGATGAGGTGGATTTTCAATCATTAGATGGTGCGGGTT | 39 | | NA | | NA | | | NA | |
|  | Spacer_NoG2_53 | CTGACGGGGTAGCTAACAGGTTATTCAATGCAAA | 34 | | NA | | NA | | | NA | |
|  | Spacer_NoG2_54 | ATTATTGCCCTATTTCTAGGTAAAATATCTTGTAGA | 36 | | NA | | NA | | | NA | |
|  | Spacer_NoG2_55 | CAATAGACTAATTATTGTTATTGTAACATTGACAA | 35 | | NA | | NA | | | NA | |
|  | Spacer_NoG2_56 | GGCATCGTCAGCTACTACTTCTCTCCAAAGGTATC | 35 | | NA | | NA | | | NA | |
|  | Spacer_NoG2_57 | CTCAAGACCTAGTATAGTTAGATTAACCGATTCAGT | 36 | | NA | | NA | | | NA | |
|  | Spacer_NoG2_58 | ACTATCCCCAAGCATCCGAAATACAGAGAGCATA | 34 | | NA | | NA | | | NA | |
|  | Spacer_NoG2_59 | AGCTGTGATAGTATAATCTTCACCCAGAGGATACCAA | 37 | | NA | | NA | | | NA | |
|  | Spacer_NoG2_60 | TGAATCAAGTTGGAATTCTTGCCAAAACCCTGTATA | 36 | | NA | | NA | | | NA | |
|  | Spacer_NoG2_61 | CTAGATTTACGAATGCTTCCTCTTGGTAGACGT | 33 | | NA | | NA | | | NA | |
|  | Spacer_NoG2_62 | TTTGCATCGCGAAGTTAAGAGATACTCTGACGAT | 34 | | NA | | NA | | | NA | |
|  | Spacer_NoG2_63 | CTCTTAGAGTTAATAGATACTGATGAAAAATTGG | 34 | | NA | | NA | | | NA | |
|  | Spacer_NoG2_64 | CTGGTGGAGGTTGCGGATAGTCGATATCAATCCCT | 35 | | NA | | NA | | | NA | |
|  | Spacer_NoG2_65 | ATAAGCGATCATGTCCTCTAAGCTACTGTAAATGGCTT | 38 | | NA | | NA | | | NA | |
|  | Spacer_NoG2_66 | TTGAGTCAATTCCTTAAGAAAGTTCAAACACCTGGAAC | 38 | | NA | | NA | | | NA | |
|  | Spacer_NoG2_67 | TATAAAGATGATAGATACTTTAATGATGTCTTAAA | 35 | | NA | | NA | | | NA | |
|  | Spacer_NoG2_68 | ATCGACGATGTCGATCCGTGGACTTTGGAAGTGTCGGT | 38 | | NA | | NA | | | NA | |
|  | Spacer_NoG2_69 | CTCTCTCGCCACCACACTTGGGGACGCTTACATCGTTCCTCAA | 43 | | NA | | NA | | | NA | |
|  | Spacer_NoG2_70 | CCTTCAATTGACGAAGACCCCTCCCAACAGACCCC | 35 | | NA | | NA | | | NA | |
|  | Spacer_NoG2_71 | CGATACTTTTTCGAGGTTCTCCCCGAAAGCGTTG | 34 | | NA | | NA | | | NA | |
|  | Spacer_NoG2_72 | ATGTAAAGTTGGGTTGACACCCTGATGAGGGCTTGA | 36 | | NA | | NA | | | NA | |
|  | Spacer_NoG2_73 | TTTGTATCCAAAATAGATCGGTATAGGGAGGTTGTCAA | 38 | | NA | | NA | | | NA | |
|  | Spacer_NoG2_74 | ATCTATGTCCATTTTTTGTACCCGTCTCCTTTGTTTA | 37 | | NA | | NA | | | NA | |
|  | Spacer_NoG2_75 | AAGTTAGAAATACTTTATGGTTCTTTGACTTAAAC | 35 | | NA | | NA | | | NA | |
|  | Spacer_NoG2_76 | GTGATGATTTTCTTAGTCATGGTTGTTTCCTTTTCTT | 37 | | NA | | NA | | | NA | |
|  | Spacer_NoG2_77 | AAACATCTGTTTAACAAAAATTTAATATTAATCAT | 35 | | NA | | NA | | | NA | |
|  | Spacer_NoG2_78 | ATGGATAGATAAGATAAACTCTTCATCCATATTT | 34 | | NA | | NA | | | NA | |
|  | Spacer_NoG2_79 | TGGCGGTAAGTATTTCGATTCTTTTCTTAGGAGTAA | 36 | | NA | | NA | | | NA | |
|  | Spacer_NoG2_80 | TCCCACAGGTTCCGGCAAGACGTTTATGTTTTGCCAAA | 38 | | NA | | NA | | | NA | |
|  | Spacer_NoG2_81 | ATTCGTGAATCTCAGATTTACTATAGCAAGTATAAGGAA | 39 | | NA | | NA | | | NA | |
|  | Spacer_NoG2_82 | TTATATGGCTACTATTGGACACAAATCAGAATTGT | 35 | | NA | | NA | | | NA | |
|  | Spacer_NoG2_83 | CCCGTCTCTTCACGGTGACAGATTGGGTCTAGA | 33 | | NA | | NA | | | NA | |
|  | Spacer_NoG2_84 | GGGTCTGGTCTCCTCTACCCCGATGACTGATTAAAT | 36 | | NA | | NA | | | NA | |
|  | Spacer_NoG2_85 | ACCTTCCCCTACATCTGGGATATCTTCTAATAGT | 34 | | NA | | NA | | | NA | |
|  | Spacer_NoG2_86 | AGAAAAGGAAAAATACTCACCTACTTCTAAATCCTC | 36 | | NA | | NA | | | NA | |
|  | Spacer_NoG2_87 | AACAATTAGAATCATGGCGACTGCGATACTCATA | 34 | | NA | | NA | | | NA | |
|  | Spacer_NoG2_88 | GTGGTGTCGTCGTCGTGGTTTCCGGTGTGTTTCCA | 35 | | NA | | NA | | | NA | |
|  | Spacer_NoG2_89 | CGATCACCCTATCGCTCAAGGGCGGTGTGGTGT | 33 | | NA | | NA | | | NA | |
|  | Spacer_NoG2_90 | AATAAGCACGAAGTCGCCATCGGGGAGTCGGGTAACAA | 38 | | NA | | NA | | | NA | |
|  | Spacer_NoG2_91 | TTAAATAACCGTAAGCGCGCCAGCACTCACGATAAG | 36 | | NA | | NA | | | NA | |
|  | Spacer_NoG2_92 | GGCGATCGAGATACGGCAGATTGGCATGATTAGGTCT | 37 | | NA | | NA | | | NA | |
|  | Spacer_NoG2_93 | AGTTCGAGCTTTTACCTTCCGAACTCGCCTAACAACC | 37 | | NA | | NA | | | NA | |
|  | Spacer_NoG2_94 | TCCGATCTTCATCTTAATAGCAATCAGACTCGCGTAATG | 39 | | NA | | NA | | | NA | |
|  | Spacer_NoG2_95 | CGAATTGGGCTGAAGCAAAATAGCGGTTGAAACCC | 35 | | NA | | NA | | | NA | |
|  | Spacer_NoG2_96 | ACAATGAAGGTTTGAAGCCCGGTGGCTGGTGCGGGAG | 37 | | NA | | NA | | | NA | |
|  | Spacer_NoG2_97 | TCCTGCCTATGTAATCGGTACGAAAAGACGCTTAA | 35 | | NA | | NA | | | NA | |
|  | Spacer_NoG2_98 | GGAAACTTTCGGTTAGCCCTCGGTGCTGATACCACCGA | 38 | | NA | | NA | | | NA | |
|  | Spacer_NoG2_99 | TTTTGAAAGGCTTTAACAGTCGCCTTCCCCGAAACA | 36 | | NA | | NA | | | NA | |
|  | Spacer_NoG2_100 | GCTCTGAATTCGCTCCTGCTTTTGGCGAAGACGAGT | 36 | | NA | | NA | | | NA | |
| 3 | Spacer_NoG3_01 | TCTCCTGCACCACAGTAGGGGTGGAACACTGTTGAGC | 37 | | NA | | NA | | | NA | |
|  | Spacer_NoG3_02 | TCTGCGATGTCCGCCCACGGCATTCGCGTAGGAATCTC | 38 | | NA | | NA | | | NA | |
|  | Spacer_NoG3_3 | GCGGACTTTGTGAAGATCTCGATAAAGAGATTCTTCGAAAAA | 42 | | NA | | NA | | | NA | |
|  | Spacer_NoG3_4 | ATCGAGCGCATACCGTCGAGCTGCTCGCCCTCTTC | 35 | | NA | | NA | | | NA | |
|  | Spacer_NoG3_5 | GGGGGATTTCAGGCAAGAGATGTTTTCAAATTCTTGGTTTA | 41 | | NA | | NA | | | NA | |
|  | Spacer_NoG3_6 | AAGAAGTGCTCGCTCAAGAGAGCGATATAGCGCT | 34 | | NA | | NA | | | NA | |
|  | Spacer_NoG3_7 | TTTCTACATTGGGAGCGATCCAGAAAACCCA | 31 | | NA | | NA | | | NA | |
|  | Spacer_NoG3_8 | CCTCATACCTTAGCTGGAAATCGTTAGTCACCGCCGTCCGA | 41 | | NA | | NA | | | NA | |
|  | Spacer_NoG3_9 | CCAACGCCCCGGAAACCGTACGCGGTGCGATCG | 33 | | NA | | NA | | | NA | |
|  | Spacer_NoG3_10 | TTCACTTTAGGGGAAGAGGGTTTAACATCACCTAGATT | 38 | | NA | | NA | | | NA | |
|  | Spacer_NoG3_11 | CTGTCGGCGGTTTTCGGGGATGCCGTTTTTAAGA | 34 | | NA | | NA | | | NA | |
|  | Spacer_NoG3_12 | TGACCAATTTAACGCCCTCGAGAGTGAAGCGTT | 33 | | NA | | NA | | | NA | |
|  | Spacer_NoG3_13 | CACCGCCCGGCGGGGCGCAAACACAGCAACTCCTTC | 36 | | NA | | NA | | | NA | |
|  | Spacer_NoG3_14 | GGTAACAAATCAGGGCGGTGTCGAGGCGGGATTCAA | 36 | | NA | | NA | | | NA | |
|  | Spacer_NoG3_15 | AAGATCGACCCCCCGACGATGAGGAGTCCGCCGATCGCACC | 41 | | NA | | NA | | | NA | |
|  | Spacer_NoG3_16 | GTTCGCCCCTGGCACTTGTTGCGATCGGGCTGTTCTCAAA | 40 | | NA | | NA | | | NA | |
|  | Spacer_NoG3_17 | GCCTTATCGAACGCGGTTTCGATCGGCGGAAGCGTTGCC | 39 | | NA | | NA | | | NA | |
|  | Spacer_NoG3_18 | TTGATGATGGCATCTATTCGGAGAAAGCTGAAAG | 34 | | 20 | | *Rhizobium* sp. N941 plasmid pRspN941a | | | plasmid | |
|  | Spacer_NoG3_19 | TGGCGATCGCTCCCACCGAGTCAATTAGGACA | 32 | | NA | | NA | | | NA | |
|  | Spacer_NoG3_20 | CTGTAACAGTGTAAAGTCCGTTGAAGCCATAAAAA | 35 | | NA | | NA | | | NA | |
|  | Spacer_NoG3_21 | TTAAGGGATTTCGGAGAGCAATTGTTTGCTACTACGTC | 38 | | NA | | NA | | | NA | |
|  | Spacer_NoG3_22 | GTCGGGTGTTGTTGACTCCGATTCAATAACATTG | 34 | | NA | | NA | | | NA | |
|  | Spacer_NoG3_23 | CTACCTTCCTCAAGATTAGAGTCTTCATTTTCCAT | 35 | | NA | | NA | | | NA | |
|  | Spacer_NoG3_24 | AAGATTTATGGAGAAGGGTATAGAATCTTGATTA | 34 | | NA | | NA | | | NA | |
|  | Spacer_NoG3_25 | AGTTTCAGGGCAAATTAATATTAATATTCTACAT | 34 | | NA | | NA | | | NA | |
|  | Spacer_NoG3_26 | ACTGAATAAAGCTAAAGCTAGTATCAAAGAGGCAGA | 36 | | NA | | NA | | | NA | |
|  | Spacer_NoG3_27 | AATTTCTCTTCTGATAAACCTTGTCTAGATCCATACTCCAA | 41 | | NA | | NA | | | NA | |
|  | Spacer_NoG3_28 | AGTGGAAAAGCAAGTTCCGGGGACAATGGATCTCG | 35 | | NA | | NA | | | NA | |
|  | Spacer_NoG3_29 | GATCCGGTTATAGCTTTTTTAGCCTGTTTAGCATCG | 36 | | NA | | NA | | | NA | |
|  | Spacer_NoG3_30 | CTACGACTACCTCTGTTAAAGCGTCGATATCATCATCATT | 40 | | NA | | NA | | | NA | |
|  | Spacer_NoG3_31 | GAGGGGTTTCCGAAAATCGAATTTCTAGGGTTTGAA | 36 | | 22 | | *Bacillus* *thuringiensis* strain KNU-07 plasmid pBTKNU07-01 | | | plasmid | |
|  | Spacer_NoG3_32 | ATATTTGGCAAAAGGGCAACAATGTAATCGATTTA | 35 | | NA | | NA | | | NA | |
|  | Spacer_NoG3_33 | AATGGGTGAATTTTCCCATTTTAGAGATGCAATCTAA | 37 | | NA | | NA | | | NA | |
|  | Spacer_NoG3_34 | CTAGGGCTTTGCAAAAGCCCTCATTCTCTTGTAGGTA | 37 | | NA | | NA | | | NA | |
|  | Spacer_NoG3_35 | TTTATTCGGCTTCCTTCGTAGGAAATGTAGTTTGCCCC | 38 | | NA | | NA | | | NA | |
|  | Spacer_NoG3_36 | GATTTGTAAAGCTAGTTGTCCCGAAATTAGTGCCA | 35 | | NA | | NA | | | NA | |
|  | Spacer_NoG3_37 | GAATTTTAAAGGTAGTAGGCTCCAATGAAATCAC | 34 | | NA | | NA | | | NA | |
|  | Spacer_NoG3_38 | AAATCCCCTCTTGAGAAGCATAATTTAGAATTTT | 34 | | NA | | NA | | | NA | |
|  | Spacer_NoG3_39 | GAGGTAGCACAGCCAGGCGAGCGGCGTCGAGTTGGGATGCCGT | 43 | | NA | | NA | | | NA | |
|  | Spacer_NoG3_40 | GCTTCCCAACTGTCGATCGTCAAACTCGAACAGTTG | 36 | | NA | | NA | | | NA | |
|  | Spacer_NoG3_41 | CTCTAGCAAAGATACTACTCCACTCGAGAAGGAAGT | 36 | | NA | | NA | | | NA | |
|  | Spacer_NoG3_42 | TCCTCAAAGGATTCCCCCTCCACTTCTTTCCTAG | 34 | | NA | | NA | | | NA | |
|  | Spacer_NoG3_43 | GTAATTCTTGCTCAATTGCGGGTTGGAGCAATCG | 34 | | NA | | NA | | | NA | |
|  | Spacer_NoG3_44 | TCTTCTATTAGGCAGCCGTGATCCTTTCCGCAGATAG | 37 | | NA | | NA | | | NA | |
|  | Spacer_NoG3_45 | TATCTGAAATTGAGCAAGGTATTTCCAGACTTAGAGCTA | 39 | | 21 | | KX507046 *Vibrio* phage S4-7 | | | phage | |
|  | Spacer_NoG3_46 | TAGAGGCACTAGAGGCTGAAAAAGCATTAGATAATC | 36 | | NA | | NA | | | NA | |
|  | Spacer_NoG3_47 | TCCTCCGCGATCAATTGAGCAATTTCCCCATTCGA | 35 | | NA | | NA | | | NA | |
|  | Spacer_NoG3_48 | CGTAGTTGTTTGGGGGAAAGAGTCCTACCAGTGGA | 35 | | NA | | NA | | | NA | |
|  | Spacer_NoG3_49 | CTTCGAAGAGTATCCCCCTTTCTTAAGGGATACCCTTTTGG | 41 | | NA | | NA | | | NA | |
|  | Spacer_NoG3_50 | TCAAATGTTACTTTAAGTGCAATTCCAACATATGG | 35 | | NA | | NA | | | NA | |
|  | Spacer_NoG3_51 | CTGGTGTACCCTCTGATACACCAGGAAGTGGGAGCCTCTTCCTAG | 45 | | NA | | NA | | | NA | |
|  | Spacer_NoG3_52 | TTATTGCCCTGTCTATAGACATTAGGTACTACGCT | 35 | | NA | | NA | | | NA | |
|  | Spacer_NoG3_53 | CTAGAGAATTGAGAAATTTTACGACATTTCCTTCTCT | 37 | | NA | | NA | | | NA | |
|  | Spacer_NoG3_54 | TACCCCCTTTGATTCGGAAAGACTCATTGTCTTTC | 35 | | NA | | NA | | | NA | |
|  | Spacer_NoG3_55 | ATAGTTATATTGGTAGCTGTAGTCACTTATGCCAT | 35 | | NA | | NA | | | NA | |
|  | Spacer_NoG3_56 | CGTACCCAATACTAGTAATAATATGGCTAGGCAAA | 35 | | NA | | NA | | | NA | |
|  | Spacer_NoG3_57 | CTAATTTTTTGTCCTCTTCCGGCCAACTTGGTGGTGTAA | 39 | | NA | | NA | | | NA | |
|  | Spacer_NoG3_58 | TTCGCCAGAATAAAAATGATGATTACGGTCGTATCCT | 37 | | NA | | NA | | | NA | |
|  | Spacer_NoG3_59 | TCGTCAATGTAGACGACATTACCTCCGTATTAGA | 34 | | NA | | NA | | | NA | |
|  | Spacer_NoG3_60 | TCTATGGGTGTTGGCGCAAATGGTGCTTTAATTTTCTCC | 39 | | NA | | NA | | | NA | |
|  | Spacer_NoG3_61 | TAGATTCTTGCCTAGTCAGTACACAAATATAGTCTACG | 38 | | NA | | NA | | | NA | |
|  | Spacer_NoG3_62 | GATTTGTGATTATATGTGATGTCCTCTTATAGAA | 34 | | NA | | NA | | | NA | |
|  | Spacer_NoG3_63 | ACCTTAGATAAGTTAGGATCTTCTCAGTTCCCTC | 34 | | NA | | NA | | | NA | |
|  | Spacer_NoG3_64 | TGTCGAACACCTTATCTTCTCCCTCCCCGCCGTTGCG | 37 | | NA | | NA | | | NA | |
|  | Spacer_NoG3_65 | GATATAAGTCTGACTGTCTACCCAGTAGACACAGCA | 36 | | NA | | NA | | | NA | |
|  | Spacer_NoG3_66 | TTGAAGCAGTGGAAATGGACGTGTACACGGCTA | 33 | | NA | | NA | | | NA | |
|  | Spacer_NoG3_67 | TTTATGGATCATCTATATATCCTGGAAGGCAATCAT | 36 | | NA | | NA | | | NA | |
|  | Spacer_NoG3_68 | GTCCATCTATTTGCGGCTATAAAGGAGGTGCGGACGA | 37 | | NA | | NA | | | NA | |
|  | Spacer_NoG3_69 | TCGAGGTGGTATTTGAAGGTAGATACAGGTACTCCAT | 37 | | NA | | NA | | | NA | |
|  | Spacer_NoG3_70 | TCTCCTCTTGATAAGAGGGAGAATGGAGTCATTCT | 35 | | NA | | NA | | | NA | |
|  | Spacer_NoG3_71 | AGCCCCTTAAAATAATGTTTTTTAATAAAATCATGATGTCA | 41 | | NA | | NA | | | NA | |
|  | Spacer_NoG3_72 | TAGGATCTGTCTCAATCTCTTTTGTAGACTCCACTA | 36 | | NA | | NA | | | NA | |
|  | Spacer_NoG3_73 | GCAACAGCCACGGGTAGGCTTGCACGTCCGTTTAACAA | 38 | | NA | | NA | | | NA | |
|  | Spacer_NoG3_74 | CAGGAAATAAGTCCGAATTAGAATTGATACAGGAGC | 36 | | NA | | NA | | | NA | |
|  | Spacer_NoG3_75 | GACCTACTGATGTTTTGGCAACAGAGGATGGCACGAT | 37 | | NA | | NA | | | NA | |
|  | Spacer_NoG3_76 | GCACCATACTTAGATCGTATTCAAATCTTTGGGTACTTG | 39 | | NA | | NA | | | NA | |
|  | Spacer_NoG3_77 | AGTGTAGATTACACTGAAAATGGTGCAAATACCGCCA | 37 | | NA | | NA | | | NA | |
|  | Spacer_NoG3_78 | TTTGTACTTGAAATTCAAACCTTTTTCCTCACTG | 34 | | NA | | NA | | | NA | |
|  | Spacer_NoG3_79 | AGAAGAAGTCGGATCCTGTGATTGAA | 26 | | NA | | NA | | | NA | |
|  | Spacer_NoG3_80 | CGGTTTTACTACGGATAAGCTGATAGGAGAGTTTA | 35 | | NA | | NA | | | NA | |
|  | Spacer_NoG3_81 | CACGGGGTGAAATACCCCGATGAGATGTAGTTTGAATG | 38 | | NA | | NA | | | NA | |
|  | Spacer_NoG3_82 | TCGTATGAACTACGGAGCAACGAGGATGTCTACGAATG | 38 | | NA | | NA | | | NA | |
|  | Spacer_NoG3_83 | TTTAGGATCAATCCTTGTATTAGATAATACATTTTTT | 37 | | 21 | | AB620173 Influenza A virus PB1 gene for polymerase PB1 | | | virus | |
|  | Spacer_NoG3_84 | TCGGGGGTAACGACTTTTTTGCAACGAGTGACGCCGTAG | 39 | | NA | | NA | | | NA | |
|  | Spacer_NoG3_85 | CCTATAGAAATTTAATTTCTGTTTAACATGGATGT | 35 | | 21 | | *Clostridium* *perfringens* strain JP838 plasmid pJFP838A | | | plasmid | |
|  | Spacer_NoG3_86 | AGGGAGGGTAATATTTTATATGAGAGGTTGGGCTGT | 36 | | NA | | NA | | | NA | |
|  | Spacer_NoG3_87 | TTCATTTGGGTAGCTAAATTCTTTTGTAAGCTCTTTA | 37 | | NA | | NA | | | NA | |
|  | Spacer_NoG3_88 | AAACCCCTTTATCAACGTGCAAATAATAATGTTTT | 35 | | NA | | NA | | | NA | |
|  | Spacer_NoG3_89 | ATAATAGTAAAGATAGGTGATAGTTCCCCTAATGGA | 36 | | NA | | NA | | | NA | |
|  | Spacer_NoG3_90 | TCGGAAACTGGAGAATTATCTTAGCCTTTATAACTCT | 37 | | NA | | NA | | | NA | |
|  | Spacer_NoG3_91 | GGGATTCTTTAATGATTCCGGACATTACCAGAAA | 34 | | NA | | NA | | | NA | |
|  | Spacer_NoG3_92 | GGTAATATCTAAATTAGAATTGATTAATTCCTCTA | 35 | | NA | | NA | | | NA | |
|  | Spacer_NoG3_93 | TGTTGCCGCTTGGTGGCTGGGGCGTAAGAGCGGACA | 36 | | NA | | NA | | | NA | |
|  | Spacer_NoG3_94 | ACTGCGACGCCAAACCGGCCGCCACGTCAGACTCG | 35 | | NA | | NA | | | NA | |
|  | Spacer_NoG3_95 | CGAGGTTTGCGCCGCAGTGCGAGATCTACGAAAGCTGCAACCT | 43 | | NA | | NA | | | NA | |
|  | Spacer_NoG3_96 | ACGCCACAAATCCGGCGTTCGTCCGAGCAACGATTTTTGAA | 41 | | NA | | NA | | | NA | |
|  | Spacer_NoG3_97 | CCCTACGCTGTTACAACAATGCGGTGGAGGAGATG | 35 | | NA | | NA | | | NA | |
|  | Spacer_NoG3_98 | CCTAAAGTGTAGTTAGTATTATAAAAGCGTTTCGAC | 36 | | NA | | NA | | | NA | |
|  | Spacer_NoG3_99 | GCGGTGTTTGGCAGTCGCCACGAACGATCGCCCGTCTGCTAATTC | 45 | | NA | | NA | | | NA | |
|  | Spacer_NoG3_100 | AACAGATGAGGCTTTTAATCAAAGAGAACTACCAT | 35 | | NA | | NA | | | NA | |
|  | Spacer_NoG3_101 | GAGCTGAATTCTTTGGATTTGAATGTGGCATTAAGGA | 37 | | NA | | NA | | | NA | |
| 4 | Spacer_NoG4_001 | GGGGCGTCTGCGGGAATTTCGCCCTACCCGCACTGAAGAA | 40 | | NA | | NA | | | NA | |
|  | Spacer_NoG4_002 | AGCAGAGATAATCTCGTGGGCATTACAGATGGACAAA | 37 | | NA | | NA | | | NA | |
|  | Spacer_NoG4_003 | TCGATCTCGAGGATGATAGCGGCGACGAAGCC | 32 | | NA | | NA | | | NA | |
|  | Spacer_NoG4_004 | CACAATTGTTTTTGTCAGAGTAGTCCCCGAATTGAT | 36 | | NA | | NA | | | NA | |
|  | Spacer_NoG4_005 | TTTCTGTTACTAAGATTGCCCTTCAGCAACGGGGA | 35 | | NA | | NA | | | NA | |
|  | Spacer_NoG4_006 | AATTCTTTTTCCAATATTATCATTTTGATACTGCTGTT | 38 | | NA | | NA | | | NA | |
|  | Spacer_NoG4_007 | TAAAGTTAAGTTCTCTTTTTTCTTACCAATACTGA | 35 | | NA | | NA | | | NA | |
|  | Spacer_NoG4_008 | CTGATATAGAAACAGCTAGACGGCAACTGTTACTAATAATCACTAGATC | 49 | | NA | | NA | | | NA | |
|  | Spacer_NoG4_009 | GCGATTCCGATGTCTCCGAGTCGCTCGAAGAACT | 34 | | NA | | NA | | | NA | |
|  | Spacer_NoG4_010 | GTTCTTCAATTTAATTTCTGTTTTACAGGGATTA | 34 | | NA | | NA | | | NA | |
|  | Spacer_NoG4_011 | GGGTGTTGGCGCAAACGGTGCTTTAATTTTCTCC | 34 | | NA | | NA | | | NA | |
|  | Spacer_NoG4_012 | TCAAAAAAAATATTTACCTCTACAACGATTTGAG | 34 | | NA | | NA | | | NA | |
|  | Spacer_NoG4_013 | AAAAAATTTCGTGGGTGCGATTCAGTCCGCGTATG | 35 | | NA | | NA | | | NA | |
|  | Spacer_NoG4_014 | TAGATTCAGCCAGCACTTTATCCTGGCTACGTTGACGCGTATG | 43 | | NA | | NA | | | NA | |
|  | Spacer_NoG4_015 | TTATTTTTTTCGTCTAGCTTACTTAGCGTACCTA | 34 | | NA | | NA | | | NA | |
|  | Spacer_NoG4_016 | AAGACAAAATAGTTGAATTACAGTCCCTTTTGGCATCTC | 39 | | NA | | NA | | | NA | |
|  | Spacer_NoG4_017 | CCTTCTTCGGCGTAAACCACTGTCCCCGAATGCGTCGC | 38 | | NA | | NA | | | NA | |
|  | Spacer_NoG4_018 | ATGAAGAGTACGCTGAAACTCTTCGGTACGTCTTC | 35 | | NA | | NA | | | NA | |
|  | Spacer_NoG4_019 | GAGGGAATAGAGCGACTTGGGAAGAGGTTGCTCAAGTACGTCTTC | 45 | | NA | | NA | | | NA | |
|  | Spacer_NoG4_020 | GACATTTGTTCCTTGGTGAAGCTTTCCTTCCGCTTG | 36 | | NA | | NA | | | NA | |
|  | Spacer_NoG4_021 | TCGTATGAACTACGGAGTGGGGTGGACGTCTACCAGTG | 38 | | NA | | NA | | | NA | |
|  | Spacer_NoG4_022 | TTTAGATAAGGATCTACGTCCCGTTAAACCAGAA | 34 | | NA | | NA | | | NA | |
|  | Spacer_NoG4_023 | GTTCTATCACATTTAGAGGCAAACTCTGACTGTGTTA | 37 | | NA | | NA | | | NA | |
|  | Spacer_NoG4_024 | GGGAGCGACCTACAAGCTGGGATGTGTTACTATTACCA | 38 | | NA | | NA | | | NA | |
|  | Spacer_NoG4_025 | AGTATGAACTAGGCGTTTCCCTTCCTGGTTGTAGATCTTCAGATCTAC | 48 | | NA | | NA | | | NA | |
|  | Spacer_NoG4_026 | ATCTCTCTACTTTACTCGGGTTAAATTGTTCCGCTAT | 37 | | NA | | NA | | | NA | |
|  | Spacer_NoG4_027 | GAGAAGGGTTTGGGTCGTTAAAGGTATATTGTAGATACAG | 40 | | NA | | NA | | | NA | |
|  | Spacer_NoG4_028 | CAGGCACTTTCTTTTCCCTCCGTGTTATTATAAA | 34 | | NA | | NA | | | NA | |
|  | Spacer_NoG4_029 | CCTAAAACCCTTGTCCCGTTTAATTCCTGTAA | 32 | | NA | | NA | | | NA | |
|  | Spacer_NoG4_030 | TTGAATGCTGTGCCTTTTTGGCGACGCTCAAGG | 33 | | NA | | NA | | | NA | |
|  | Spacer_NoG4_031 | TTAAAAAAGGAGGATACTCTGAGAAGTATGGATTT | 35 | | NA | | NA | | | NA | |
|  | Spacer_NoG4_032 | ATATTTTATATGAGAGGTTGGGCTGCAACTATAG | 34 | | NA | | NA | | | NA | |
|  | Spacer_NoG4_033 | GGTATATAAGTCTCTATGGTCATGTTAGAGGCCTCTATAAG | 41 | | NA | | NA | | | NA | |
|  | Spacer_NoG4_034 | CTGAGGGCGTAAATTCTTCTTGACCATTTAAACCT | 35 | | NA | | NA | | | NA | |
|  | Spacer_NoG4_035 | AAGGGTAGTGACGAGTCCGTTAAGATTAAAGATCTT | 36 | | NA | | NA | | | NA | |
|  | Spacer_NoG4_036 | TTTGATTCCCGAAGCGATCGCGAACCATCAACA | 33 | | NA | | NA | | | NA | |
|  | Spacer_NoG4_037 | TTCATCTCAGCACCCCTCGTCCACAGCAACATGC | 34 | | NA | | NA | | | NA | |
|  | Spacer_NoG4_038 | TTTAAGGTAGTTCTACCTTGGGGAAATGACAATATGCT | 38 | | NA | | NA | | | NA | |
|  | Spacer_NoG4_039 | TCAAATCCGGTCTGTGGCTCGTCCAGCGTGATGATG | 36 | | NA | | NA | | | NA | |
|  | Spacer_NoG4_040 | ATAATTTACTTAACAGATTTAGTACTTTTTTCT | 33 | | NA | | NA | | | NA | |
|  | Spacer_NoG4_041 | ATTTTATTTGCATAGTTGAAGGCTGGAATAAAGA | 34 | | NA | | NA | | | NA | |
|  | Spacer_NoG4_042 | GAGGATCGCTTGGGCGATCGTCACCGAGGCCGG | 33 | | NA | | NA | | | NA | |
|  | Spacer_NoG4_043 | TTGAGTTTGGTGAACAGACGATTGACCCGTCATT | 34 | | NA | | NA | | | NA | |
|  | Spacer_NoG4_044 | GAAATAATATAGTTATCATGTTATCACATAAAAAT | 35 | | NA | | NA | | | NA | |
|  | Spacer_NoG4_045 | TCTCCTTCTCTAATTCAATTAAAATCATCTCAGCTAG | 37 | | NA | | NA | | | NA | |
|  | Spacer_NoG4_046 | TGGTCGCGATGCTCGTAGTCGACTGCGATCGC | 32 | | NA | | NA | | | NA | |
|  | Spacer_NoG4_047 | GAGCCGCTATCTAAGGGATGAAAGAGGTATAAGCCCT | 37 | | NA | | NA | | | NA | |
|  | Spacer_NoG4_048 | CCGTCATCCAGTGGATCGCATCTAATCTCGATTG | 34 | | NA | | NA | | | NA | |
|  | Spacer_NoG4_049 | TATAAAAACTACATGATCCGTGAAGCACACTATAAAG | 37 | | NA | | NA | | | NA | |
|  | Spacer_NoG4_050 | CACTAATTTAATCATTGACATATTTAATAGTTCCATC | 37 | | NA | | NA | | | NA | |
|  | Spacer_NoG4_051 | CAGGCAAATTACTAAGGGATAAAGGTTTAATATTG | 35 | | NA | | NA | | | NA | |
|  | Spacer_NoG4_052 | AAAATCATTTATATAGGTGATATCCTTTACATCACC | 36 | | NA | | NA | | | NA | |
|  | Spacer_NoG4_053 | GATCGGGGCTGTTGACTCTGCCAACAAATGATCCGTTC | 38 | | NA | | NA | | | NA | |
|  | Spacer_NoG4_054 | AGGGCTAAAAGATTGCGACGATATCGATCTTCTAC | 35 | | NA | | NA | | | NA | |
|  | Spacer_NoG4_055 | TCGCGGACGATCCCTATCGATGTTTGACTGTTGAAGGTTTTTAT | 44 | | NA | | NA | | | NA | |
|  | Spacer_NoG4_056 | CAGATCCCGATGGCGTAATTGCATCTTATCTAGATCCG | 38 | | NA | | NA | | | NA | |
|  | Spacer_NoG4_057 | TGGATAGTAGGCTCGAAATCCGTGTACTATGGCTTCC | 37 | | NA | | NA | | | NA | |
|  | Spacer_NoG4_058 | ATGAAGATGTTATCACCCCAGGTGAACAGGAACCTG | 36 | | NA | | NA | | | NA | |
|  | Spacer_NoG4_059 | AGGATTTTAAGTCCTCCCTATTAAATAATGTCAAT | 35 | | NA | | NA | | | NA | |
|  | Spacer_NoG4_060 | CGGTGGAGTGCTACTATATCGGGGTATAAAGCGCCCAC | 38 | | NA | | NA | | | NA | |
|  | Spacer_NoG4_061 | CCTCAATCTTTGGTGCTTCTCAATTAAGCTTCGA | 34 | | NA | | NA | | | NA | |
|  | Spacer_NoG4_062 | AGTTATTCACTCACTTCAATGACACAAAGGAAAG | 34 | | NA | | NA | | | NA | |
| 5 | Spacer_NoG5_01 | ACAGGAGTGGGAAATCGCACGATTTCTAGTCACATC | 36 | | NA | | NA | | | NA | |
|  | Spacer_NoG5_02 | ACTCGGGGAACTCGGCTATATCTCTCGCTTCCACC | 35 | | NA | | NA | | | NA | |
|  | Spacer_NoG5_03 | TGTCGGCGGTGCAGTACGCCGAAACACCGTTCACCA | 36 | | NA | | NA | | | NA | |
|  | Spacer_NoG5_04 | ACTTTAGCGGCGATGGAATTAGTGTCGGTGGGCATCGC | 38 | | NA | | NA | | | NA | |
|  | Spacer_NoG5_05 | TCGGCTTTTCCAAAGAGTGATAGTTGTTGTTGTTT | 35 | | NA | | NA | | | NA | |
|  | Spacer_NoG5_06 | CTTTCGCTCCCGTCACTCGATAGACCTTTGTCAC | 34 | | NA | | NA | | | NA | |
|  | Spacer_NoG5_07 | AAATCGGCGCGGCGGCGTACCGCGACGAAAACCAGCC | 37 | | NA | | NA | | | NA | |
|  | Spacer_NoG5_08 | ATTAAACGACCTAGACTGCCGGAGCCTATCGCGGA | 35 | | NA | | NA | | | NA | |
|  | Spacer_NoG5_09 | TTTGATATCGAATTCAGGGGCAATTCCTACCCTATCCT | 38 | | NA | | NA | | | NA | |
|  | Spacer_NoG5_10 | GACACTCCGGTAACAGTCAGCCGCTCGGCGACGATT | 36 | | 20 | | *Sphingobium* sp. EP60837 plasmid pEP2 | | | plasmid | |
|  | Spacer_NoG5_11 | ATTAATCCCGCCTTCCCCATCTCGATCGCCCAGATT | 36 | | NA | | NA | | | NA | |
|  | Spacer_NoG5_12 | GAATAATTCGCCCCAAATTCCCACCATCTGGGCGTT | 36 | | NA | | NA | | | NA | |
|  | Spacer_NoG5_13 | CTTACAGCCCGCTGTATAGCACGCCGAAGGGTTTTT | 36 | | NA | | NA | | | NA | |
|  | Spacer_NoG5_14 | AAAACTACCGCCCCTGCAGGGGAATTAATCTACCT | 35 | | NA | | NA | | | NA | |
|  | Spacer_NoG5_15 | TTAATACATACAGGTATTGTACCATGTTTTCGCCAA | 36 | | NA | | NA | | | NA | |
|  | Spacer_NoG5_16 | GTTCCCCTTGCAATCGTCGCTGTTCGTCAGCCCAAT | 36 | | NA | | NA | | | NA | |
|  | Spacer_NoG5_17 | GGTTTGGAGGATTATCGTTAAATGACTTCATTAC | 34 | | NA | | NA | | | NA | |
|  | Spacer_NoG5_18 | ATCTCAAGGAACTTGAATGTCTGTAACAACATAACA | 36 | | NA | | NA | | | NA | |
|  | Spacer_NoG5_19 | AACCGCACTCGGTTTTGCGTCCTGATGTTTGGTTT | 35 | | NA | | NA | | | NA | |
|  | Spacer_NoG5_20 | GTTGCAGACGACGATCGCCTCGGAAGTCGTCCCCCT | 36 | | NA | | NA | | | NA | |
|  | Spacer_NoG5_21 | CGGCATCAGCAACCAAGCAGGCATCCTCTACGGGTTC | 37 | | NA | | NA | | | NA | |
|  | Spacer_NoG5_22 | CGTCCGCAAGCTGACGGGCGATCGCAGCGTTCC | 33 | | NA | | NA | | | NA | |
|  | Spacer_NoG5_23 | TGTACCAGGGGGTTTCGCCCTGGGGAGAAAGGAATA | 36 | | NA | | NA | | | NA | |
|  | Spacer_NoG5_24 | AGACGCCCGCAACAGGACGAAGACGAAGAAGAGA | 34 | | NA | | NA | | | NA | |
|  | Spacer_NoG5_25 | GCGTTGGGGACGACGACGGTGTTGGCGTCGGCCG | 34 | | 24 | | *Halomicrobium* *mukohataei* DSM 12286 plasmid pHmuk01 | | | plasmid | |
|  | Spacer_NoG5_26 | AGTGAACCGTTCGCGATACAAGGGGCGGCGATACC | 35 | | NA | | NA | | | NA | |
|  | Spacer_NoG5_27 | CGACGCTGCCAGTGCCGCAGCCCTGCATCGCCAAGTC | 37 | | 27 | | GU936714_GU936714 *Synechococcus* phage S-CBS2 | | | phage | |
|  | Spacer_NoG5_28 | TCCACTGAAACCGTTGCCTCGGGATACATTCCCAA | 35 | | NA | | NA | | | NA | |
|  | Spacer_NoG5_29 | CGAGTAAAAACGGAACTCGAAAAAATTGCCGAAAAGTA | 38 | | 20 | | *Escherichia* *coli* UMN026 plasmid p1ESCUM | | | plasmid | |
| 6 | Spacer_NoG6_02 | GTCCGAAGATGGTGAGGTTTTTATCTCGCTTTAGTACCC | 39 | | NA | | NA | | | NA | |
|  | Spacer_NoG6_03 | TAAATATGAATCCAGAACTTGAATTTTTGTTGCAGCTA | 38 | | 20 | | KF148616 *Campylobacter* phage CP8 | | | phage | |
|  | Spacer_NoG6_04 | ATTACGCTATCTTGGTTAATCGTGGCGGACGGTTTCGA | 38 | | NA | | NA | | | NA | |
|  | Spacer_NoG6_05 | GAAAATCCCCTGAGTTGGTTGGCGCTCGATTTGACCAAAAACTGAA | 46 | | NA | | NA | | | NA | |
|  | Spacer_NoG6_06 | TTATGCACTTGTCGATCTATCGTTCGGTGATCCTGCACCG | 40 | | NA | | NA | | | NA | |
|  | Spacer_NoG6_07 | GGGTATATAGCCCCCGCGGTCGTACACCTCAACACAA | 37 | | NA | | NA | | | NA | |
|  | Spacer_NoG6_08 | TTGGTGATAACCTATACCCCGGAGTAACAACAATACTAAA | 40 | | NA | | NA | | | NA | |
|  | Spacer_NoG6_09 | CCTCGAAAGAGGGCGTTATTTCTGTAGCCGAAATCAAAAA | 40 | | NA | | NA | | | NA | |
|  | Spacer_NoG6_10 | AAAGTTCGGTATCGGCTATTCGCCTCGAGGCAAGCTCCT | 39 | | NA | | NA | | | NA | |
|  | Spacer_NoG6_11 | ATGCCGTCGGGATGAACCTGTCAGCCGAGACGCTGAGGACT | 41 | | NA | | NA | | | NA | |
|  | Spacer_NoG6_12 | TTTATCGACTCCAAAACGATCCCCCTGGCAACAGATA | 37 | | NA | | NA | | | NA | |
|  | Spacer_NoG6_13 | AGAAAGATCGTGAATTTAAAGATGGAGCCAAAATAAA | 37 | | 21 | | AP008983 *Clostridium* phage c-st | | | phage | |
|  | Spacer_NoG6_14 | ATGGACGCAGGGGCTACGGTTCCCAGATACGCCGTCGA | 38 | | NA | | NA | | | NA | |
|  | Spacer_NoG6_15 | GACGCCGTAGACTTCTACACTCCCAATCTGGATGCAATATTTG | 43 | | NA | | NA | | | NA | |
|  | Spacer_NoG6_16 | AGGATTTATGCAATTGGAGTCGCACTCTCAACTAGGGAT | 39 | | NA | | NA | | | NA | |
|  | Spacer_NoG6_17 | GAGAGAAGTAGCGATGAATTGTATTGATATTGTGCGGGA | 39 | | NA | | NA | | | NA | |
|  | Spacer_NoG6_18 | TCAACGTATATTTATTAGTACCGATATCAAAATTAGTATG | 40 | | 20 | | HF679131 *Adoxophyes* *honmai* enomopoxvirus 'L' | | | virus | |
|  | Spacer_NoG6_19 | CCTACTTGCCACCCAGTAGAATTGTCGGTATAAATTAAA | 39 | | NA | | NA | | | NA | |
|  | Spacer_NoG6_20 | GATCTCTAAGAGTTTACACCCGTTGCGGAGTTGTTGG | 37 | | NA | | NA | | | NA | |
|  | Spacer_NoG6_21 | ACGACGTCAATCGAGCGCTACGACTCGTTCGTACGT | 36 | | NA | | NA | | | NA | |
|  | Spacer_NoG6_22 | GCTTTGGGGATGTTGCCCAAGGACATAACGATTTTTT | 37 | | NA | | NA | | | NA | |
|  | Spacer_NoG6_23 | TTTATTGTACGAGCGGGAGGGTACCTCCCAGACTTGGAG | 39 | | NA | | NA | | | NA | |
|  | Spacer_NoG6_24 | TTTAGCGGCTCCAGACCAATTGATTTCATTATTGTTAT | 38 | | 20 | | AF020713 Bacteriophage SPBc2 complete genome | | | phage | |
|  | Spacer_NoG6_25 | TGGCTATCAGACATCAACCCCAATCTGGCGCTGACGTAT | 39 | | NA | | NA | | | NA | |
|  | Spacer_NoG6_26 | GTGGACGGGATTATTTAGTATACGATCCATGGGATTAC | 38 | | NA | | NA | | | NA | |
|  | Spacer_NoG6_27 | AGGGCTTATGGTTTAAGTATTCAGGGTCGCTTTGATTGG | 39 | | NA | | NA | | | NA | |
|  | Spacer_NoG6_28 | AAATTACGCAAAAGGCTGCCTGGCAAATTGTGTATCGACTGG | 42 | | NA | | NA | | | NA | |
|  | Spacer_NoG6_29 | TGCTTGGTGAGCGGTTTTTGTCCACTGATTTAAT | 34 | | NA | | NA | | | NA | |
|  | Spacer_NoG6_30 | TTTACCGCAACGGACACGCCGACGAACGTGCGGGAT | 36 | | NA | | NA | | | NA | |
|  | Spacer_NoG6_31 | GTAACAGCGCAATCAGTTTCTATGGCTGCGTTAAAGCTGTA | 41 | | NA | | NA | | | NA | |
|  | Spacer_NoG6_32 | GAGGAATATTTTCCGGAAGTGTCGGCGCGTCACTCCC | 37 | | NA | | NA | | | NA | |
|  | Spacer_NoG6_33 | TCTTCTACCGATAGAGTTACGTCTTATTTTGTAGATTGAGT | 41 | | NA | | NA | | | NA | |
|  | Spacer_NoG6_34 | CCCGCTACGGCTGTCGGACTCCAAATCGTATTTAATA | 37 | | NA | | NA | | | NA | |
|  | Spacer_NoG6_35 | TATTAGTATTAGTAAATAGAGAGCCTCTGATCGAACAAAC | 40 | | NA | | NA | | | NA | |
|  | Spacer_NoG6_36 | CTTTGGGATATAACCCCAACGAGTCGTTTCCCGCAGACA | 39 | | NA | | NA | | | NA | |
|  | Spacer_NoG6_37 | TTGACCGTCGTGCAACAGTTGGGTGGAAGCTTGGGG | 36 | | NA | | NA | | | NA | |
|  | Spacer_NoG6_38 | TTTTCGTTAAGGGCAACGATTTCTTCATCGAATCCCT | 37 | | NA | | NA | | | NA | |
|  | Spacer_NoG6_39 | AAGCAAGGCAAGTATTACGTCGAGGACTCCATCAAGCA | 38 | | NA | | NA | | | NA | |
|  | Spacer_NoG6_40 | TTGTCGATCTTCAGATGGAATATTTCGAGCTTGGCA | 36 | | NA | | NA | | | NA | |
|  | Spacer_NoG6_41 | TTTAATTTTTATGGACACTTTGACTTCGGGACGTT | 35 | | NA | | NA | | | NA | |
|  | Spacer_NoG6_42 | ACTGTCGGACGTGGGAAGGATATCCCCGCGCTCACAA | 37 | | NA | | NA | | | NA | |
|  | Spacer_NoG6_43 | GGTAACACGACCTATCGCTACCGTATTAAAGAAGATTT | 38 | | NA | | NA | | | NA | |
|  | Spacer_NoG6_44 | AACCCTACAACGCCTTCAACAAGCCAGAGGACAACGC | 37 | | NA | | NA | | | NA | |
|  | Spacer_NoG6_45 | AAAACGGCGAATCGCTTGCTCCCTACCTCCGTCAGAT | 37 | | NA | | NA | | | NA | |
|  | Spacer_NoG6_46 | GCGAATTACCTGCGTGGGTCGTCAGCACCTCGCTCATTTT | 40 | | NA | | NA | | | NA | |
|  | Spacer_NoG6_47 | ATCCCGTACCTCGTAAGGGGCCGCTCGACCTCGAT | 35 | | NA | | NA | | | NA | |
|  | Spacer_NoG6_48 | ACCGTTCGACCTCGAGATAGAGCGGAACGAATACGATTC | 39 | | 21 | | KU760999 Bluetongue virus isolate BTV-27FRA2014v03 segment 3 viral protein 3 (VP3) | | | virus | |
|  | Spacer_NoG6_49 | AATTTAAGCTTGGTCTTTTCGCGGTTTTTACCTCCTTT | 38 | | NA | | NA | | | NA | |
|  | Spacer_NoG6_50 | TGTCTGTTTATCTCCACCGGGTTGTCCAACCCCATAGAG | 39 | | NA | | NA | | | NA | |
|  | Spacer_NoG6_51 | TGACTTCGGCGTTGATTATAAGCCGTTAACGTGGCGAA | 38 | | NA | | NA | | | NA | |
|  | Spacer_NoG6_52 | GTCTGCAAACCGTTCCCGTCATCGAAACATCCTTTTACTT | 40 | | NA | | NA | | | NA | |
|  | Spacer_NoG6_53 | CTGACCAAAGCGCTGCCGTTGGAGCTATCCGAAGGAG | 37 | | NA | | NA | | | NA | |
|  | Spacer_NoG6_54 | ATGCGATCGGGGACTTGGGATTTCTCAATCCCAAGTGTG | 39 | | NA | | NA | | | NA | |
|  | Spacer_NoG6_55 | ATCCCGGCATGGTCGCACCCCAACGTTGCCGATAAATA | 38 | | NA | | NA | | | NA | |
|  | Spacer_NoG6_56 | TCGAACACGCTTACGCCGTGGCCGAAGCGTTCCGCGCC | 38 | | NA | | NA | | | NA | |
|  | Spacer_NoG6_57 | TTGGCGTTCTTCCAATCCCTTCTAATCTATCCACGTATT | 39 | | NA | | NA | | | NA | |
|  | Spacer_NoG6_58 | GATTTCGGCGTTTACCAGATATTGCACGTCACGAAC | 36 | | NA | | NA | | | NA | |
|  | Spacer_NoG6_59 | ATCCAGAGAGAGAAAACGGTAGCGACGATTCGCTTTTT | 38 | | NA | | NA | | | NA | |
|  | Spacer_NoG6_60 | TTGCTCGTACTGCTTCCTAGAGAATAACGACACTCCGACAG | 41 | | NA | | NA | | | NA | |
|  | Spacer_NoG6_61 | TTTTAGGGGAAAGATAGCCGATCTTTTCCGCCACCCT | 37 | | NA | | NA | | | NA | |
|  | Spacer_NoG6_62 | CGATATGAACACCAACGGCTTTTTTCAGCCAGGGAC | 36 | | NA | | NA | | | NA | |
|  | Spacer_NoG6_63 | TCAGCGTGATTCACAAAAGTCGCTTCAATAAAAGCCTCGAAAT | 43 | | NA | | NA | | | NA | |
|  | Spacer_NoG6_64 | CTCAGCCGTCTCGGTTGCCTCAGGTTCCTCAGGTTCCGTCTT | 42 | | 20 | | KF056323 *Haloarcula* *hispanica* pleomorphic virus 2 | | | virus | |
|  | Spacer_NoG6_65 | CTTACGCCGTGGGCGTTGCTCTCGATCGCTACGAAAAA | 38 | | NA | | NA | | | NA | |
|  | Spacer_NoG6_66 | CTTTGAACGCCTTTAACCGCTTATCGGGCTTCGTTGTTA | 39 | | NA | | NA | | | NA | |
|  | Spacer_NoG6_67 | TGAAATACCTGTAATTGGTTTAGAGGGATTGAAAGGAAC | 39 | | NA | | NA | | | NA | |
|  | Spacer_NoG6_68 | TACTGGAGGTATCGGACGGTACAGCTCGCATTGAACAA | 38 | | NA | | NA | | | NA | |
|  | Spacer_NoG6_69 | AAATTTTTATTTGTAGTCATGGGATTAAATCTAGTTTTT | 39 | | NA | | NA | | | NA | |
|  | Spacer_NoG6_70 | TGAACTGGAGTTTAAAAGATTAATCAACTCCTCATTTTTT | 40 | | NA | | NA | | | NA | |
|  | Spacer_NoG6_71 | ATGGGGCGAAATGCGAACCTGTTACTATCAGTTA | 34 | | NA | | NA | | | NA | |
|  | Spacer_NoG6_72 | TGTCATAATGCCAGATGGGTTACAAATTATCAATGTTA | 38 | | NA | | NA | | | NA | |
| 7 | Spacer_NoG7_02 | GCTGTTCGATGGATGCGGAGGTGGGGTCGGCGATTC | 36 | | NA | | NA | | | NA | |
|  | Spacer_NoG7_03 | CAACTTCCACACCCACTCCAGACGTAGTCGACGTCGACGA | 40 | | NA | | NA | | | NA | |
|  | Spacer_NoG7_04 | TACAATAATACCTAAGAGTAGGCTGCTGAGAAGGCTAATT | 40 | | NA | | NA | | | NA | |
|  | Spacer_NoG7_05 | GGGGTGGTTTCCCGGACGGGGGTTGCAGAGACAA | 34 | | NA | | NA | | | NA | |
|  | Spacer_NoG7_06 | CTCTTCCTCCTCGCCTTCGGCCCGGCTCGAAATCTCCGTC | 40 | | 24 | | JF974315 *Rhizobium* phage RR1-B genomic sequence | | | phage | |
|  | Spacer_NoG7_07 | CGTTGTTCTCGCTCAGCTTGTTGTTTCCAACAAAGAACA | 39 | | NA | | NA | | | NA | |
|  | Spacer_NoG7_08 | AAGACGACCCCGATTATTAAAATTCCTCCAGCGTATGAAG | 40 | | NA | | NA | | | NA | |
|  | Spacer_NoG7_09 | AATCTAGACTAGTTTTTTTAAAAAAACTAGCCCAATCAAAACA | 43 | | NA | | NA | | | NA | |
|  | Spacer_NoG7_10 | ATTGCTCGTGCAGGAGTGGTGGAAGAATTGACCAAACC | 38 | | NA | | NA | | | NA | |
|  | Spacer_NoG7_11 | TCCATCATTGGAAACTTCATCAACGCCTTTCGG | 33 | | NA | | NA | | | NA | |
|  | Spacer_NoG7_12 | CACCACGCCCAAGTACACCTTGGGTGTAATCCAAGA | 36 | | NA | | NA | | | NA | |
|  | Spacer_NoG7_13 | ATTCTACCACATCTTCTTTGGAACGAAGGATATTAAAAT | 39 | | NA | | NA | | | NA | |
|  | Spacer_NoG7_14 | ATTTCCATCGTCGCTGTCATCTTTGAAAGCGTTGCCATAT | 40 | | 20 | | HM144385 *Brochothrix* phage NF5 | | | phage | |
|  | Spacer_NoG7_15 | ATTTCGAACTATCGCTACGACGAGAGCCTACCTCTTT | 37 | | NA | | NA | | | NA | |
|  | Spacer_NoG7_16 | TACTCGCCTAAGTTTGTAGCCTCCCCTGTCTCATCATCGA | 40 | | NA | | NA | | | NA | |
|  | Spacer_NoG7_17 | ATATAGATCTAGGGGGTTTGCGTTTCCCTCAAACCTTAAGA | 41 | | NA | | NA | | | NA | |
|  | Spacer_NoG7_18 | AGTTCTTTGCAAGAACTCGCCACAATATTGGCAGTAGGT | 39 | | NA | | NA | | | NA | |
|  | Spacer_NoG7_19 | TCGTACCGGTAAGCCCGGCCGTACCGGCGAGCCTTTGCC | 39 | | NA | | NA | | | NA | |
|  | Spacer_NoG7_20 | CGTGTAGTCTTTAGCGAGAACCTCAGCGGACTCTTTAGCG | 40 | | NA | | NA | | | NA | |
|  | Spacer_NoG7_21 | CCCTCAATTAAGTGAGGGAGTCGGGACTAACTAATA | 36 | | NA | | NA | | | NA | |
|  | Spacer_NoG7_22 | CAGATTGTATACTCCCCGTCCTTCTTCATGGACTGGGA | 38 | | NA | | NA | | | NA | |
|  | Spacer_NoG7_23 | AATATTGAATACTTCTTCTAGGGAATTGAAATTCCCGAACG | 41 | | NA | | NA | | | NA | |
|  | Spacer_NoG7_24 | CTTCCACCGCTACTGCCAAGGGGCTAGTGATAGCGAA | 37 | | NA | | NA | | | NA | |
| 8 | Spacer_NoG8_02 | GTCGGAAAAAACCCAGAGCTATAAAAATGTACTTGTCCCGATAATTGCTTT | 51 | | NA | | NA | | | NA | |
|  | Spacer_NoG8_03 | TTGAAAAAAAACTCCAACAATATTACCCCAAAAAACGAGAAAATTCTTGA | 50 | | 22 | | *Ilyobacter* *polytropus* DSM 2926 plasmid pILYOP01 | | | plasmid | |
|  | Spacer_NoG8_04 | GTCAAAAAAAAACGAAATTTGACAAATGCTAAAAGATAACTGAATT | 46 | | 20 | | *Lactobacillus* *salivarius* UCC118 plasmid pMP118 | | | plasmid | |
| 9 | Spacer_NoG9_01 | TATTCTATTGGGTCAGGACAACTTTAATCTGTTGACCCGTTTT | 43 | | NA | | NA | | | NA | |
|  | Spacer_NoG9_02 | CTCTGGCTCATCAGCTCTCCCTAGAGCGAATAGACAGTTTC | 41 | | NA | | NA | | | NA | |
|  | Spacer_NoG9_03 | TCTCGAAACGCCCGTGAAACTTACCGTCACAAAATGTTTT | 40 | | NA | | NA | | | NA | |
| 10 | Spacer_NoG10_01 | GGGCCAAGAAAGCCCCGATCCCAATCCCAATCCCATG | 37 | | NA | | NA | | | NA | |
|  | Spacer_NoG10_02 | CGGGAACTTACCTCACTGCCGAAGACGCCATCCATG | 36 | | NA | | NA | | | NA | |
|  | Spacer_NoG10_03 | AAAACGTTTACGGGCTAACCCGTTTCCCCGGAACTTA | 37 | | NA | | NA | | | NA | |
|  | Spacer_NoG10_04 | TATTTGTCTCCCGAAGACACAATTCATGCTGGAT | 34 | | NA | | NA | | | NA | |
|  | Spacer_NoG10_05 | TCAAAGAGTTCCTGTGAGGAAGCGCTAGCCCGTAGCCGAGAGG | 43 | | NA | | NA | | | NA | |
|  | Spacer_NoG10_06 | GTTTGGGACTTACGAGTCCGCCGAAGCCGCCATTTACGATG | 41 | | NA | | NA | | | NA | |
|  | Spacer_NoG10_07 | CGTTCTCGGTATCGCTAACGCTACGTTAGCGACGCCTAT | 39 | | NA | | NA | | | NA | |
|  | Spacer_NoG10_08 | GTTCTTGCAATCAACACCGAGCAAGGCTCTTGTTTCTTAGA | 41 | | NA | | NA | | | NA | |
|  | Spacer_NoG10_09 | CGTCGTTGCTATCAACAGCAACCAGGGGCTATGTTTT | 37 | | NA | | NA | | | NA | |
|  | Spacer_NoG10_10 | TATACTGACTTCATTACCGACCCAAAGACTGGAAAACAA | 39 | | NA | | NA | | | NA | |
|  | Spacer_NoG10_11 | GGCTACTGTAGCCCGGGACCGGTCTATGAGACTGAT | 36 | | NA | | NA | | | NA | |
|  | Spacer_NoG10_12 | CCGCAATTTACAATGGGGGGGTATGTCCCTCCTCAC | 36 | | NA | | NA | | | NA | |
|  | Spacer_NoG10_13 | AGTACGTCATCGCCATCAGTGACGGCCGATCATGCTATT | 39 | | NA | | NA | | | NA | |
| 11 | Spacer_NoG11_15 | CCGTCGCAATTAAAGATGAGGAGCTGCACGACATCGC | 37 | | NA | | NA | | | NA | |
|  | Spacer_NoG11_16 | CTCTTGCTATTTAGCTTCAAGGGGTATCGTATGCCATAA | 39 | | NA | | NA | | | NA | |
|  | Spacer_NoG11_17 | TCTTTGCTACCTCGACGGTAGCAAGCCCAGTTCA | 34 | | NA | | NA | | | NA | |
|  | Spacer_NoG11_18 | CTTTTCATCGCTGTCGTAATCTTTTTCGTTCGGAA | 35 | | NA | | NA | | | NA | |
|  | Spacer_NoG11_19 | TTATGTTGTTTGATGATGATGATGACGACGGGGAGAAAA | 39 | | NA | | NA | | | NA | |
|  | Spacer_NoG11_20 | TGGAAACGTGAGGAGGGACATACCCCCCATT | 31 | | NA | | NA | | | NA | |
|  | Spacer_NoG11_21 | CCCATTCCAAGCTCGTTAGAAGGGGAAAACCCCGTCTT | 38 | | NA | | NA | | | NA | |
|  | Spacer_NoG11_22 | TCAGCCCCCATACCTCGTCTTGCGACTTCGCGGAGA | 36 | | NA | | NA | | | NA | |
|  | Spacer_NoG11_23 | CCCTTCGCTTCGGGGTAGAATATGAAAGCTGCGATGATTT | 40 | | NA | | NA | | | NA | |
|  | Spacer_NoG11_24 | TTTTGGGTTCAGTTTCTTACTGAACTTTCCAAGTTAT | 37 | | NA | | NA | | | NA | |
|  | Spacer_NoG11_25 | GAAGCCCATCGCGGGTCGGGTCGTATCGGGTAATTAGC | 38 | | NA | | NA | | | NA | |
|  | Spacer_NoG11_26 | TGTTCGCAACGGCGTGCTTGACTGTATTGCTGTCTACGACAACGA | 45 | | NA | | NA | | | NA | |
|  | Spacer_NoG11_27 | AAGAGGAGTCCGACGAGGGTCTAGATCCTACGATTA | 36 | | NA | | NA | | | NA | |
|  | Spacer_NoG11_28 | TCCTTCGCCACCCCCACGATTGTAGCTAAAGAATACACCCCAG | 43 | | NA | | NA | | | NA | |
|  | Spacer_NoG11_29 | TCTGTAGCAGGCGTGCCCTCTGTAGCTAAAGAATACATCCCG | 42 | | NA | | NA | | | NA | |
|  | Spacer_NoG11_30 | ATTTTCATCCTCTTTCTAGGAGGGGGTGTGATTACCA | 37 | | NA | | NA | | | NA | |
|  | Spacer_NoG11_31 | TGACAAATTATTTGAAGAAGGAGGTGAGGAATGTGAC | 37 | | NA | | NA | | | NA | |
|  | Spacer_NoG11_32 | CTCTGAAATATCTGAAATAGAGAAGAGAGTCCCCCGAAC | 39 | | NA | | NA | | | NA | |
|  | Spacer_NoG11_33 | CGTCGTCGGTCTCCATGGTCCTCCCCGGACAAGAAGGTGAT | 41 | | NA | | NA | | | NA | |
|  | Spacer_NoG11_34 | CAGCCCCACTCCCTCGAGTGTATGAAGGAGATATGGGTATG | 41 | | NA | | NA | | | NA | |
|  | Spacer_NoG11_35 | ACGGAAGTTGTGGAATTTCAAACCCCTCTTCAAA | 34 | | NA | | NA | | | NA | |

**Table S7 Phage related genes in *Roseofilum* *reptotaenium* AO1.** Annotations were taken from RAST and BLASTp.

| Feature ID | Contig | RAST annotations | BLAST similarity |
| --- | --- | --- | --- |
| fig\|564709.3.peg.63 | Contig_102_length_22140_cov_415.14_ID_2031875719653897 | phage protein | putative lipoprotein [Vibrio phage CKB-S1], E-value 2e-45 |
| fig\|564709.3.peg.309 | Contig_123_length_7114_cov_1067.01_ID_24544845416933 | phage protein | **1)** Multi-domain Transposase [Mobilome: prophages, transposons], COG3415,  E-value 2.47e-08.  **2)** Gp20, Siphoviridae,  E-value: 8.2e-30 |
| fig\|564709.3.peg.623 | Contig_15_length_308535_cov_441.995_ID_292752362770261791 | phage tail sheath protein | Multi-species: tail protein [Arthrospira], E-value 0.0 |
| fig\|564709.3.peg.624 | Contig_15_length_308535_cov_441.995_ID_292752362770261791 | hypothetical protein | T4-like virus tail tube protein gp19, pfam06841,  E-value 1.10e-17 |
| fig\|564709.3.peg.625 | Contig_15_length_308535_cov_441.995_ID_292752362770261791 | hypothetical protein | T4-like virus tail tube protein gp19, pfam06841,  E-value 3.64e-16 |
| fig\|564709.3.peg.626 | Contig_15_length_308535_cov_441.995_ID_292752362770261791 | hypothetical protein | T4-like virus tail tube protein gp19, pfam06841,  E-value 1.05e-28 |
| fig\|564709.3.peg.2506 | Contig_37_length_117591_cov_438.095_ID_73116563114182238 | DNA primase, phage associated # P4-type | Phage- or plasmid-associated DNA primase [Mobilome: prophages, transposons];  E-value 3.24e-32 |
| fig\|564709.3.peg.3086 | Contig_45_length_94947_cov_442.235_ID_893854739005459 | phage protein | Nucleoside Triphosphate Pyrophosphohydrolase (EC 3.6.1.8) MazG-like domain  E-value 3.12e-05 |
| fig\|564709.3.peg.3307 | Contig_49_length_86236_cov_423.446_ID_97612250581065 | phage endolysin, Phage lysis modules | Glycoside hydrolase family 19 chitinase domain.  E-value 2.17e-04 |
| fig\|564709.3.peg.3537 | Contig_51_length_84369_cov_463.726_ID_1015790356944960 | putative prophage protein (ps3) | Uncharacterized phage-associated protein [Mobilome: prophages, transposons].  E-value 1.06e-20 |
| fig\|564709.3.peg.4364 | Contig_68_length_58146_cov_430.42_ID_13554321531251197 | hypothetical protein | Phage- or plasmid-associated DNA primase [Mobilome: prophages, transposons]  E-value 3.94e-35 |
| fig\|564709.3.peg.4366 | Contig_68_length_58146_cov_430.42_ID_13554321531251197 | phage integrase | Shufflon-specific DNA recombinase Rci and Bacteriophage Hp1_like integrase.  E-value 1.31e-17 |
| fig\|564709.3.peg.5051 | Contig_86_length_35161_cov_369.366_ID_17179687234735 | phage shock protein A | phage shock protein PspA; Provisional.  E-value 7.35e-34 |
| fig\|564709.3.peg.5052 | Contig_86_length_35161_cov_369.366_ID_17179687234735 | phage shock protein A | phage shock protein PspA; Provisional.  E-value 7.26e-24 |
| fig\|564709.3.peg.5237 | Contig_91_length_31916_cov_504.263_ID_18115950149101041 | phage protein | RNA ligase, DRB0094 family.  E-value 1.81e-64 |

**Table S8** **PHAST, PHASTER and VIRsorter prophage detection in *R*. *reptotaenium* AO1.** The detected prophage is considered as questionable or incomplete due to for example the lack of essential virus related genes, such as capsid, head, and tail genes.

| **#** | *R*. *reptotaenium* AO1, R1  **CDS position (contig 41)** | **PHASTER - BLAST hits** | **E-value** |
| --- | --- | --- | --- |
| 1 | 71377..72384 | PHAGE_Synech_ACG_2014f_NC_026927:  NAD-dependent epimerase/dehydratase  PP_02287 phage (gi815854730) | 3.37e-029 |
| 2 | 72417..73613 | PHAGE_Synech_ACG_2014f_NC_026927:  hypothetical protein  PP_02288 phage (gi815854731) | 7.15e-086 |
| 3 | 73622..74668 | PHAGE_Prochl_P_TIM68_NC_028955:  putative transketolase central region-containing protein  PP_02289 phage (gi971760363) | 1.78e-013 |
| 4 | 74671..75612 | PHAGE_Synech_S_SM2_NC_015279:  transketolase central region-containing protein  PP_02290 phage (gi326781943) | 1.52e-009 |
| 5 | 75648..76571 | hypothetical  PP_02291 | 0 |
| 6 | 76629..77624 | PHAGE_Synech_ACG_2014f_NC_026927:  GDP-D-mannose 4,6-dehydratase  PP_02292 phage (gi815854729) | 5.64e-040 |
| 7 | 77682..78776 | PHAGE_Caulob_Cr30_NC_025422:  D,D-heptose 7-phosphate kinase  PP_02293 phage (gi725949173) | 5.36e-044 |
| 8 | 78817..79218 | PHAGE_Caulob_Cr30_NC_025422:  phosphoheptose isomerase  PP_02294 phage (gi725949171) | 1.69e-015 |
| 9 | 79397..79978 | hypothetical PP_02295 | 0 |
| 10 | 79981..80703 | PHAGE_Entero_phi92_NC_023693:  Phi92_gp066  PP_02296 phage (gi726646999) | 1.68e-014 |
| 11 | 80715..81653 | PHAGE_Synech_ACG_2014f_NC_026927:  ADP-L-glycero-D-mannoheptose-6-epimerase  PP_02297 phage (gi815854739) | 5.82e-011 |
| 12 | 81674..81751 | hypothetical  PP_02298 | 0 |
| 13 | 81784..82971 | hypothetical  PP_02299 | 0 |
| 14 | 83072..83188 | hypothetical  PP_02300 | 0 |
| 15 | 83400..84692 | PHAGE_Plankt_PaV_LD_NC_016564:  ABC transporter  PP_02301 phage (gi371496158) | 8.98e-010 |
| **#** | *R*. *reptotaenium* AO1, R2  **CDS position (contig 72)** | **PHAST - BLAST hits** | **E-value** |
| 1 | 7385..7849 | PHAGE_Pseudo_F116_NC_006552:  DNA adenine methyltransferase  PP_00007; phage (gi56692911) | 7.00e-018 |
| 2 | 7869..8273 | PHAGE_Pseudo_F116_NC_006552:  DNA adenine methyltransferase  PP_00008; phage (gi56692911) | 1.00e-020 |
| 3 | 8280..9293 | PHAGE_Microc_Ma_LMM01_NC_008562:  lysozyme/metalloendopeptidase  PP_00009; phage (gi117530266) | 7.00e-017 |
| 4 | 9309..11027 | PHAGE_Parame_bursaria_Chlorella_virus_FR483_NC_008603:  hypothetical protein FR483_N733R  PP_00010; phage (gi155370831) | 9.00e-032 |
| 5 | 11328..12539 | PHAGE_Microc_Ma_LMM01_NC_008562:  transposase  PP_00011; phage (gi117530306) | 3.00e-104 |
| 6 | 12554..12685 | hypothetical  PP_00012 | 0 |
| 7 | 12936..13088 | hypothetical  PP_00013 | 0 |
| 8 | 13192..14520 | PHAGE_Cardio_polyomavirus_NC_020067:  major structural protein VP1  PP_00014; phage (gi440285304) | 2.00e-007 |
| # | *R*. *reptotaenium* AO1, R3  **CDS position (contig 93)** | **VIRsorter - BLAST hits** | **E-value** |
| 1 | 1..358 | hypothetical protein | - |
| 2 | 358..2963 | Phage_cluster_71; PFAM-AAA_25 (DNA repair protein) | 5.37e-25 |
| 3 | 3009..3455 | hypothetical protein | - |
| 4 | 3458..3919 | hypothetical protein | - |
| 5 | 4421..4942 | hypothetical protein | - |
| 6 | 4935..5204 | hypothetical protein | - |
| 7 | 5311..5814 | hypothetical protein | - |
| 8 | 6163..6423 | hypothetical protein | - |

**Table S9 PHAST, PHASTER and VIRsorter prophage detection in *Geitlerinema* sp. BBD_1991.** The detected prophage is considered as questionable or incomplete due to for example the lack of essential virus related genes, such as capsid, head, and tail genes.

| # | *Geitlerinema* sp. BBD_1991, G1  **CDS pos.** **(BBD_1000996)** | **PHAST - BLAST hits** | **E-value** |
| --- | --- | --- | --- |
|  | 337736..337747 | AttL AAGTGGCGTTTT | 0 |
| 1 | 338024..338470 | PHAGE_Lactob_phiPYB5_NC_027982: putative integrase; PP_00353; phage (gi937456185) | 9.00e-005 |
| 2 | 338504..338869 | PHAGE_Dinoro_IMEphi4_NC_024367: putative host-like protein; PP_00354; phage (gi658311036) | 7.00e-006 |
| 3 | 339100..340053 | PHAGE_Synech_S_SKS1_NC_020851: GDP-L-fucose synthase; PP_00355; phage (gi472340899) | 5.00e-062 |
| 4 | 340160..341236 | PHAGE_Synech_S_SKS1_NC_020851: GDP-D-mannose 4,6-dehydratase; PP_00356; phage (gi472340900) | 5.00e-109 |
| 5 | 341422..342153 | glycosyl transferase [Oscillatoria acuminata PCC 6304]. gi\|428211603\|ref\|YP_007084747.1\|; PP_00357 | 9.00e-098 |
| 6 | 342220..343476 | PHAGE_Synech_ACG_2014f_NC_026927: group 1 glycosyl transferase; PP_00358; phage (gi815854524) | 5.00e-009 |
| 7 | 343579..344703 | PHAGE_Bathyc_BpV1_NC_014765: hypothetical protein; PP_00359; phage (gi313768026) | 6.00e-049 |
|  | 357614..357625 | AttR AAGTGGCGTTTT | 0 |
| # | *Geitlerinema* sp. BBD_1991, G2  **CDS pos.** **(BBD_1001009)** | **PHAST - BLAST hits** | **E-value** |
| 1 | 2477808..2478164 | PHAGE_Helico_phiHP33_NC_016568: transposase; PP_02553; phage (gi371671361) | 3.00e-015 |
| 2 | 2478257..2479516 | PHAGE_Microc_Ma_LMM01_NC_008562: transposase; PP_02554; phage (gi117530202) | 2.00e-045 |
| 3 | 2479573..2482482 | PHAGE_Ectoca_siliculosus_virus_1_NC_002687: EsV-1-65; PP_02555; phage (gi13242537) | 5.00e-040 |
| 4 | 2482470..2482589 | hypothetical; PP_02556 | 0 |
| 5 | 2482597..2482731 | hypothetical; PP_02557 | 0 |
| 6 | 2482789..2482908 | hypothetical; PP_02558 | 0 |
| 7 | 2483196..2483342 | hypothetical; PP_02559 | 0 |
| 8 | 2483336..2484139 | PHAGE_Synech_S_SKS1_NC_020851: cyanobacterial phosphoribosylglycinamide formyltransferase; PP_02560; phage (gi472340960 | 2.00e-036 |
| 9 | 2484180..2484779 | PHAGE_Prochl_P_SSM7_NC_015290: orotate phosphoribosyltransferase; PP_02561; phage (gi326784523) | 3.00e-032 |
| 10 | 2485011..2486813 | PHAGE_Cyanop_S_TIM5_NC_019516: virion structural protein; PP_02562; phage (gi422936314) | 3.00e-016 |
| # | *Geitlerinema* sp. BBD_1991, G3  **CDS pos.** **(BBD_1001028)** | **PHAST - BLAST hits** | **E-value** |
| 1 | 4406431..4408458 | PHAGE_Cyanop_NATL2A_133_NC_016659: hypothetical protein; PP_04556; phage (gi372217849) | 2.00e-030 |
| 2 | 4408486..4409214 | phage tail protein [Calothrix sp. PCC 7507]. gi\|427718458\|ref\|YP_007066452.1\|; PP_04557 | 8.00e-030 |
| 3 | 4409211..4410185 | hypothetical protein Anacy_0193 [Anabaena cylindrica PCC 7122]. gi\|440679915\|ref\|YP_007154710.1\|; PP_04558 | 8.00e-065 |
| 4 | 4410236..4413910 | PHAGE_Bacill_BCD7_NC_019515: putative baseplate J family protein; PP_04559; phage (gi422936037) | 5.00e-016 |
| 5 | 4413999..4414385 | PHAGE_Synech_ACG_2014e_NC_026928: base plate wedge subunit; PP_04560; phage (gi815854880) | 1.00e-010 |
| 6 | 4414418..4414843 | PAAR motif protein [Teredinibacter turnerae T7901]. gi\|254787612\|ref\|YP_003075041.1\|; PP_04561 | 3.00e-046 |
| 7 | 4414853..4415503 | PHAGE_Campyl_CP30A_NC_018861: putative baseplate hub and tail lysozyme; PP_04562; phage (gi410493030) | 9.00e-008 |
| 8 | 4415500..4416558 | PHAGE_Bacill_BCD7_NC_019515: hypothetical protein; PP_04563; phage (gi422936041) | 2.00e-010 |
| 9 | 4416577..4417227 | PHAGE_Salmon_ViI_NC_015296: conserved uncharacterised protein; PP_04564; phage (gi326804610) | 4.00e-005 |
| 10 | 4417224..4418816 | PHAGE_Molliv_sibericum_NC_027867: hypothetical protein; PP_04565; phage (gi927594325) | 3.00e-009 |
| 11 | 4418829..4419368 | PHAGE_Bacill_BCD7_NC_019515: putative tail tube protein 2; PP_04566; phage (gi422936047) | 4.00e-012 |
| 12 | 4419384..4419521 | hypothetical protein Anacy_0201 [Anabaena cylindrica PCC 7122]. gi\|440679923\|ref\|YP_007154718.1\|; PP_04567 | 4.00e-010 |
| 13 | 4419527..4419904 | hypothetical protein Lepto7376_0625 [Leptolyngbya sp. PCC 7376]. gi\|427722594\|ref\|YP_007069871.1\|; PP_04568 | 1.00e-045 |
| 14 | 4419904..4420365 | PHAGE_Bacill_BCD7_NC_019515: putative tail tube protein 2; PP_04569; phage (gi422936047) | 2.00e-018 |
| 15 | 4420381..4421694 | PHAGE_Bacill_BCD7_NC_019515: putative tail sheath protein; PP_04570; phage (gi422936048) | 2.00e-026 |
| 16 | 4421802..4423052 | PHAGE_Bacill_BCD7_NC_019515: putative tail sheath protein; PP_04571; phage (gi422936048) | 9.00e-038 |
| # | *Geitlerinema* sp. BBD_1991, G4  **CDS pos.** **(BBD_1001072)** | **PHAST - BLAST hits** | **E-value** |
| 1 | 5824500..5825465  BBD_1001072 | PHAGE_Chryso_virus_NC_028094: putative glycosyltransferase; PP_06057; phage (gi939177431) | 1.00e-009 |
| 2 | 5825458..5825685 | hypothetical protein Cal7507_2917 [Calothrix sp. PCC 7507]. gi\|427718171\|ref\|YP_007066165.1\|; PP_06058 | 9.00e-014 |
| 3 | 5825691..5825807 | hypothetical; PP_06059 | 0 |
| 4 | 5826905..5827384 | glycosyl transferase family 2 [Oscillatoria nigro-viridis PCC 7112]. gi\|428319715\|ref\|YP_007117597.1\|; PP_06060 | 5.00e-026 |
| 5 | 5827381..5827593  BBD_1001073 | hypothetical; PP_06061 | 0 |
| 6 | 5828156..5829235 | PHAGE_Ostreo_2_NC_028091: hypothetical protein; PP_06062; phage (gi939177229) | 8.00e-011 |
| 7 | 5829191..5829349 | hypothetical; PP_06063 | 0 |
| 8 | 5829364..5829681 | PHAGE_Megavi_chiliensis_NC_016072: thioredoxin-like protein; PP_06064; phage (gi363540574) | 2.00e-008 |
| 9 | 5829758..5829895 | hypothetical; PP_06065 | 0 |
| 10 | 5829878..5830357 | PHAGE_Pandor_salinus_NC_022098: guanine deaminase; PP_06066; phage (gi531037005 | 4.00e-025 |
| 11 | 5830479..5831102 | PHAGE_Microm_12T_NC_020864: hypothetical protein; PP_06067; phage (gi472342811 | 7.00e-007 |
| 12 | 5831263..5832063 | PHAGE_Entero_ST104_NC_005841: ORF19; PP_06068; phage (gi46358666) | 8.00e-018 |
| 13 | 5832192..5833109 | type 11 methyltransferase [Calothrix sp. PCC 7507]. gi\|427718977\|ref\|YP_007066971.1\|; PP_06069 | 4.00e-106 |
| 14 | 5833106..5834035 | iron permease FTR1 [Oscillatoria nigro-viridis PCC 7112]. gi\|428315250\|ref\|YP_007113132.1\|; PP_06070 | 3.00e-102 |
| 15 | 5834481..5835026 | PHAGE_Rubell_virus_NC_001545: non-structural polyprotein; PP_06071; phage (gi336284683) | 2.00e-012 |
| 16 | 5835195..5835266 | tRNA | 0 |
| 17 | 5835298..5835510 | periplasmic solute binding protein [Geitlerinema sp. PCC 7407]. gi\|428224031\|ref\|YP_007108128.1\|; PP_06072 | 4.00e-010 |
| 18 | 5835859..5836659 | hypothetical protein PCC7424_4846 [Cyanothece sp. PCC 7424]. gi\|218441742\|ref\|YP_002380071.1\|; PP_06073 | 5.00e-084 |
| 19 | 5836794..5837648 | biotin-(acetyl-CoA-carboxylase) ligase BirA [Oscillatoria acuminata PCC 6304]. gi\|428211981\|ref\|YP_007085125.1\|; PP_06074 | 2.00e-057 |
| 20 | 5837759..5838928  5837696..5838928 | PHAGE_Lactob_phig1e_NC_004305: minor capsid protein; PP_06075; phage (gi23455811)  BLASTp: Peptidase, M23/M37 family [*Phormidium* sp. OSCR],  Den Uyl., 2016: BBD_100107313, Membrane proteins related to metalloendopeptidases COG0739 | 1.00e-016  2.00e-124 |
| 21 | 5839022..5839315 | PROPHAGE_Escher_Sakai: putative transposase TnA; PP_06076; phage (gi15832531 | 8.00e-020 |
| 22 | 5839572..5839844 | PHAGE_Geobac_E3_NC_029073: transposase; PP_06077; phage (gi985758480) | 4.00e-007 |
| 23 | 5839852..5840331 | PHAGE_Geobac_E3_NC_029073: transposase; PP_06078; phage (gi985758480) | 2.00e-027 |
| 24 | 5840636..5840941  BBD_1001074 | PROPHAGE_Deinoc_R1: serine protease; PP_06079; phage (gi15807944) | 5.00e-007 |
| 25 | 5840913..5841986 | PROPHAGE_Deinoc_R1: serine protease; PP_06080; phage (gi15807944) | 0.00007 |
| 26 | 5842143..5843258 | subtilase family protease [Dactylococcopsis salina PCC 8305]. gi\|428780017\|ref\|YP_007171803.1\|; PP_06081 | 4.00E-082 |
| 27 | 5843285..5843407 | hypothetical; PP_06082 | 0 |
| 28 | 5843519..5843752 | hypothetical; PP_06083 | 0 |
| 29 | 5843783..5843911 | hypothetical; PP_06084 | 0 |
| 30 | 5844106..5845404 | PHAGE_Strept_20617_NC_023503: enolase; PP_06085; phage (gi588295080) | 8.00e-136 |
| **#** | *Geitlerinema* sp. BBD_1991, G1  **CDS pos. (BBD_1000996)** | **PHASTER - BLAST hits** | **E-value** |
| 1 | 136544..136555 | attL | 0.0 |
| 2 | 136832..137278 | PHAGE_Bacill_Fah_NC_007814: site-specific serine recombinase; PP_00294; phage (gi89152504) | 9.48e-06 |
| 3 | 137312..137677 | PHAGE_Dinoro_DFL12phi1_NC_024367: putative host-like protein; PP_00295; phage (gi658311036) | 6.82e-09 |
| 4 | 137908..138861 | PHAGE_Synech_S_SKS1_NC_020851: GDP-L-fucose synthase; PP_00296; phage (gi472340899) | 2.59e-76 |
| 5 | 138968..140044 | PHAGE_Synech_S_SKS1_NC_020851: GDP-D-mannose 4,6-dehydratase; PP_00297; phage (gi472340900) | 5.17e-139 |
| 6 | 140230..140961 | hypothetical; PP_00298 | 0.0 |
| 7 | 141028..142284 | PHAGE_Synech_ACG_2014f_NC_026927: N/A; PP_00299; phage (gi815854524) | 7.99e-10 |
| 8 | 142366..143511 | PHAGE_Synech_ACG_2014f_NC_026927: N/A; PP_00300; phage (gi815854731) | 3.21e-19 |
| 9 | 156422..156433 | attR | 0.0 |
| # | *Geitlerinema* sp. BBD_1991, G3  **CDS pos.** **(BBD_1001028)** | **PHAST - BLAST hits** | **E-value** |
| 1 | 59826..61865 | PHAGE_Cyanop_NATL2A_133_NC_016659: hypothetical protein; PP_03742; phage (gi372217849) | 8.97e-27 |
| 2 | 61881..62588 | hypothetical; PP_03743 | 0.0 |
| 3 | 62606..63580 | hypothetical; PP_03744 | 0.0 |
| 4 | 63631..67305 | PHAGE_Bacill_BCD7_NC_019515: putative baseplate J family protein; PP_03745; phage (gi422936037) | 1.49e-17 |
| 5 | 67394..67780 | PHAGE_Synech_ACG_2014e_NC_026928: base plate wedge subunit; PP_03746; phage (gi815854880) | 8.45e-14 |
| 6 | 67813..68214 | hypothetical; PP_03747 | 0.0 |
| 7 | 68248..68829 | PHAGE_Campyl_NCTC12673_NC_015464: gp5 baseplate hub subunit and tail lysozyme; PP_03748; phage (gi332672341) | 7.30e-08 |
| 8 | 68895..69953 | PHAGE_Bacill_BCD7_NC_019515: hypothetical protein; PP_03749; phage (gi422936041) | 3.37e-11 |
| 9 | 69972..70571 | PHAGE_Salmon_ViI_NC_015296: conserved uncharacterised protein; PP_03750; phage (gi326804610) | 4.24e-07 |
| 10 | 70619..72187 | hypothetical; PP_03751 | 0.0 |
| 11 | 72224..72763 | PHAGE_Bacill_BCD7_NC_019515: putative tail tube protein 2; PP_03752; phage (gi422936047) | 2.74e-14 |
| 12 | 72779..72859 | hypothetical; PP_03753 | 0.0 |
| 13 | 72922..73221 | hypothetical; PP_03754 | 0.0 |
| 14 | 73299..73724 | PHAGE_Bacill_BCD7_NC_019515: putative tail tube protein 2; PP_03755; phage (gi422936047) | 2.24e-22 |
| 15 | 73776..75089 | PHAGE_Bacill_BCD7_NC_019515: putative tail sheath protein; PP_03756; phage (gi422936048) | 9.98e-30 |
| 16 | 75197..76447 | PHAGE_Bacill_BCD7_NC_019515: putative tail sheath protein; PP_03757; phage (gi422936048) | 3.98e-42 |
| **#** | *Geitlerinema* sp. BBD_1991, G5  **CDS pos. (BBD_1001065)** | **VIRsorter - BLAST hits** | **E-value** |
| 1 | 369..650 | PFAM-DUF4258 | 5.80e-22 |
| 2 | 656..883 | hypothetical protein | - |
| 3 | 946..1206 | hypothetical protein | - |
| 4 | 1417..1635 | hypothetical protein | - |
| 5 | 1727..3124 | hypothetical protein | - |
| 6 | 3114..4043 | hypothetical protein  BLASTp: metalloendopeptidase-like membrane protein [*Oscillatoria* *acuminata*], WP_015152078.1  Den Uyl., 2016: BBD_10010657, membrane proteins related to metalloendopeptidases, COG0739 | -  2.00e-19 |
| 7 | 4084..4608 | hypothetical protein | - |
| 8 | 4613..4909 | hypothetical protein | - |
| 9 | 4909..5232 | hypothetical protein | - |
| 10 | 5196..6245 | hypothetical protein | - |
| 11 | 6245..7012 | hypothetical protein | - |

**Fig. S1 De Bruijn graph connection of contig 93**. De Bruijn graph was visualised with the software bandage (Table S1). Contig 93 can be connected to several other contigs (contig number in figure with coverage). A potential prophage could be spread over the adjacent contigs. Black marks on contig 93 indicate CRISPR-cas target loci.


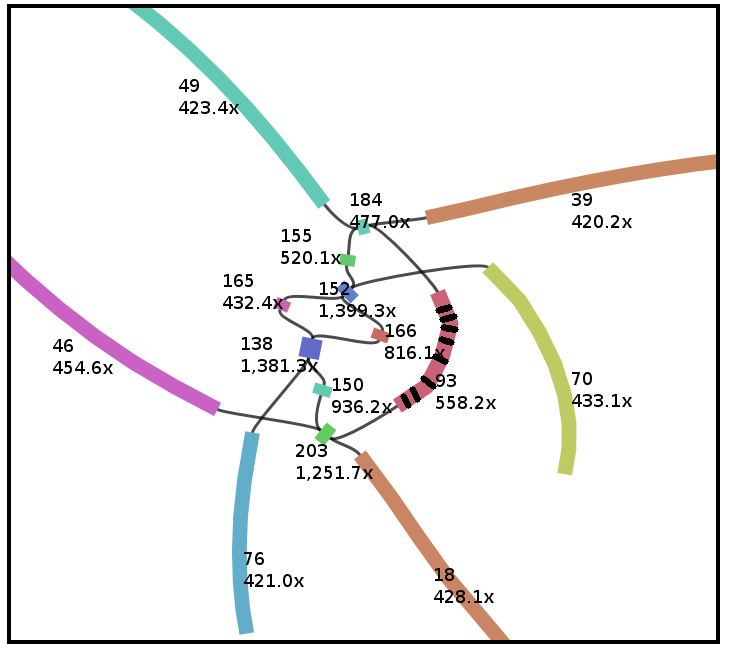

Supplement: Supplementary file 1 [file Data_Sheet_1.DOCX]
